# Superconducting Sn-Intercalated TaSe_2_: Structural Diversity Obscured by Routine Characterization Techniques

**DOI:** 10.1021/jacs.5c06808

**Published:** 2025-10-21

**Authors:** Brenna C. Bierman, Gillian Nolan, Hongrui Ma, Ying Wang, Pinshane Huang, Daniel A. Rhodes

**Affiliations:** † Department of Chemistry, 5228 University of Wisconsin-Madison, Madison, Wisconsin 53706, United States; ‡ The Grainger College of Engineering, Department of Materials Science and Engineering, 14589 University of Illinois Urbana−Champaign, Urbana, Illinois 61801, United States; § Department of Electrical and Computer Engineering, 5228 University of Wisconsin-Madison, Madison, Wisconsin 53706, United States; ∥ Department of Physics, 5228 University of Wisconsin-Madison, Madison, Wisconsin 53706, United States; ⊥ Department of Materials Science and Engineering, 5228 University of Wisconsin-Madison, Madison, Wisconsin 53706, United States; # Materials Research Laboratory, 14589 University of Illinois Urbana−Champaign, Urbana, Illinois 61801, United States

## Abstract

Using Sn-intercalated TaSe_2_ as a model system, we demonstrate the presence of structural heterogeneity captured by single-crystal X-ray diffraction (SCXRD) and scanning transmission electron microscopy (STEM) that eludes the routine characterization techniques of powder X-ray diffraction, Raman spectroscopy, and electronic transport measurements. From a single growth composition (1:1:2 Sn:Ta:Se), we obtained crystals diverse in stoichiometry and structure, with near-continuous intercalation for Sn_*x*_TaSe_2_ from 0 ≲ *x* ≲ 1. Using SCXRD, we found global structural diversity, identifying three new structure types: Sn_0.18_TaSe_2.0_/Sn_0.08_TaSe_1.96_ (*R*3*m*), Sn_0.16_TaSe_2.0_ (*P*6_3_/*mmc*), and Sn_1.2_TaSe_1.9_ (*Fmm*2). Using STEM, we observed local structural diversity, manifested as regions of highly variable stacking within a single crystal. In contrast, powder X-ray diffraction did not resolve all observed global structures. Raman spectroscopy was unable to distinguish between different structures or compositions in the standard measurement range. Electronic transport measurements showed consistent superconductivity and charge density wave behavior irrespective of Sn-intercalation amount. Our results indicate that routine approaches to characterization of intercalated transition metal dichalcogenides may be inadequate for capturing the diversity of this family of materials, highlighting the need for high-resolution structural characterization when examining the properties of van der Waals-layered compounds.

## Graphical abstract

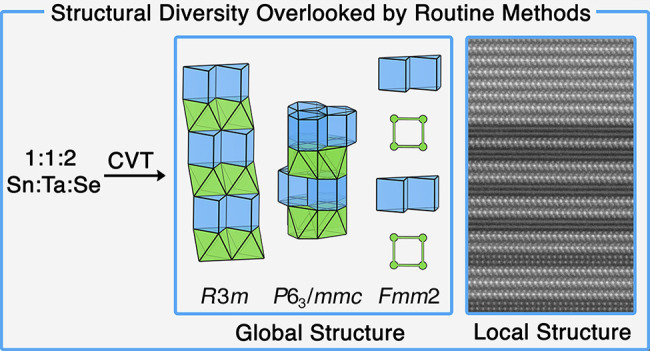

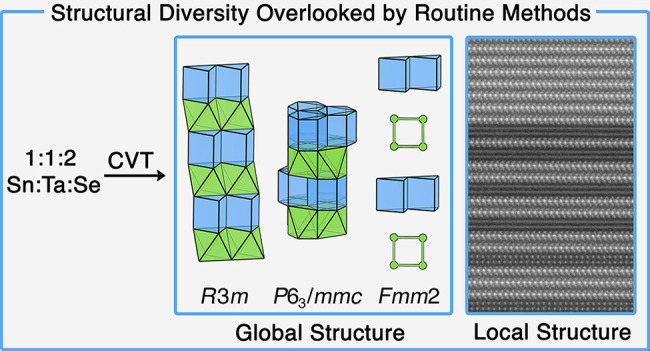

## Introduction

Transition metal dichalcogenides (TMDs) are a class of van der Waals (vdW) layered compounds known for their unusual electronic, optical, and physical properties, including superconductivity, charge density waves (CDW), and exfoliability.^1^ Intercalation of TMDs with guest atoms can introduce or enhance properties such as magnetism^2,3^ or superconductivity.^4,5^ Intercalated atoms generally occupy an octahedral, tetrahedral, or trigonal prismatic void, forming distinct layers in the structures.^6,7^ The intercalated atoms typically exhibit substantial positional and occupational disorder.^8–11^ These nonperiodic features can result in regions of local order in a crystal that may not be representative of the global crystal structure, complicating the determination of intercalated TMD structures.^12–15^

While metal-intercalated TMDs are well-studied,^6,16^ much of the structural understanding of these compounds originates from characterization techniques that cannot resolve all structural details^17,18^ – particularly powder X-ray diffraction (PXRD), which compresses three-dimensional diffraction patterns into one-dimension, resulting in information loss.^19–22^ Some TMD structures have been elucidated using more information-rich techniques, such as single-crystal X-ray diffraction (SCXRD).^23–26^ However, many foundational reports on TMD structures employed now antiquated SCXRD methods, including determination of structures by hand from physical film.^27,28^ SCXRD has since advanced, enabling the collection of more reflections and the determination of more accurate reflection intensities, but many structures have not been reexamined with modern techniques. As a result, reported structures may be incorrect or incomplete, potentially stymieing investigation of structure–property relationships.^29–31^

For example, recent reevaluation of the structure of SrTa_2_S_5_ led to discovery of its unusual electronic characteristics.^31^ Early investigations of SrTa_2_S_5_ reported a $\sqrt{\left(28\right)}a\times \sqrt{\left(28\right)}a$ supercell obtained using PXRD, but did not resolve a complete structure.^32,33^ Devarakonda et al. reexamined and solved the complete crystal structure, clarifying the incommensurate modulation of SrTa_2_S_5_.^31^ This modulation was key to discovering the striped electronic behavior of SrTa_2_S_5_, suggesting this compound hosts a pair-density wave state.

To highlight the potential complexity of and demonstrate an approach to more completely characterize intercalated TMD structures, we examined global and local structures of Sn-intercalated TaSe_2_ crystals. SnTaSe_2_ is predicted to be a topological superconductor candidate isostructural with PbTaSe_2_,^34^ a non-centrosymmetric superconductor known to host topological Dirac surface states.^35,36^ To our knowledge, three studies of Sn-intercalated TaSe_2_ have been detailed.^37–40^ All reports rely primarily or exclusively on PXRD for global structural characterization. None of the reports used SCXRD or other long-range, high-resolution structural characterization methods. The earliest study reported a stoichiometry (SnTaSe_2_) and lattice parameters (*a* = 3.42 Å, *c* = 18.38 Å) obtained by treating PXRD reflections as SCXRD reflections and constraining refinement using several assumptions about the structure, including the range of possible space groups.^37,38^ The stoichiometry was possibly checked with density measurements, but notably the SnTaSe_2_ phase does not have Mössbauer data to support the structural assignment as provided for all other phases in the report. The second study reports a stoichiometry of Sn_0.5_TaSe_2_ that appears to result from a misinterpretation of energy dispersive spectroscopy (EDS) data.^39^ The authors propose a structure for Sn_0.5_TaSe_2_ primarily by matching three (00*l*) peaks to those of similar phases described in historic reports.^18,38,41^ The most recent report provides a unit-cell sized-view of local order obtained by cross-sectional high-resolution scanning transmission electron microscopy (STEM). The proposed stoichiometry, 2Sn-2TaSe_2_, is supported by both EDS and X-ray photoelectron spectroscopy (XPS) data. However, direct analysis of global structure by an information-rich technique was not conducted. Global structure was characterized mainly through comparison of select experimental PXRD peaks to those generated for a potential *R*3*m* structure.^40^ All three of these reports imply that the synthetic Sn-intercalated TaSe_2_ products are uniform in stoichiometry and structure; we find this system to be significantly more complex.

Here, we use SCXRD to identify three new Sn_*x*_TaSe_2_ crystal structures (0 ≲ *x* ≲ 1), originating from a single growth composition (1:1:2, Sn:Ta:Se). While significant global structural diversity is represented among these phases, the full range of possible Sn_*x*_TaSe_2_ structures may not be captured by those reported here. Using STEM, we identified regions of spontaneous variable local stacking order within a single crystal of Sn_*x*_TaSe_2_, demonstrating appreciable local structural diversity. We found that common characterization methods, such as PXRD, Raman spectroscopy, and electronic transport, were unable to capture the heterogeneity of the Sn_*x*_TaSe_2_ system. PXRD only resolved two of the three structures identified by SCXRD, and lacked the specificity needed to unambiguously characterize some structural details, such as stoichiometry, for the phases. Whereas stoichiometry and structure varied considerably, the Raman peaks in the standard measurement range, superconducting critical temperature (*T*_c_), and charge density wave onset temperature (*T*_CDW_) did not differ significantly across the samples measured. Our findings illustrate that a surprising amount of structural and stoichiometric diversity can emerge from a single growth composition and demonstrate the need for high-resolution approaches to intercalated TMD structure determination and verification.

## Results and Discussion

### Structural Characterization

Sn_*x*_TaSe_2_ crystals were synthesized by chemical vapor transport (see Supporting Information for details) and analyzed using SCXRD. All crystals screened were twinned, including those that were visually pristine and a few microns in length (Figure S1). Screening ≳100 crystals per data set was required to find a specimen where the major twin component contributed ≥90*%* of the reflections. Non-merohedral twin domains could be a common, intrinsic feature of bulk vdW-layered TMDs, found in both intercalated and non-intercalated TMDs (Figure S2).^42^ Extrinsic factors, such as mechanical strain induced by cutting the crystals, could also introduce twin components.^43–45^ Both mechanisms may contribute to the observed SCXRD behavior. Additionally, merohedral twinning might be common in Sn_*x*_TaSe_2_, occurring in two of the four SCXRD data sets.

Figure 1 presents the observed Sn_*x*_TaSe_2_ crystal structures, with refinement details provided in Table 1 and precession images in Figures S3–S6. The first structure was observed in two data sets, corresponding to two different stoichiometries, Sn_0.18_TaSe_2.0_ and Sn_0.08_TaSe_1.96_, in the same three-layer stacking configuration (*R*3*m*). The structure is analogous to 3*R*-Ta_1.11_Se_2_ (*R*3*m*).^46^ In Ta_1.11_Se_2_, the octahedral voids between the three layers of Ta-centered trigonal prisms are partially occupied by Ta (11%). Similarly, in Sn_0.18_TaSe_2.0_ and Sn_0.08_TaSe_1.96_, the octahedral voids between TaSe_2_ layers contain a Sn site occupied 18% or 8% respectively. Se occupies two distinct sites in both 3*R*-Sn_*x*_TaSe_2_ structures, forming the vertices of the trigonal prismatic Ta coordination polyhedra. All of the Ta and Se sites are fully or near fully occupied without positional disorder. Both data sets for the first structure exhibit merohedral twinning. Merohedral twin domains produce perfectly coinciding reflections, enabling determination of twin contributions from refinement.^47,48^ Data set one features an inversion twin component: (1 0 0/0 1 0/0 0 1), 49(17)%. In data set two, the twin component represents a −180° rotation around c*: (1 0 0/1 1 0/0 0 1), 25.2­(7)%.

**Figure 1:**
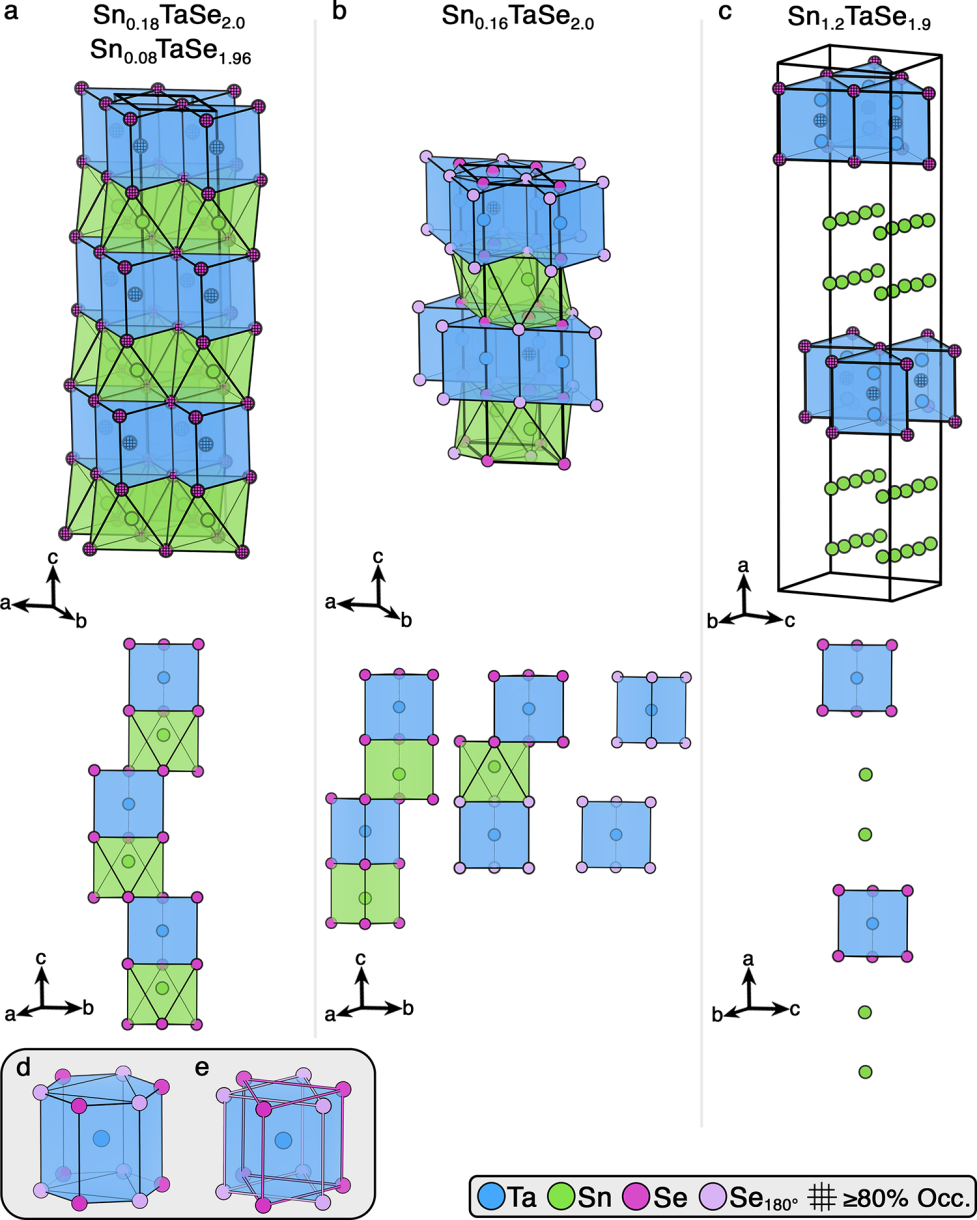
Crystal structures (top), idealized stacking (middle), and rotational disorder (bottom) for Sn_*x*_TaSe_2_. (a) Sn_0.18_TaSe_2.0_ (structure one, data set one) and Sn_0.08_TaSe_1.96_ (structure one, data set two), (b) Sn_0.16_TaSe_2.0_ (structure two), (c) Sn_1.2_TaSe_1.9_ (structure three), (d) Ta coordination for Sn_0.16_TaSe_2.0_ as depicted in panel (b), and (e) Ta coordination for Sn_0.16_TaSe_2.0_ highlighting the two rotated trigonal prisms arrangements. ≥80% occupied sites are designated with a grid pattern. Se_180°_ denotes the Se site attributed to a 180° rotation of the major Ta-centered coordination polyhedron.

**Table 1:** Structural Data and Refinement for Sn_*x*_TaSe_2_ Crystals

**empirical formula**	**Sn**_**0.18**_ **TaSe**_**2.0**_	**Sn**_**0.08**_ **TaSe**_**1.96**_	**Sn**_**0.16**_ **TaSe**_**2.0**_	**Sn**_**1.2**_ **TaSe**_**1.9**_
radiation	Mo Kα (λ = 0.71069)	Mo Kα (λ = 0.71069)	Mo Kα (λ = 0.71069)	Mo Kα (λ = 0.71069)
*T*/K	293	293	293	293
crystal system	trigonal	trigonal	hexagonal	orthorhombic
space group	*R*3*m*	*R*3*m*	*P*6_3_/*mmc*	*Fmm*2
*a*/Å	3.4417(1)	3.4409(8)	3.44110(7)	24.734(4)
*b*/Å	3.4417(1)	3.4409(8)	3.44110(7)	3.4193(4)
*c*/Å	18.8554(9)	18.997(5)	12.622(1)	6.0307(7)
volume/Å^3^	193.425(15)	194.78(10)	129.435(12)	510.03(12)
*Z*	3	3	2	4
ρ_calc_ g/cm^3^	9.118	8.825	9.176	6.463
μ/mm^–1^	70.853	70.161	71.554	42.678
F(000)	442.0	431.0	298.0	829.0
crystal size/mm^3^	0.03 × 0.02 × 0.01	0.01 × 0.007 × 0.001	0.0206 × 0.0133 × 0.001	0.0319 × 0.0188 × 0.0008
2θ range for data collection/°	6.482–57.16	6.434–58.932	6.456–59.988	3.294–51.564
index ranges	–4 ≤ *h* ≤ 4	–4 ≤ *h* ≤ 4	–4 ≤ *h* ≤ 4	–27 ≤ *h* ≤ 30
	–3 ≤ *k* ≤ 4	–4 ≤ *k* ≤ 4	–4 ≤ *k* ≤ 4	–4 ≤ *k* ≤ 4
	–24 ≤ *l* ≤ 23	–25 ≤ *l* ≤ 25	–17 ≤ *l* ≤ 16	–7 ≤ *l* ≤ 7
reflections collected	1029	1249	1563	1122
independent reflections	168 [*R*_int_ = 0.0342, *R*_σ_ = 0.0247]	184 [*R*_int_ = 0.0300, *R*_σ_ = 0.0194]	100 [*R*_int_ = 0.0731, *R*_σ_ = 0.0277]	296 [*R*_int_ = 0.0652, *R*_σ_ = 0.0576]
data/restraints/parameters	168/0/14	184/0/14	100/1/19	296/0/12
goodness-of-fit on *F* ^2^	1.276	1.085	1.320	1.207
final *R* indexes [*I* > 2σ(*I*)]	*R*_1_ = 0.0266,	*R*_1_ = 0.0148,	*R*_1_ = 0.0441,	*R*_1_ = 0.0943,
	*wR*_2_ = 0.0686	*wR*_2_ = 0.0342	*wR*_2_ = 0.1076	*wR*_2_ = 0.2429
final *R* Indexes [all data]	*R*_1_ = 0.0266,	*R*_1_ = 0.0148,	*R*_1_ = 0.0469,	*R*_1_ = 0.1027,
	*wR*_2_ = 0.0686	*wR*_2_ = 0.0342	*wR*_2_ = 0.1090	*wR*_2_ = 0.2498
largest diff. peak/hole/eÅ^–3^	2.36/–1.57	0.67/–0.80	4.20/–5.40	4.89/–2.60
Flack parameter (*x*)	0.49(17)	–0.03(8)		0.1(4)
Hooft parameter (*y*)	–0.01(4)	–0.07(3)		–0.11(6)

Structure two, Sn_0.16_TaSe_2.0_ (*P*6_3_/*mmc*) (Figure 1b), features a two-layer stacking arrangement of Ta-centered trigonal prisms, with interstitial Sn between TaSe_2_ layers (8%). Sn_0.16_TaSe_2.0_ appears to exhibit rotational disorder (Figure 1d-e). Se is disordered across three sites. Two are ∼50% occupied, forming vertices for two trigonal prismatic arrangements around the major Ta site that appear 180° rotated around the *c* axis, similar to the rotational disorder previously reported in 4*H*-Ta_1.03_Se_2_.^49^ The disorder is not necessarily a 180° rotation. The observed sites could match a 60° rotation, 120° rotation, or a mirror plane. The rotational disorder creates three distinct interlayer environments (Figure 1b idealized stacking). Two of these stacking arrangements can accommodate Sn, in trigonal prismatic or octahedral voids. The tetrahedral voids between two Se_180°_ layers are presumably vacant; if both Se_180°_ and Sn were present, the Se–Sn distance would be unreasonably short (1.2223(1) Å) (Figure S7). Ta appears disordered across two sites (88% and 11%). The third Se site (13%), or possibly the Se_180°_ site, appears to form a trigonal prismatic coordination environment around the second Ta site. Similar disordered positions have been reported before, and may be indicative of stacking faults.^50,51^

The final structure, Sn_1.2_TaSe_1.9_ (*Fmm*2) (Figure 1c), features the longest unit cell of the three structures, but contains only two TaSe_2_ layers. The space between TaSe_2_ layers contains two layers of two diffuse channels of Sn atoms, similar to the Sn layers reported in 2Sn-2TaSe_2_.^40^ The channels are composed of three distinct partially occupied Sn sites (15%, 16.5%, and 16%). Positional disorder appears in Ta, with occupancy split between two sites (95% and 5%). Similar disorder was recently reported in Ba_0.75_ClTaSe_2_,^52^ and might be explained by the presence of stacking faults.^53–55^ We note that our SCXRD analysis leaves some uncertainty in the structures. A discussion of this uncertainty is included in the Supporting Information. Elemental analysis provides additional support for the presence of Sn, in the form of XPS (Figure S8) and EDS (Figure S9–S10) data.

The three structures resolved by SCXRD represent distinct long-range orders (μm scale) observed in the Sn_*x*_TaSe_2_ system. To examine the local ordering (nm scale), we collected high-resolution cross-sectional STEM. The samples analyzed by STEM were taken from the same bulk flakes as the specimens corresponding to structures two and three, but collection did not use the exact section of crystal analyzed by SCXRD. Figure 2a shows layers of Ta-centered trigonal prisms, with diffuse intercalants between layers. In Figure 2b, we observe layers of Ta-centered trigonal prisms alternating with two channels of Sn. In other regions, individual Sn atoms can be resolved (Figure S11). EDS of select regions were collected and support the presence of these features (Figures S12 and S13). EDS did not definitively identify the intercalants shown in Figure 2a, but they could plausibly represent either Sn or Ta. The regions of periodic behavior demonstrate that consistent structures exist in the phase. We observe relatively consistent stacking, with expected local disorder represented in features such as stacking faults and inconsistent intercalant presence.

**Figure 2:**
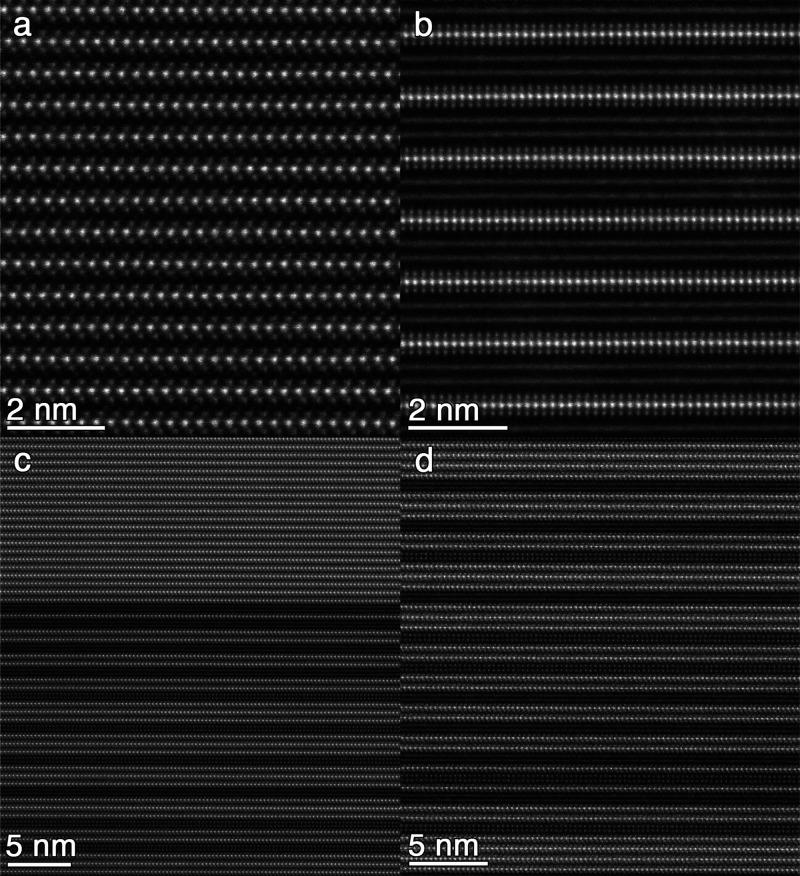
High-resolution cross-sectional STEM for Sn_*x*_TaSe_2_. Periodic regions of (a) Ta-centered trigonal prisms with visible intercalants and (b) Ta-centered trigonal prisms alternated with two channels of Sn. (c) Transition from periodic to variable stacking. (d) Variability in number of TaSe_2_ layers between Sn channels. STEM images for panels (a), (c), and (d) were obtained from the flake corresponding to structure two. STEM image for panel (b) was obtained from the crystal corresponding to structure three. All images show the out-of-plane direction as vertical.

Our STEM data revealed a second dimension of structural diversity in Sn_*x*_TaSe_2_. Periodic stacking appears to spontaneously transition to variable stacking within a single crystal (Figure 2c). Within the variable stacking region, we observe between one to four TaSe_2_ layers alternated with two layers of Sn channels (Figure 2d). In this instance, the variable stacking region spans approximately 150 nm (Figure S14), terminating in one μm of periodic stacking of two layers of TaSe_2_ interleaved with two layers of Sn channels (Figure S15). This stacking order spans from the end of the variable stacking region to the surface of the crystal (Figure S16). A similar stacking order is observed in 2Sn-2TaSe_2_, but is distinct in the relative orientation of TaSe_2_ layers.^40^ The formation of multiple stacking polymorphs within a sample is well established in layered materials, particularly those synthesized by deposition.^56–61^ Our results expand upon these previous reports by evidencing disparate structural motifs within a single bulk-grown vdW crystal. Bulk properties measurements are typically ascribed to a single structural phase. That several distinct structures can coexist in a single crystal highlights the importance of considering both the local and global order when examining structure–property relationships for vdW-layered materials.

### Routine Characterization Methods

While we observed significant global and local structural diversity in Sn_*x*_TaSe_2_ by SCXRD and STEM, collected PXRD patterns appear to contain only two sets of peaks, likely matching structures one and two (Figure 3a). The third structure was not identified. Unique peaks matching the orthorhombic structure are absent, potentially made indiscernible by a low signal-to-noise ratio. The weak signal intensity suggests the third structure is a minor phase. Reliance on PXRD alone would under-represent the structural diversity observable with more sensitive techniques, raising the possibility that minor phases in similar systems are being overlooked.

**Figure 3:**
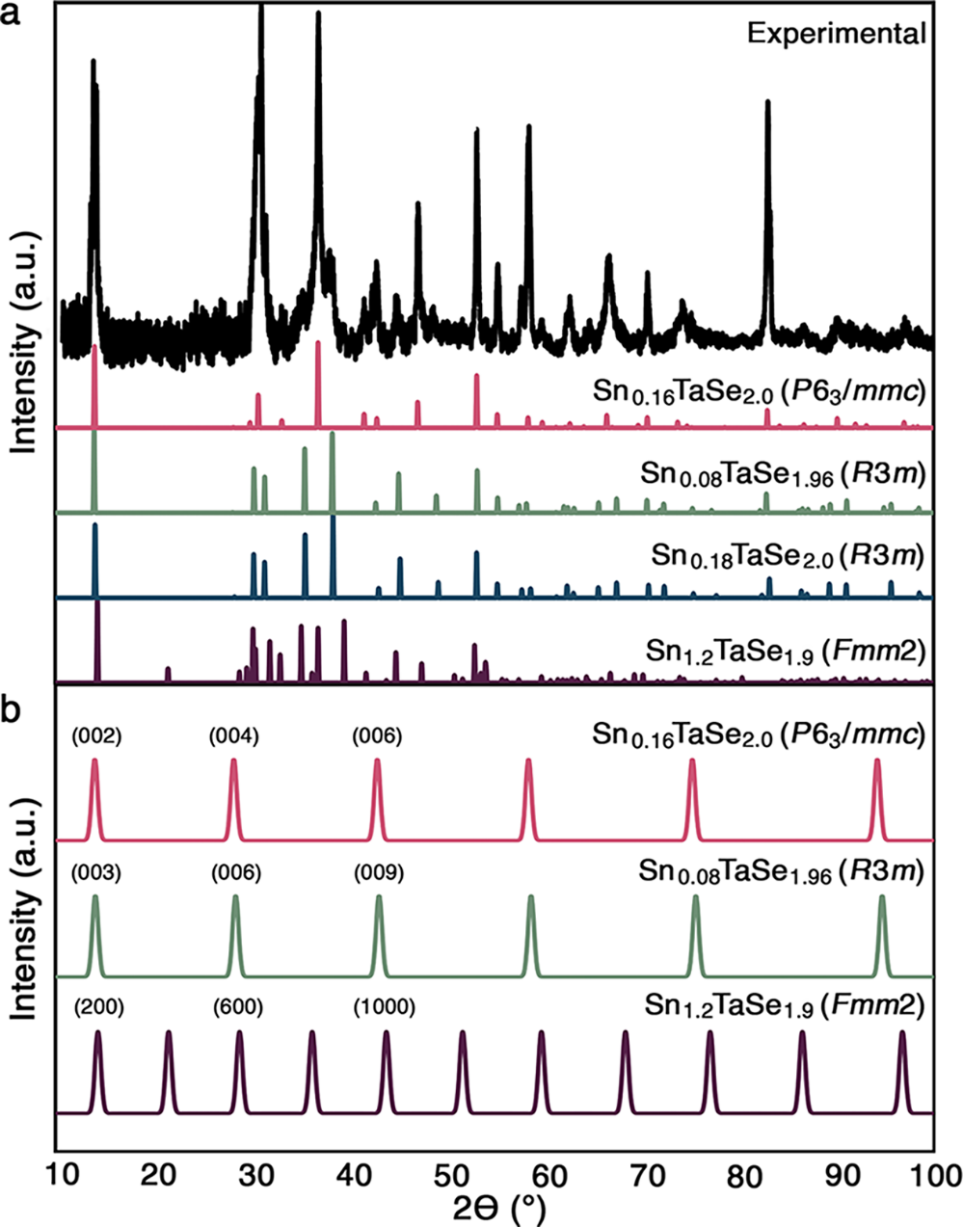
Sn_*x*_TaSe_2_ PXRD patterns. (a) Full experimental and theoretical patterns of the crystal structures observed by SCXRD and (b) (00*l*) (or analogous (*h*00)) peaks for the three Sn_*x*_TaSe_2_ structures with equalized peak intensities.

Phase identification of layered materials like Sn_*x*_TaSe_2_ with PXRD is further complicated because significant changes to structure can result in only subtle changes to PXRD patterns. Stoichiometric changes, like those observed between the *R*3*m* phases in Sn_*x*_TaSe_2_, can produce patterns that vary only by peak intensity, making differentiation challenging. Calculated PXRD patterns for structures with different space groups can also vary by peak intensities alone (Figure S17). Obtaining accurate intensities is challenging as layered structures often have a high degree of preferred orientation (Figure S18). Collecting a partial pattern further diminishes the sensitivity to structural changes. To avoid destructive powdering methods, phase identification of layered materials is often based on collection of (00*l*) peaks of single crystals. The (00*l*) peaks can provide insufficient specificity for structural determination even between structures with significantly different unit cells. Structures one and two have fully overlapping (00*l*) peaks; structure three has peaks that overlap with those of structures one and two but has additional unique (*h*00) peaks (Figure 3b). Matching of (00*l*) peaks would not allow for differentiation between structures one or two, and incomplete collection of peaks for structure three could result in mischaracterization of the phase. Our results demonstrate the limitations of PXRD as a tool for structural determination and verification, and underscore how reliance on this technique could result in overlooked structural diversity.

In the Sn_*x*_TaSe_2_ system we observed significant stoichiometric diversity in addition to the global and local structural diversity. Using EDS, we found a near-continuous range of Sn-intercalation between 0 ≲ *x* ≲ 1. To understand the impact of stoichiometry on properties, we selected 26 single crystals representing the full range of Sn-intercalation for properties measurements (Table S1). Given the significant structural differences observed by SCXRD and the variable stoichiometries measured by EDS, we expected the Raman spectra to vary significantly across the sample set.^13,62–64^ However, the Raman spectra for all measured samples are nearly identical to pure 2*H*-TaSe_2_ for wavenumbers above 80 cm^–1^, representing intralayer modes (Figure 4).^65–67^ Only small shifts in the A_1g_ and E_2g_ peaks were sporadically observed. These findings illustrate the limitations of Raman spectroscopy in verifying the structures of Sn_*x*_TaSe_2_ crystals, highlighting the need for careful multifaceted characterization approaches.

**Figure 4:**
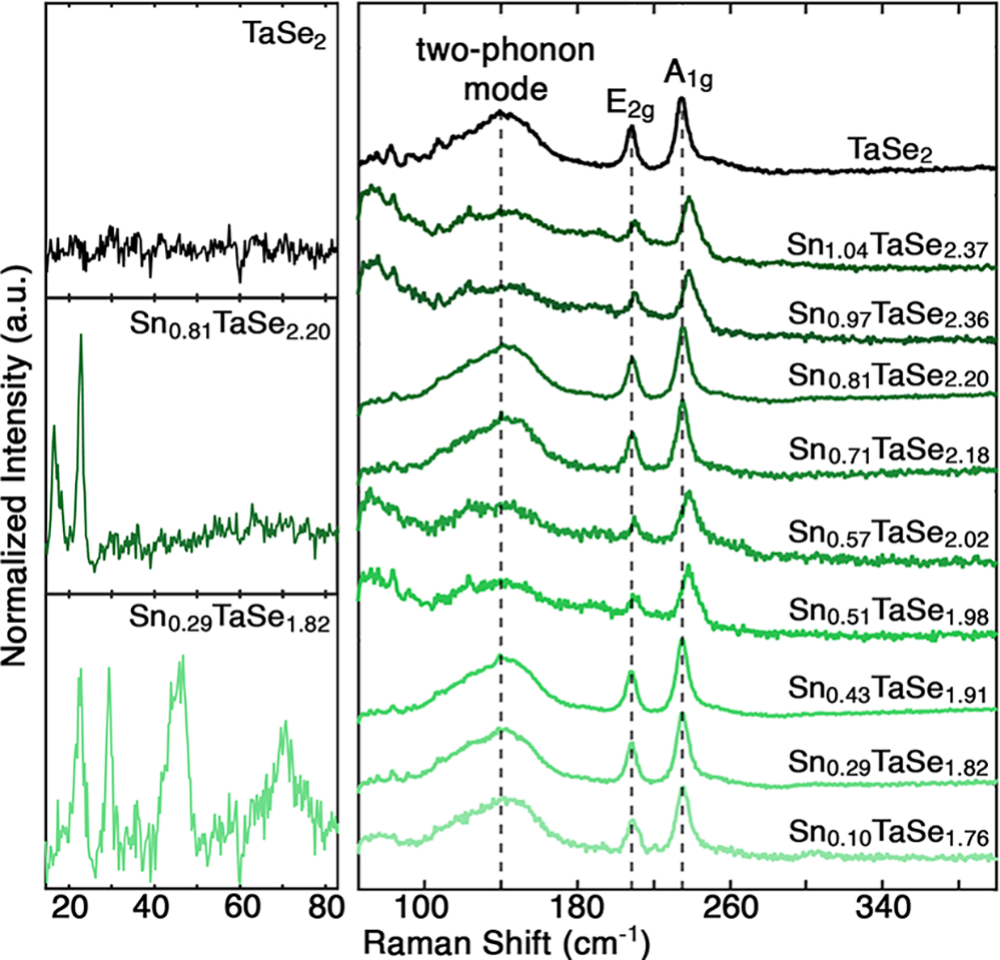
Raman spectra for Sn_*x*_TaSe_2_. Dashed lines indicate the position of the TaSe_2_ peaks. Baseline correction and SiO_2_ subtraction were used for low frequency data; raw spectra are presented in Figures S19–S22.

Low-frequency Raman spectroscopy is known to distinguish between compounds with different symmetries by revealing interlayer breathing and shear modes that are sensitive to TMD stacking orders.^68,69^ Figure 4 shows the low-frequency Raman spectra obtained for Sn_*x*_TaSe_2_ samples representing no (*x* = 0), low (*x* = 0.29), and high (*x* = 0.81) Sn-intercalation. Spectral differences were observed between the three samples, with TaSe_2_ having no observable low-frequency peaks, in agreement with previous ambient experimental results;^67^ Sn_0.81_TaSe_2.20_ having two peaks at 16.5 and 22.8 cm^–1^; and Sn_0.29_TaSe_1.82_ having four peaks at 22.8, 29.4, 45.8, and 70.8 cm^–1^. These spectral differences imply that the compounds are distinct.^70,71^ Some of the visible low-frequency peaks align with calculated inactive modes of 2*H*-TaSe_2_, suggesting intercalation-induced symmetry breaking may have activated these modes.^67,72^ Additional SCXRD and density functional theory phonon-calculations could connect the observed low-frequency modes with the structure to provide an avenue for optical phase verification that higher-frequency Raman spectroscopy cannot provide.

Another common approach to verifying phase composition and structure involves measuring the electronic properties of a phase and matching measured to literature values. Small changes to structure are expected to produce clear changes in electronic behavior.^50,73^ In Figure 5, we summarize our temperature-dependent resistivity (ρ) measurements for Sn_*x*_TaSe_2_ (0.10 ≤ *x* ≤ 1.04). For several crystals, we observe peaks in ρ at ∼111 K, consistent with CDW peaks found in unintercalated TaSe_2_. Despite significant variation in Sn, the *T*_CDW_ does not significantly change across the sample set. The small variations in *T*_CDW_ that are observed appear independent of stoichiometry (Figure S23). The absence of correlation between *T*_CDW_ and stoichiometry is surprising, as *T*_CDW_ typically changes with stacking order (1*T*-TaSe_2_ *T*_CDW_= 473 K (commensurate), 600 K (incommensurate),^74,75^ 2*H*-TaSe_2_ *T*_CDW_ = 90 K (commensurate), 120 K (incommensurate)^76^), and increased intercalation often suppresses CDW behavior.^77,78^

**Figure 5:**
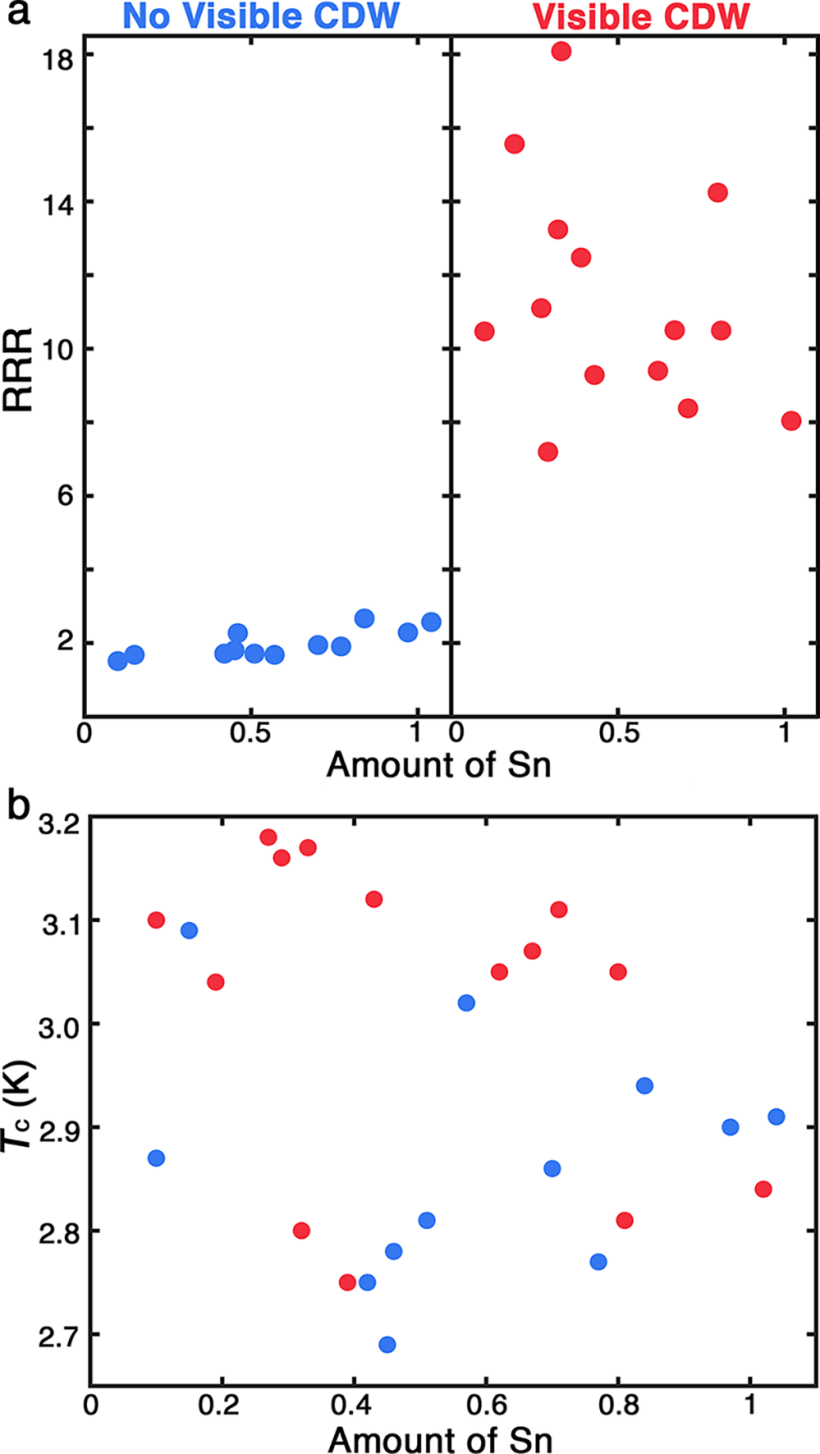
Transport behavior of Sn_*x*_TaSe_2_. (a) RRR plotted against the amount of Sn, partitioned according to the presence or absence of a visible CDW, and (b) *T*_c_ plotted against the Sn amount.

As shown in Figure 5b, *T*_c_ (defined as 0.9ρ_3.3K_) did not significantly vary with changes in stoichiometry. All measured Sn_*x*_TaSe_2_ crystals exhibited superconductivity, with only minor fluctuations in *T*_c_ (2.69 ≤ *T*_c_ ≤ 3.18 K). Measurement of the magnetic susceptibility of a bulk sample corroborates this result (Figure S24). No clear trend or phase groups link *T*_c_ to the amount of Sn or other changes in stoichiometry (Figures S25–S29). Magnetotransport results for Sn_*x*_TaSe_2_ displayed the same lack of correlation between stoichiometry and properties (Figures S30–S33). These results contrast with the observations of Luo et al., who found *T*_c_ to be sensitive to intercalant amount and structure, particularly for the 3*R* phase of TaSe_2–*x*_Te_*x*_.^50^

As multiple phases were observed within a single crystal by STEM, the observed homogeneity in transport measurments could be a result of a single dominant phase or an averaging of all phases. Alternatively, all phases may exhibit comparable electronic behavior. In either case, the diversity of the system would be obscured if electronic properties were used as the metric for structure and composition verification. The observed similarity in *T*_CDW_ and *T*_c_ values could easily be mistaken for evidence of a consistent structure for Sn_*x*_TaSe_2_.

While *T*_c_ does not correlate to changes in Sn_*x*_TaSe_2_ stoichiometry, the measured *T*_c_ values are higher than those measured for pure TaSe_2_ phases. The observed *T*_c_ values represent a dramatic increase over that of 2*H*-TaSe_2_ (*T*_c_ ≈ 0.2 K),^76^ and a slight increase over 3*R*-TaSe_2_ (*T*_c_ ≈ 0.6–3.0 K).^73,79–81^ The substantial change in *T*_c_ for TaSe_2_ from 2*H* to 3*R* was attributed to changes in stacking order by Deng et al.^73^ However, fluctuations in *T*_c_ linked to stacking order do not appear to be present in Sn_*x*_TaSe_2_. This seems to indicate that the origins of *T*_c_ enhancement are not attributable to stacking order alone. Intercalation of Li or Pt into TaSe_2_ has been reported to increase the *T*_c_.^82,83^ The mutual presence of interstitial sites among 3*R*-TaSe_2_, Sn_*x*_TaSe_2_, and other intercalated TaSe_2_ phases may point to involvement of these sites in the enhanced superconducting behavior.

Although electronic properties were insensitive to changes in stoichiometry, we observed a correlation between CDW behavior and crystal quality. Crystal quality, measured by residual-resistivity ratios (RRR), seems to control CDW appearance, with visible peaks present only in crystals with RRR > 7 (Figure 5a). In addition, RRR appears positively correlated to *T*_CDW_ (Figure S34). Correlation of *T*_CDW_ with RRR has been observed in 1*T*-VSe_2_.^84^ Figure 6 presents *T*-dependent ρ curves for samples with and without visible CDWs showing respectively broad or sharp superconducting transitions (for all measured flakes, see Figures S35–S60).

**Figure 6:**
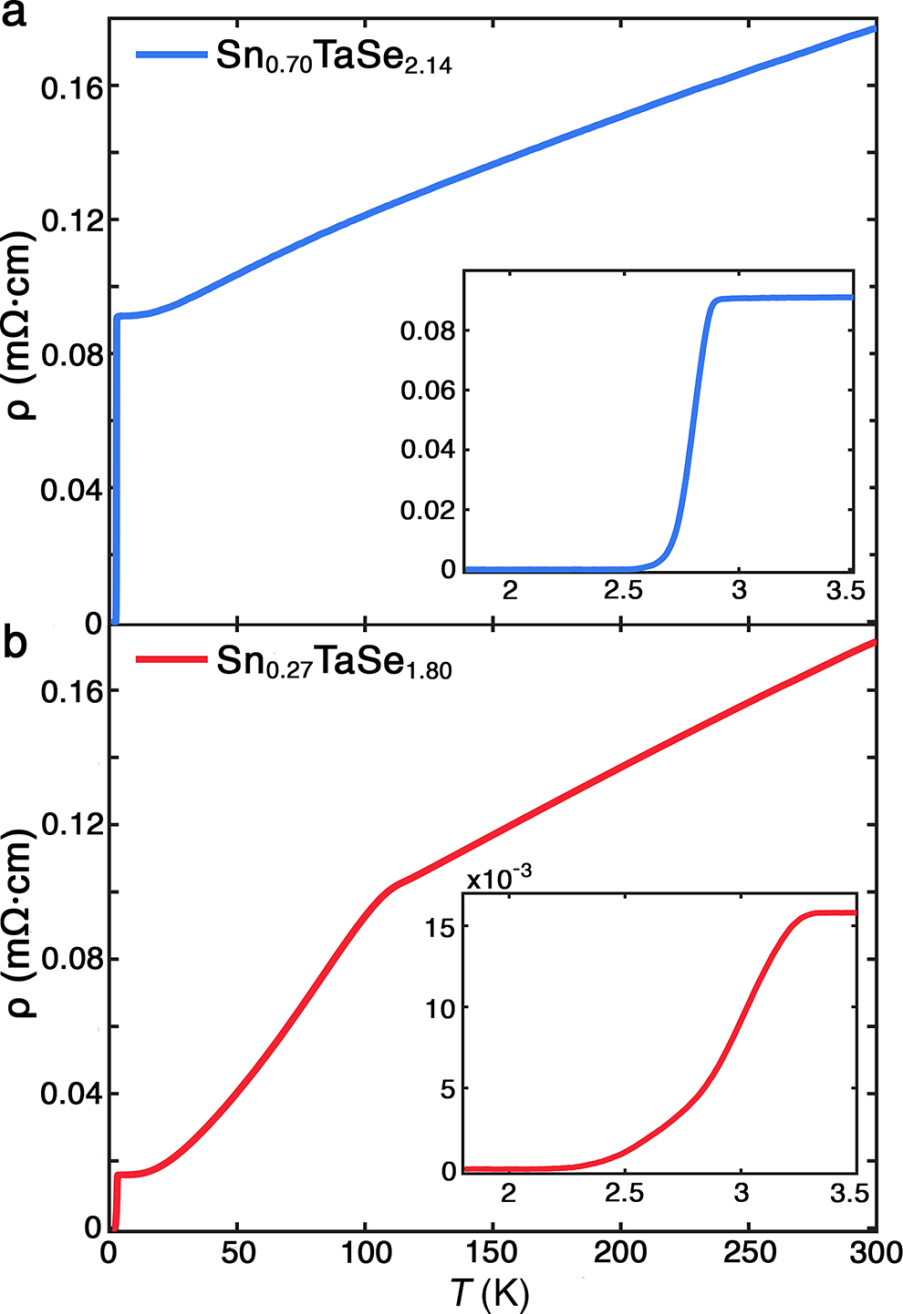
Representative Sn_*x*_TaSe_2_ transport curves. (a) Sn_0.70_TaSe_2.14_ (no visible CDW), inset shows the sharp superconducting transition. (b) Sn_0.27_TaSe_1.80_ (visible CDW), inset shows the broad superconducting transition.

Similar broadening of the superconducting transition appears in CsV_3_Sb_5_ and is assigned to a nearly commensurate CDW state composed of multiple CDW domains.^85^ The domain walls exhibit higher *T*_c_, while the CDW domains exhibit varying degrees of suppressed superconductivity, generating inhomogeneous superconductivity. In Sn_*x*_TaSe_2_ crystals with visible CDW behavior, *T*_c_ does not decrease as expected from the competition between superconducting and CDW phases.^86,87^ The absence of correlated variation may imply the CDW is present in all samples, but that flake quality controls its observability. Overall, the trends in electronic transport for Sn_*x*_TaSe_2_ appear to arise from changes in crystal quality, rather than structure or stoichiometry.

## Conclusions

Our identification of three new Sn_*x*_TaSe_2_ structures, representing a wide range of compositions and crystal symmetries, demonstrates that unexpected structural diversity can emerge from a single TMD growth composition. Complexity in structure and stoichiometry was identified between and within single crystals, illustrating both the global and local structural diversity found in the Sn_*x*_TaSe_2_ system. This heterogeneity was not reflected in PXRD, Raman spectroscopy, or electronic transport data. Materials parameters that were expected to be sensitive to changes in structure, like superconductivity, remained homogeneous across a wide range of stoichiometries. Investigations of intercalated TMDs often rely on assumptions that previously reported structures are correct and comprehensive, synthetic stoichiometries will match the stoichiometries of the products, and properties measurements can unambiguously verify the phase. Our findings suggest that these assumptions may not be valid. Given the intertwined nature of symmetry, topology, and superconductivity for this family of materials, accurate structures are necessary to understand their properties. We anticipate that our work will prompt others to characterize similar materials using multifaceted or high-resolution structural characterization techniques, potentially leading to the discovery of yet more structural diversity and unique physical phenomena.

## Supplementary Material



## Notes

Experimental and instrumentation details, including additional discussion of structures, supplementary figures, and tables (PDF)
