# Supplementary material for: Superconducting Sn-Intercalated TaSe2: Structural Diversity Obscured by Routine Characterization Techniques
Source: J Am Chem Soc. 2025 Oct 21;147(43):39093–102. doi: 10.1021/jacs.5c06808 (PMC12576769; doi:10.1021/jacs.5c06808)
Supplement: Supplementary file 1 [file ja5c06808_si_001.pdf]

Superconducting Sn-Intercalated $TaSe_2$: Structural Diversity Obscured by Routine Characterization Techniques

Brenna C. Bierman,[†] Gillian Nolan,[‡] Hongrui Ma,[¶] Ying Wang,[¶,§,‖] Pinshane Huang,[‡,⊥] and Daniel A. Rhodes[*,‖,§]

†*Department of Chemistry, University of Wisconsin-Madison, Madison, Wisconsin 53706, United States*

‡*The Grainger College of Engineering, Department of Materials Science and Engineering, University of Illinois Urbana-Champaign, Urbana, IL 61801, USA*

¶*Department of Electrical and Computer Engineering, University of Wisconsin-Madison, Madison, WI 53706, United States*

§*Department of Physics, University of Wisconsin-Madison, Madison, WI 53706, United States*

‖*Department of Materials Science and Engineering, University of Wisconsin-Madison, Madison, WI 53706, United States*

⊥*Materials Research Laboratory, University of Illinois Urbana-Champaign, Urbana, IL 61801, USA*

E-mail: darhodes@wisc.edu

Contents

1. Experimental Information and Instrumentation

Crystal Growth of Sn_xTaSe_2 S4

Single-Crystal X-ray Diffraction (SCXRD) S5

Scanning Transmission Electron Microscopy (STEM) S5

Raman Spectroscopy S6

Electronic Transport Measurements S7

Scanning Electron Microscopy (SEM) S7

Powder X-ray Diffraction (PXRD) S8

X-ray Photoelectron Spectroscopy (XPS) S9

Magnetic Property Measurements S9

Density Functional Theory (DFT) Calculations S9

Additional Discussion of Structures S10

2. Supplementary Figures

Figure S1: Example Diffraction of Intercalated TMD "Single" Crystal S16

Figure S2: Example Diffraction of TMD "Single" Crystal S17

Figure S3-6: Precession Images for SCXRD Datasets S18-S21

Figure S7: Tetrahedral Voids between $Se_{180°}$ Layers (Structure Two) S22

Figure S8: XPS for I_2 and $SeCl_4$ grown Sn_xTaSe_2 S23

Figure S9: Example EDS Spectra for Sn_xTaSe_2 S24

Figure S10: EDS Atomic Percentage Map for Sn_xTaSe_2 S25

Figure S11-S16: Additional STEM Images and EDS Results S26-S31

Figure S17: Theoretical PXRD Patterns with Effectively Identical Peak Positions S32

Figure S18: Experimental PXRD Pattern for Sn_xTaSe_2 with Preferred Orientation S33

Figure S19-22: Raw Low-Frequency Raman Spectra S34-S37

Figure S23: T_{CDW} Plotted Against the Amount of Sn S38

Figure S24: Low-Temperature Magnetic Moment Results for Sn_xTaSe_2 S39

Figure S25-29: Correlations between T_c and Stoichiometry S40-S44

Figure S30-33: Magnetotransport Results for Sn_xTaSe_2 S45-S46

Figure S34: Correlation between T_{CDW} and RRR S47

Figure S35-S60: Transport Curves for Sn_xTaSe_2 Samples S48-S60

Figure S61: Typical Products of CVT Sn_xTaSe_2 Growth S61

Figure S62-S63: Products of Sn-Flux Growth of Sn_xTaSe_2 S62-S63

Figure S64: Example of Sample Preparation for Transport Measurments S64

Figure S65-S66: Example T_{CDW} Calculations S65-S66

Figure S67: Critical Current Density for Sn_xTaSe_2 S67

Figure S68-S70: EBSD Maps and Kikuchi Patterns for Sn_xTaSe_2 S68-S70

Figure S71-S72: Band Structures for Structures One and Two S71-S72

3. Supplementary Tables

Table S1: Stoichiometry, Transition Temperatures, and RRR for Measured Flakes S73

Table S2-S3: Crystallographic Parameters for Dataset 1 ($Sn_{0.18}TaSe_{2.0}$) S74

Table S4-S5: Crystallographic Parameters for Dataset 2 ($Sn_{0.08}TaSe_{1.96}$) S75

Table S6-S7: Crystallographic Parameters for Dataset 3 ($Sn_{0.16}TaSe_{2.0}$) S76

Table S8-S9: Crystallographic Parameters for Dataset 4 ($Sn_{1.2}TaSe_{1.9}$) S77

Table S10-S11: Phase Data and Atomic Parameters for $Sn_{0.36}TaSe_{2.11}$ (Theoretical) S78

Table S12-S13: Phase Data and Atomic Parameters for $Sn_{0.17}TaSe_2$ (Theoretical) S79

Table S14: DFT Total Energy Calculations S80

Table S15-20: Atomic and Cell Parameters for DFT Calculations S81-S86

Experimental Information and Instrumentation

Crystal Growth of Sn_xTaSe_2

Sn-intercalated $TaSe_2$ was grown via chemical vapor transport (CVT). Sn shot (Alfa Aesar, 99.9999+%), Ta powder (Alfa Aesar, 99.98%), and Se shot (Alfa Aesar, 99.999+%) were added in a molar ratio of 1:1:2 (Sn:Ta:Se) directly to a fused quartz tube along with the transport agent, I_2 (Alfa Aesar, 99.999%) or $SeCl_4$ (Thermo Fisher Scientific, 99.5%). The quartz tube was then sealed under vacuum (∼1 mTorr). The initial synthesis utilized CVT in a two-zone furnace set to 1000/900 °C for 14 days, followed by cooling to 500 °C at 5 °C/hr. This produced the intercalated phase in a powdered, polycrystalline form. Increasing the temperature of the furnace to 1150/1000 °C produced large flakes (mm size), even when left at the maximum temperature for a shorter period (5 days). Typical products of the high temperature synthesis included: Sn_xTaSe_2 flakes, polycrystalline Sn_xTaSe_2, and unreacted Sn (Figure S61).

Changing the following synthetic conditions yielded flakes with no obvious differences in stoichiometry or appearance: utilizing a horizontal single zone tube furnace with the same profile (max temperature of 1150 °C), cooling at a rate of 5.4 °C/hr, using a temperature gradient of 1150/1050 °C rather than 1150/1000 °C, increasing the time at maximum temperature (up to 20 days), and cooling to 550°C rather than to 500°C. Pre-reacting the elements in the absence of a transport agent before subsequent addition of the transport agent did not appear to have a significant impact on the quality or size of crystals, nor did adding excess Se (5% or 10% excess). Products of the $SeCl_4$ reactions did not have obvious quantitative differences, but qualitatively produced thicker crystals (along the c direction) and more crystals without Sn (as determined by energy dispersive X-ray spectrometry).

Synthesis via Sn-flux was also attempted. Samples with a 10:1:2 (Sn:Ta:Se) molar ratio were prepared by combining elements in an alumina crucible capped with an alumina filter and a second crucible inside quartz tube, then sealing under vacuum (∼1 mTorr). Samples

were placed in a box furnace at 1000 °C for 48 hours before cooling at 4 °C/hour to 500 or 400 °C. At the target temperature, the samples were removed from the furnace and immediately inverted and centrifuged. Products were polycrystalline Sn_xTaSe_2 (Figure S62) and SnSe (Figure S63). Samples prepared with the flux method were not used in this work.

Single-Crystal X-ray Diffraction

Silver, plate-like crystals were picked from the samples, cut with a scalpel, and lifted onto a MiTeGen MicroMount in oil. Single-crystal evaluation and data collection was performed at room temperature on a Bruker Quazar SMART APEXII diffractometer with Mo Kα (λ = 0.71073 Å) radiation at 50 kV and 0.6 mA. Integration and scaling was done in APEX3 or APEX6. Structures were solved by charge flipping as implemented by the Superflip program in Jana2006 (dataset one) or by intrinsic phasing in ShelXT in Olex2 (datasets two through four).[1–4] Final refinement for all structures was done in Olex2 using L.S. instruction in ShelXL.[5] Additional atomic parameter information is provided in tables S2-S9. Discussion of structural solutions and refinement is included in "Additional Discussion of Structures" (pages S10-S15). Crystal structure data has been deposited at the Cambridge Crystallographic Data Centre (CCDC) under reference numbers 2441345 ($Sn_{0.18}TaSe_{2.0}$), 2441346 ($Sn_{0.08}TaSe_{1.96}$), 2441347 ($Sn_{1.2}TaSe_{1.9}$), and 2441348 ($Sn_{0.16}TaSe_{2.0}$). This data can be obtained free of charge from the website (https://www.ccdc.cam.ac.uk/structures/). Structure figures were made with VESTA.[6]

Scanning Transmission Electron Microscopy

Crystallographic orientation of scanning transmission electron microscopy (STEM) samples was determined using electron backscatter diffraction (EBSD) (see scanning electron microscopy (SEM) section for more details, pages S7-S8). Samples were coated with 5-10 nm of protective amorphous carbon via thermal evaporation and prepared according to standard focused ion beam (FIB) lift-out procedures for TEM lamellas in a Thermo Fisher Scientific

Helios 600i DualBeam FIB-SEM and and Thermo Fisher Scientific Scios 2 DualBEAM FIB-SEM. A cryo can was used during thinning.

STEM and energy-dispersive X-ray spectroscopy (EDS) data were acquired in a Thermo Fisher Scientific Themis Z aberration-corrected STEM operated at 300 kV with a convergence angle of 25.2 mrad. Annular dark-field (ADF) STEM images were taken with a collection angle of 64-200 mrad and EDS data was collected on a Super-X EDS detector. The ADF-STEM images in Figures 2a,b and S11 were taken as a series with drift corrected frame integration (DCFI) as implemented in Thermo Scientific's Velox Software.

Raman Spectroscopy

Samples were exfoliated onto SiO_2 before collection. Bulk samples were used for collection, rather than few-layer samples. Standard Raman spectra were collected using a Horiba LABRAM HR Evolution Raman spectrometer with a 532 nm excitation laser and 1800 gr/mm at room temperature. When long collection windows were employed or high filter values (either 1% or 3.2% depending on the sample), burning of the sample surface occurred, distorting the spectra. Low-frequency Raman spectra were collected using an Andor Shamrock 500 spectrometer with 1800 gr/mm and an air-cooled CCD camera at room temperature. The 633 nm excitation laser was used with one customized OptiGrate band pass filter (BPF) and three customized Optigrate band notch filters (BNF) to increase the purity and to enable the low-frequency Raman measurement down to 10 cm^{-1}. A background correction and SiO_2 subtraction was used on the low-frequency data presented in the main text. Raw spectra for SiO_2, $TaSe_2$, $Sn_{0.81}TaSe_{2.20}$, and $Sn_{0.29}TaSe_{1.82}$ are presented in Figures S19-S22. Some differences in low-frequency peak intensities were observed between different exfoliated flakes within a sample, likely due to differences in sample thickness or orientation.

Electronic Transport

Temperature dependent electrical resistivity data was collected using a Quantum Design Dynacool physical property measurement system (PPMS). Single crystals were mounted on SiO_2 and 0.025 mm gold wires (99.95%, Thermo Fischer Scientific) were attached with 4929N silver paste in a standard four point probe arrangement (Figure S64). The sample was placed on a Quantum Design Resistivity Sample Puck, with a thin layer of Apiezon N grease between the puck and SiO_2. The residual-resistance ratio (RRR) was calculated by dividing the resistance at 300 K by the resistance just before the superconducting transition (3.3 K). T_{CDW} calculation was performed in MATLAB by first subtracting a linear fit from the resistance data. The derivative of the linear-subtracted resistance data was then taken. The onset temperature was the point where this derivative equaled zero. Figures S65-S66 demonstrate these plots for samples with and without visible CDW.

Magnetoresistance data was collected on a Cryo Industries Cryostat equipped with a variable temperature insert and a 14 T magnet. Measurements used a Stanford Research 500 kHz DSP Lock-In Amplifier with a Keithley 6221 DC and AC current source (set to 1.5 mA current for magnetotransport measurements). Single crystals were mounted on SiO_2 and gold wires were attached with 4929N silver paste in a standard six point Hall bar arrangement. For critical current measurements, a constant 1 nA current (10 mV with a 1 MΩ resistor) was supplied from the Lock-In Amplifier while the current was changed on the current source. Data was smoothed with a moving average. Current dependence data was also collected and fit with the Matlab program written by Talantsev et al. (Figure S67).[7]

Scanning Electron Microscopy

Energy-dispersive X-ray spectra were collected on a Zeiss Gemini 450 field emission scanning electron microscope (SEM) equipped with a Thermo Noran energy dispersive X-ray microanalysis system. An accelerating voltage of 20 or 21 kV was used with sufficient probe current to produce a dead time between 35% and 60%. Samples were mounted on SiO_2 with silver paste

on the ends of each flake, then the SiO_2 was secured on carbon tape. Stoichiometries were determined by collecting many points across each flake, then averaging to generate the final reported stoichiometries. Example data and an elemental map are shown in Figures S9-S10. The EDS data consistently found lower Se:Ta ratios in structures with low Sn-intercalation, and higher Se:Ta ratios in those with high Sn-intercalation.

Electron backscatter diffraction (EBSD) data was collected on a Zeiss Gemini 300 field emission SEM equipped with a Oxford Aztec EBSD system. Samples were loaded on a 70° tilted sample holder and tilt correction was enabled. EBSD was primarily used to determine crystallographic orientation for preparation of STEM samples. We found that the same Kikuchi pattern can appear to match either structure one or two, and maybe structure three (Figure S68). The Kikuchi pattern itself does not appear to change between the different map points. This result suggest that EBSD may not provide the specificity needed to distinguish between the different structures observed by SCXRD. We also observed regions that appeared to match a single structure (Figures S69-70). These two regions were used for preparation of STEM samples. These result suggest that EBSD may be able to provide the specificity needed to distinguish between the different structures observed by SCXRD. More investigation into the sensitivity of EBSD to Sn_xTaSe_2 structure would be needed resolve the ambiguity in results.

Powder X-ray Diffraction

Powder X-ray diffraction patterns were collected on a Bruker D8 Advance Powder X-ray diffractometer with Cu Kα radiation (λ = 1.5418 Å), at 50 kV voltage and 1,000 uA current over a 2θ range of 10 to 100°. Powder samples were prepared using a ceramic mortar and pestle with isopropyl alcohol.

X-ray Photoelectron Spectroscopy

X-ray photoelectron spectroscopy (XPS) was performed on powdered samples with a Thermo Scientific Kα X-ray photoelectron spectrometer equipped with a monochromatic Al Kα X-ray source with a spot size of 400 μm and the electron flood gun on. The X-ray gun was operated at 15 kV and 20 mA. Survey scans were collected from 10 to 1300 eV with step size of 1 eV and pass energy of 200 eV. Elemental scans were collected for Sn (479 to 500 eV, step size of 0.1 eV, and pass energy of 50 eV) Ta (20 to 30 eV, step size of 0.1 eV, and pass energy of 50 eV), and Se (154 to 169 eV, step size of 0.1 eV, and pass energy of 50 eV). Peak fitting was done using Thermo Scientific Avantage software.

Magnetic Property Measurements

Magnetic moment measurements were preformed on a magnetic property measurements system (MPMS) 3 Quantum Design Superconducting QUantum Interference Device (SQUID) magnetometer with a magnetic field of 0.1 Tesla. A plastic straw was used as the sample holder. The sample (121.1 mg) was placed in a gelatin capsule and held in place with Elmer's rubber cement.

Density Functional Theory Calculations

Band structures were calculated with Quantum ESPRESSO[8,9] using Projector Augmented-Wave (PAW)[10] method with Perdew-Burke-Ernzerhof for solids (PBESol) exchange-correlation (XC) energy functional in the generalized gradient approximation[11] obtained from PseudoDojo.[12] Brillouin zone paths were determined based on space group and atomic parameters.[13] Band structures for intercalated and un-intercalated versions of structures one and two are provided in Figure S71-S72. Atomic optimization was done with ABINIT[14,15] using norm-conserving Hartwigsen–Goedecker–Hutter (HGH) pseudopotentials with local density approximation (LDA) XC energy functional.[16] Optimization was done in two steps: first

relaxing the atomic positions, then relaxing the cell and atomic positions together. A tolmxf of 5.0000E-05 Ha/Bohr was used. Calculated energies for all three structures for each step of optimization are provided in Table S14. As all structures have partially occupied sites, structures were approximated with sites close to observed stoichiometry. The atomic and cell parameters for the direct and optimized structures are provided in Tables S15-S20.

Additional Discussion of Structures

Before discussing each structure individually, it is important to acknowledge some of the limitations of single-crystal X-ray diffraction for TMDs. Atomic identity assignment primarily relies on the electron density at a specific site. Confidently identifying the elemental identity of partially occupied sites can therefore be challenging. Assignment is particularly difficult when the electron density at a site is small enough to allow any of the possible atom types to feasibly occupy the position. These types of partially occupied sites are prevalent in intercalated TMDs. Pairing SCXRD with elemental analysis techniques can provide some guiding information, but the observed experimental stoichiometry may not be in perfect agreement. Another challenge with structure determination by X-ray diffraction is space group assignment. Comparing precession images and reflection conditions, or using programs like XPREP[17] or Zürich Space Group Helper (ZSGH)[18], can offer guidance on possible groups but generally cannot provide an unambiguous answer. Often multiple space groups can provide high quality models for a given set of reflections. The goal of the crystallographer is to pick an appropriate model that captures all the symmetry elements present. Even with these challenges, SCXRD is arguably the most powerful method for three-dimensional structural determination for solids.[19]

Structure One

The first dataset for structure one, $Sn_{0.18}TaSe_{2.0}$, refined well in two different space groups: $R3m$ or $R\bar{3}$. The $R3m$ model was chosen for the final model, with an inversion twin refinement.

If the inversion twin is not modeled, the $R3m$ model produces a Flack parameter (x) of 0.48(17) and a Hooft parameter (y) of 0.49(5). An x or y value of 0.5 may indicate the presence of an equal twin fraction, particularly an inversion twin.[20] Refining the $R3m$ model with a 49(17)% inversion twin produced a y value of -0.01(4). An x or y value of 0.5 can also indicate an omitted inversion center, suggesting a centrosymmetric space group.[21,22] Assignment to an inversion center leads to the $R\bar{3}$ model. This model refines well (R_1=.0379, wR_2=.0847, R_{int}=.0391, Max/Min Peak=2.5/-6.2, Goof=1.295) but has a large residual electron density hole and more disorder than the $R3m$ model. Particularly, the $R\bar{3}$ model features rotational disorder of the Se environment around Ta, similar to structure two (forming octahedra rather than trigonal prisms). An x or y values of 0.5 does not always indicate a twin fraction or an inversion center.[23] In reciprocal space, a possible non-merohedral twin lattice is visible, representing a 180° rotation around [-1 1 0]: (0.99 -0.14 -0.06 / 0.14 0.99 -0.02 / 0.06 0.01 1). The reflections were too weak to be reliably processed, so were ignored.

The second dataset for structure one, $Sn_{0.08}TaSe_{1.96}$, was refined with a merohedral twin component: ($\bar{1}$ 0 0 / 1 1 0 / 0 0 $\bar{1}$). The two domains are then related by a -180° rotation around c*. The refinement indicates a 25.2(7)% contribution from the twin component. Without the twin component modeled, electron density peaks appear that resemble positional disorder. In the model without the twin law, the overall stoichiometry is $Sn_{0.11}TaSe_{1.63}$. Both Se sites are deficient (85% and 78%), Ta is disordered across three sites (81.7%, 9.4%, and 8.9%), and Sn is disordered across two low occupancy sites (7% and 3%). All partial sites have a stable anisotropic refinement. With the twin law modeled, the disordered sites disappear, and the Se sites refine to near full occupation.

Structure Two

Structure two has some significant uncertainties. The final model has both rotational and positional disorder. For structure one, both of these traits only appeared in incomplete models. However, after extensive attempts at modeling with different unit cells, space groups,

and twin laws, the disorder observed in structure two could not be eliminated.

In dataset one, changing the model from a centrosymmetric space group ($R\bar{3}$) to a non-centrosymmetric space group ($R3m$) eliminated the apparent rotational disorder. With dataset three, selection of a non-centrosymmetric space group did not remove the rotational disorder. The rotational disorder appears as a second Se site. In centrosymmetric space groups, the two Se sites are each ∼50% occupied. When a non-centrosymmetric space group is selected, both sites are ∼100% occupied. This is unreasonable as the distance between these sites is only 1.98 Å. Fully occupied sites with unreasonable distances can be indicative of an incorrect unit cell. In this case, there are two reasonable super cells, doubling along c (a = b = 3.44, c = 25.24) or doubling a and b (a = b = 5.96, c = 12.62). Supercell reflections were not apparent for either of these cells in reciprocal space. For completeness, both cells were tested in all allowable space groups (as determined by the precession images and reflection conditions). No reasonable, stable model was produced. The a = b = 5.96, c = 12.62 cell appeared to have a reflection pattern in reciprocal space that resembled a second order reticular twin, but selection of these cells only produced low quality models (absorption correction for twin domains was done using TWINABS).[24] Lower symmetry unit cells were also modeled, primarily a C-centered orthorhombic cell (a = 3.44, b = 5.96, and c = 12.63) and a C-centered monoclinic cell (a = 3.44, b = 5.96, c = 12.63, β = 105.25). Models with these cells resulted in ADDSYM detecting missing symmetry elements.[25–27] Such a warning can sometimes be the result of pseudo-symmetry caused by pseudo-merohedral twinning.[28] Attempts to model any such twin domain were unsuccessful. The a = b = 3.44, c = 12.62 primitive hexagonal/trigonal unit cell, with a centrosymmetric space group then seemed most promising. Only two space groups fulfill both those traits and the observed reflection conditions: $P6_3/mmc$ or $P\bar{3}1c$. If $P\bar{3}1c$ is selected, ADDSYM detects missing symmetry elements, recommending $P6_3/mmc$.

The final $P6_3/mmc$ model appears to have positionally disordered Ta and Se. In dataset two, the positional disorder could be accounted for with a merohedral twin law. No reasonable

merohedral or pseudo-merohedral twin law could be found for dataset three. The Ta2 site sits on an electron peak of 23 e/Å^{-3}. The Ta2 site could be attributed to an in-layer stacking fault, like those reported by Luo et al.[29] The Se3 site sits on a peak of 10.3 e/Å^{-3}. This site appears to be a coordinating Se site for Ta2. The apparent coordination is supported by similar occupancies on the two sites (11(14)% for Ta2 and 13(10)% for Se3). The Se3 site could be modeled as Ta instead. The maximum residual electron density peak in the final model is located between Ta1 and Se3. This could suggest a continuous disorder from the center to the face of the prism. This type of disorder could be attributed to vertical stacking faults, like those observed by transmission electron microscopy.[30–32] The assignment of Se to the site is chosen as 1) in-layer stacking faults seem more common than vertical stacking faults, 2) the refinement values are better with Se at the site as compared to Ta (R_1=.0495, wR_2=.1438, R_{int}=.0734, Max/Min Peak=4.63/-3.66, Goof=1.521), and 3) the electron density peak was not stable when modeled, and its appearance may be due to diffuse scattering.

The crystal selected for the collection of dataset three was grown using $SeCl_4$ as the transport agent; the other three crystals used for collection were synthesized using I_2. A dataset was collected for another $SeCl_4$ grown crystal that also appears to solve well in generally the same structure (varying slightly in the position of the partial Ta sites). This dataset has a lower quality refinement (R_1=.0836, wR_2=.1802, R_{int}=.0803, Max/Min Peak=7.0/-8.3, Goof=1.249), but supports the reproducibility of the observed phase. Structure two does not seem to be exclusive to $SeCl_4$ grown crystals, as the PXRD pattern shown in Figure 2 was obtained from an I_2 grown sample, and appears to contain peaks matching structure two. Although, as discussed in the main text, this is not conclusive evidence of structure two. SCXRD, or other high-resolution techniques, would be required for unambiguous assignment.

Structure Three

The dataset for structure three had the most significant twin domains that could not be modeled. Reflections from twin domains are visible in the precession images, particularity in the (0kl) plane. In reciprocal space, a possible non-merohedral lattice is visible, representing a 180° rotation around c*: (0.99 0.10 -0.06 / -0.10 1 0.01 / 0.06 -0.01 1). The reflections were too weak to be reliably processed, so this possible twin was ignored.

The dataset had two likely unit cells, the face-centered orthorhombic cell (as reported), or a C-centered monoclinic cell (a=3.42, b=6.023, c=12.47, β=97.86°). The observed reflections for both show a strange lack of systematic absences from symmetry elements. The monoclinic cell has possible space groups of $C2$, Cm, or $C2/m$. Only models in $C2$ seem to produce a reasonable solution, but in these models, ADDSYM detects missing symmetry, suggesting $Fmm2$. Attempts to model a twin domain did not resolve this message from ADDSYM.

The final $Fmm2$ model appears to have positionally disordered Ta. The Ta2 site sits on an electron peak of 10.1 e/Å^{-3}. This site is likely due to stacking disorder; similar disorder has been reported in $Ba_{0.75}ClTaSe_2$.[33] The positional disorder disappears with a BASF/TWIN refinement of 0.30(4)/(1 0 -0.06 0 1 0.01 -0.06 0.01 1). This twin law was not used though, as the twin law is seemingly nonsensical. ShelXL can only process reflections that overlap, as those would be the only reflections contained in the .hkl file. This twin law would not match the reflections of the first cell, and thus could not be refined by ShelXL alone. Processing as a separate twin domain did not eliminate the electron peak assigned to Ta2.

The Sn sites and the Ta2 site could not be refined anisotropically. When the occupancy of Ta2 is refined (under the condition that both Ta sites sum to a total occupancy of one), the occupancy goes to just under 0.05, which makes the parameters non-positive definite. Thus, the occupancy was fixed at 0.05, where the occupancy is stable. This instability could be caused by the applied occupancy restriction. The Sn sites also have fixed occupancies. When the occupancies are refined, the thermal ellipsoids balloon to an unreasonable degree. If refined anisotropically, the ellipsoids elongate. Potentially, additional Sn sites surround

the modeled sites, causing the instability in the model. This unmodeled disorder could also contribute to the poor refinement values.

The crystal used for structure three was cut from the $Sn_{1.02}TaSe_{2.25}$ sample. The stoichiometry obtained from the final refinement ($Sn_{1.2}TaSe_{1.9}$) is generally in line with this EDS result as some difference is expected due to inhomogeneity across the flake. This particular flake had charge density wave behavior observable in the transport data.

Supplementary Figures

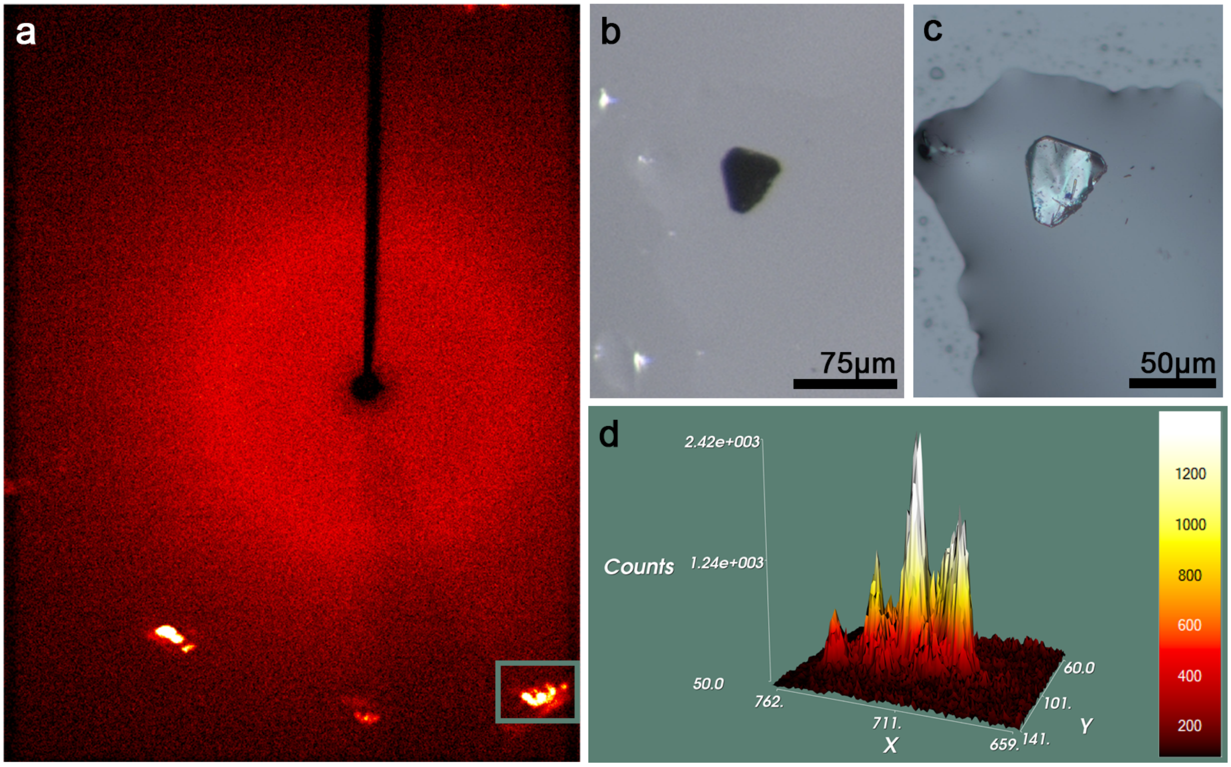

Supplementary Figure 1: Example diffraction of Sn_xTaSe_2 "single" crystal. (a) diffraction image of Sn_xTaSe_2 crystal showing peak smearing. (b) stereo microscope image of corresponding Sn_xTaSe_2 crystal. (c) optical microscope image of corresponding Sn_xTaSe_2 crystal. (d) 3D view of peaks in boxed area of diffraction image (a).

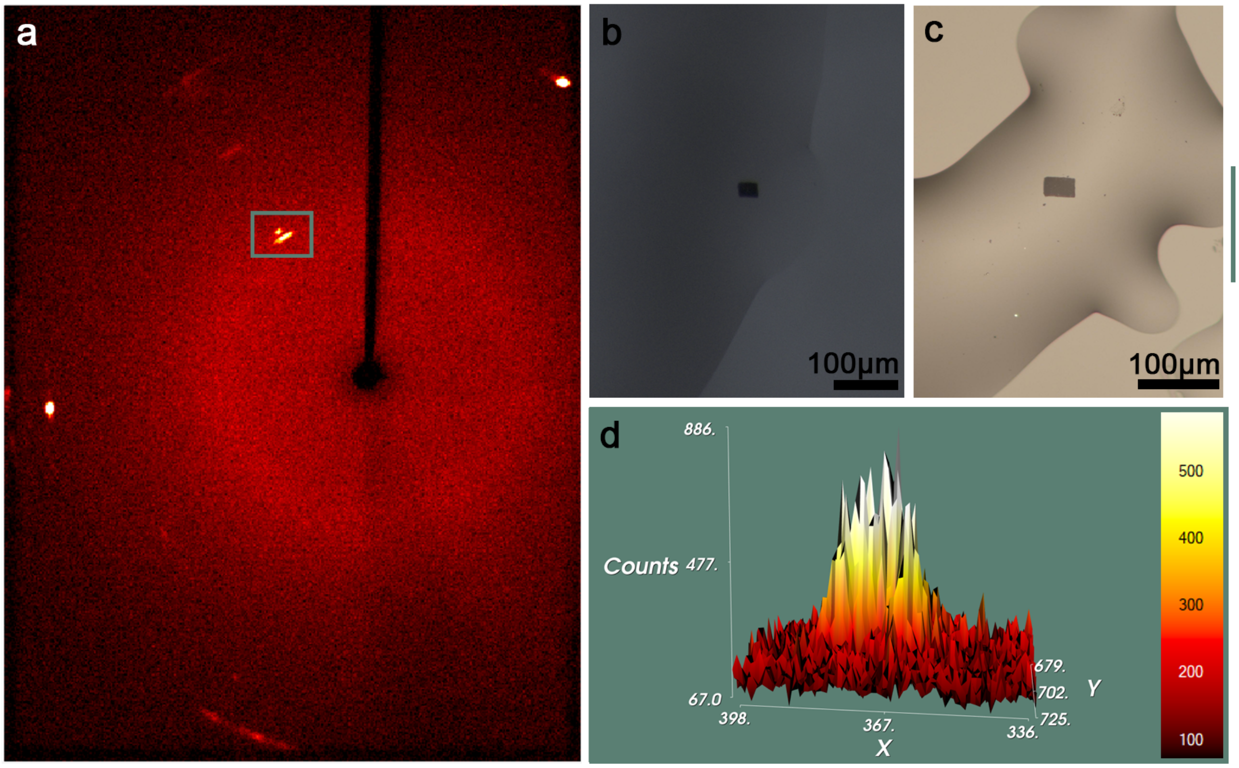

Supplementary Figure 2: Example diffraction of $2M$-WSe_2 "single" crystal. (a) diffraction image of $2M$-WSe_2 crystal showing peak smearing. (b) stereo microscope image of corresponding $2M$-WSe_2 crystal. (c) optical microscope image of corresponding $2M$-WSe_2 crystal. (d) 3D view of peaks in boxed area of diffraction image (a).

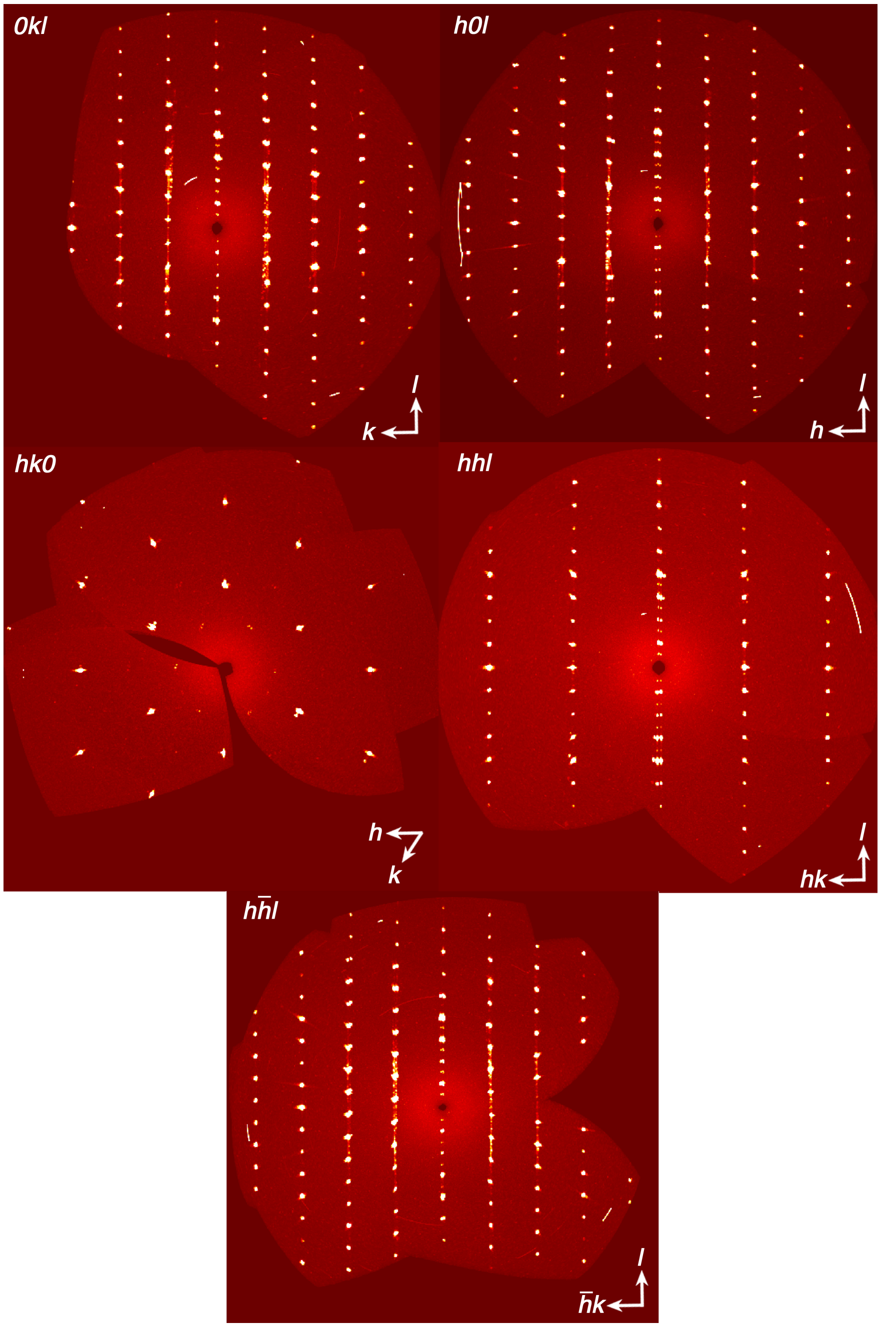

Supplementary Figure 3: Precession images for structure one, dataset one (hexagonal axes).

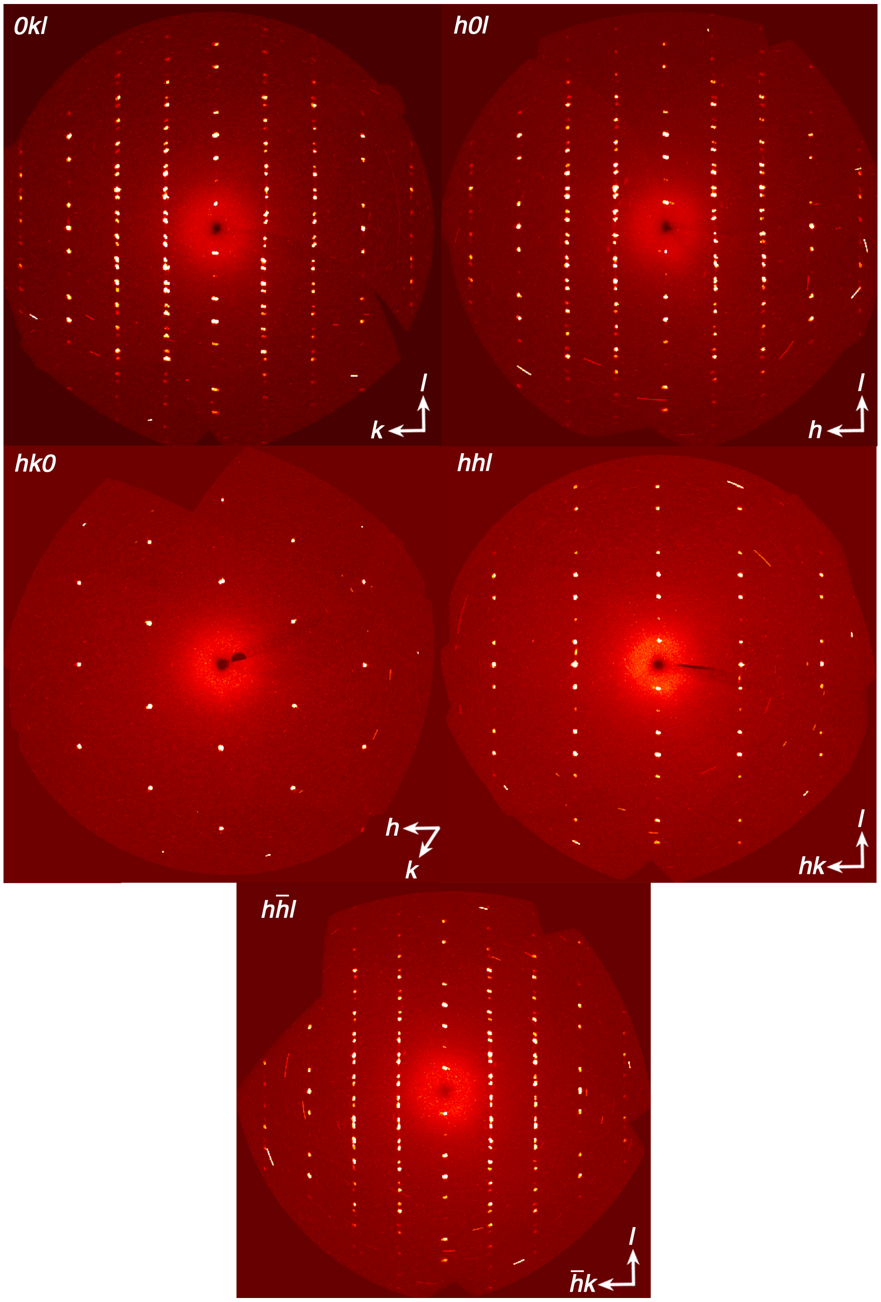

Supplementary Figure 4: Precession images for structure one, dataset two (hexagonal axes).

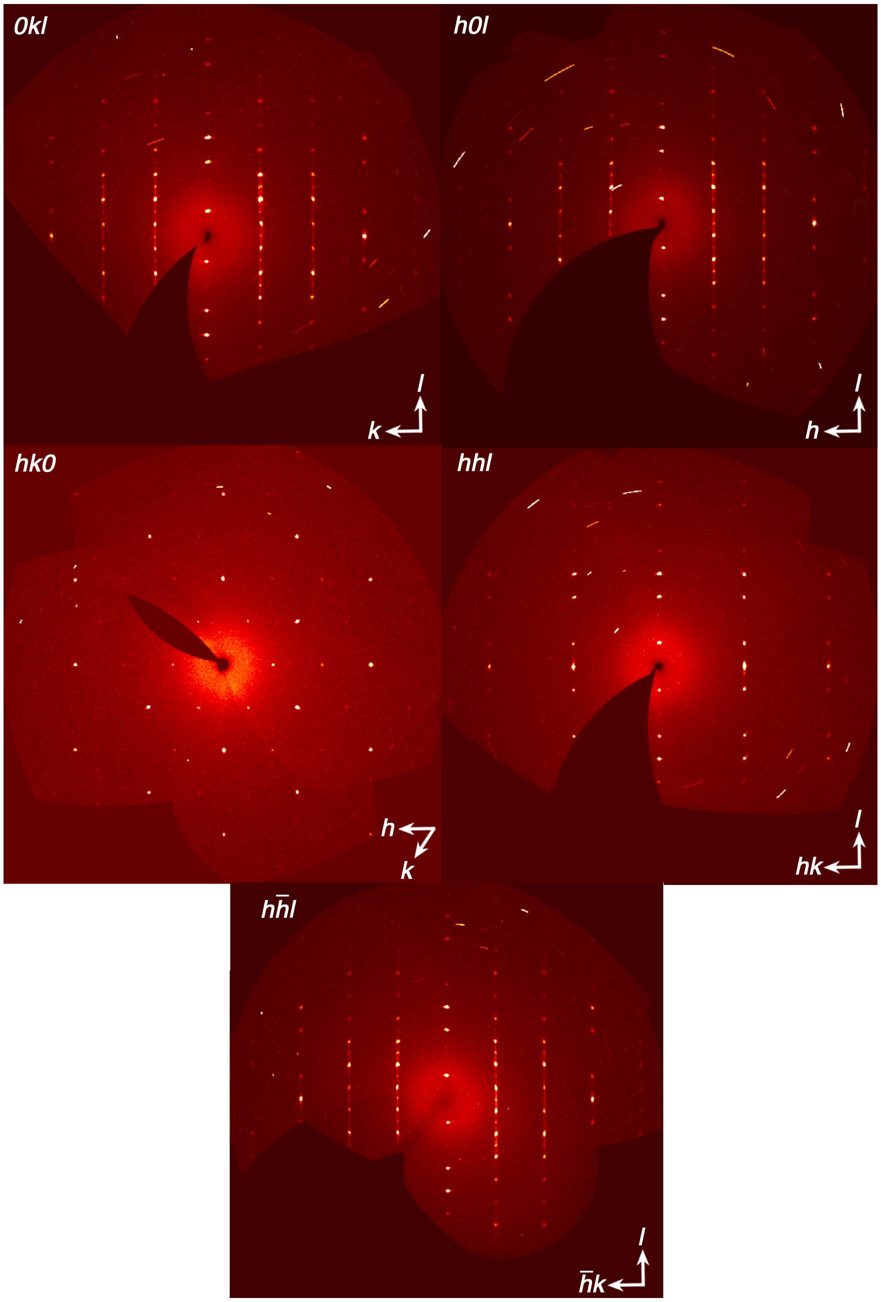

Supplementary Figure 5: Precession images for structure two. Smearing along l is visible, possibly from stacking faults.

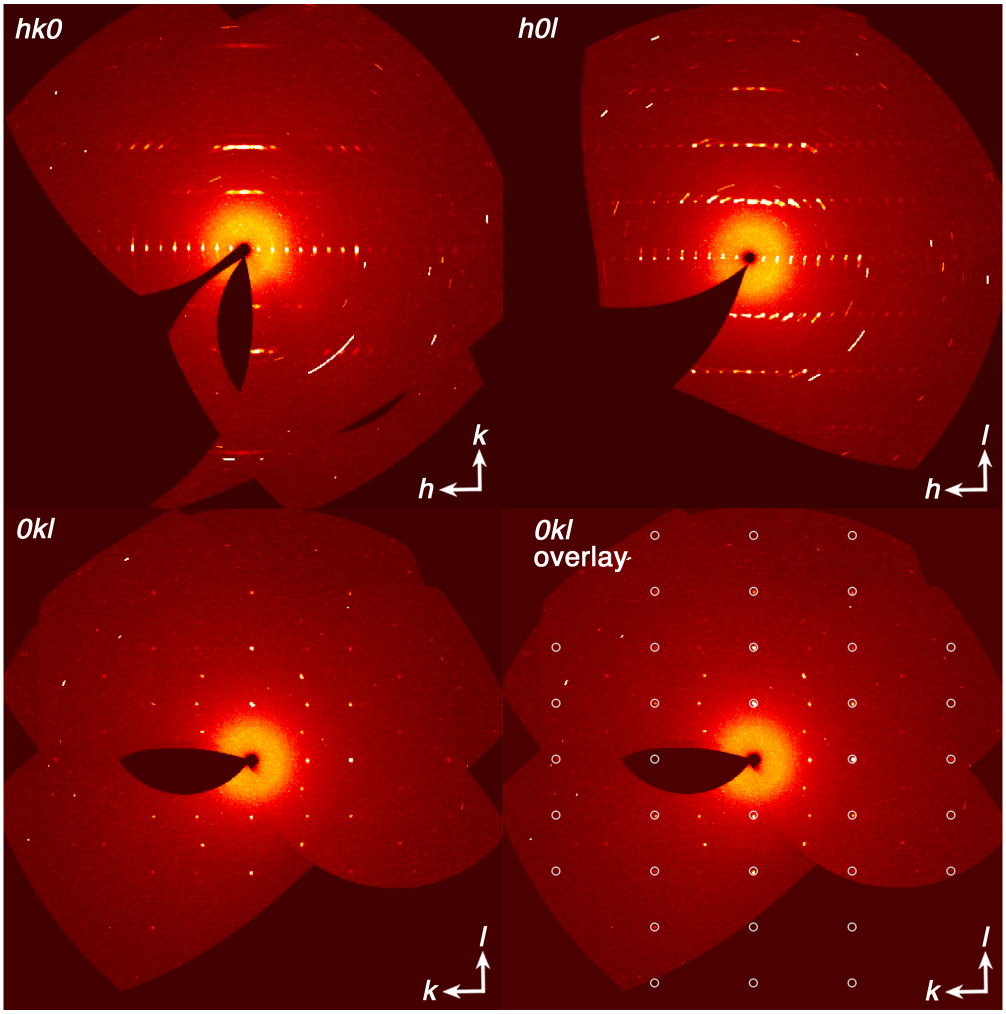

Supplementary Figure 6: Precession images for structure three. The $hk0$ plane is provided with and without an overlay to help differentiate the reflections from the main domain and those from a twin domain. Some smearing of reflection peaks is visible, indicating some degree of polycrystalline or stacking disorder in the crystal.

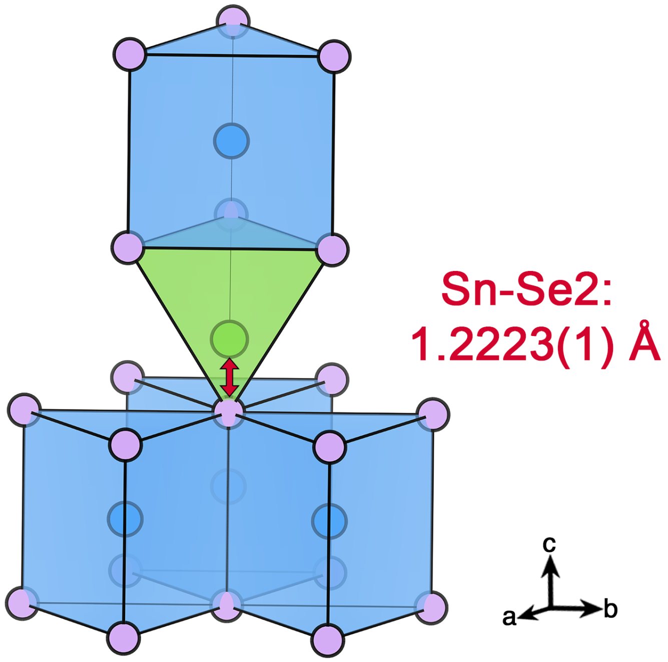

Supplementary Figure 7: Tetrahedral voids between two Se_{180° layers in structure two ($Sn_{0.16}TaSe_{2.0}$), showing the short Sn–Se distance that makes Sn occupation unlikely when Se2 is occupied.

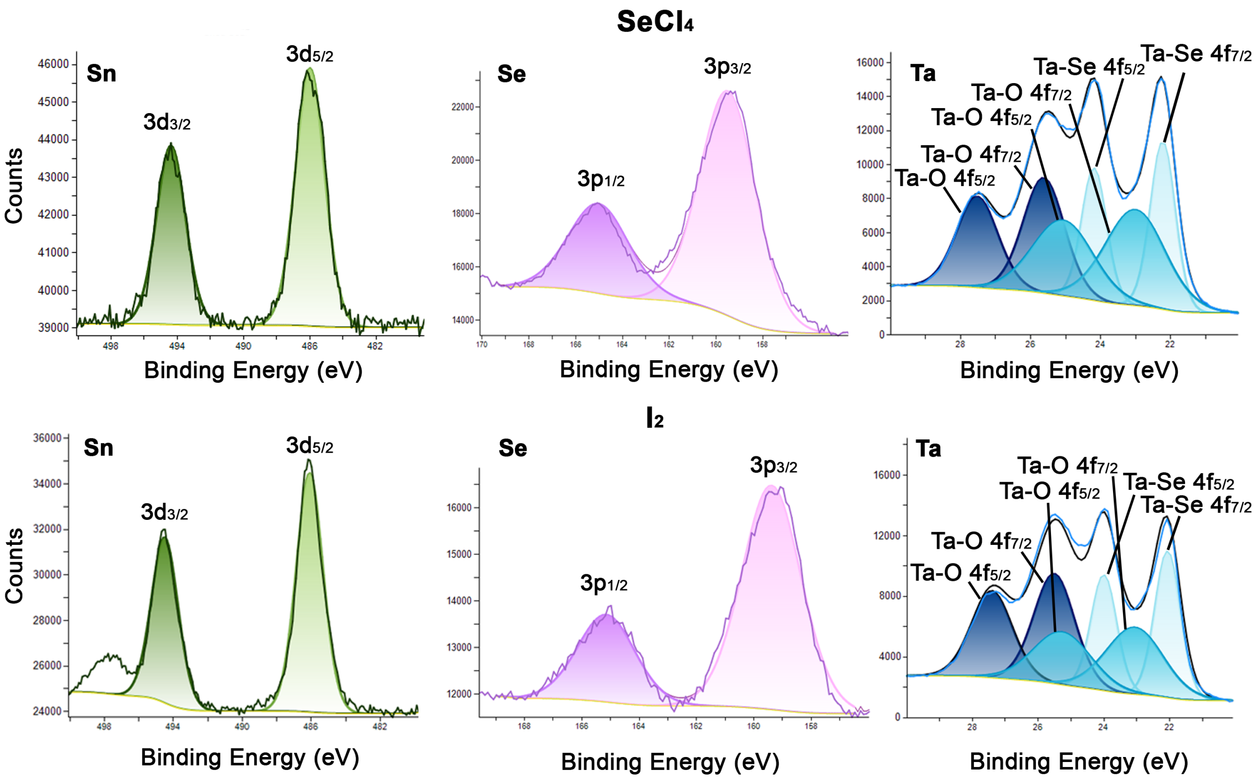

Supplementary Figure 8: XPS for I_2 and $SeCl_4$ grown Sn_xTaSe_2.

Peaks are assigned according to literature.[34–38] Current literature is inconsistent for peak fittings for $TaSe_2$ and related intercalated phases.[38–56] This leads to uncertainty in the assignments, particularly for Ta. We suspect that the Ta-O peaks are primarily from surface oxidation of the samples.

Image Resolution: 512 by 384
Image Pizel Size: 8.08 μm
Acc. Voltage: 21.0 kV
Magnification: 29

$Sn_{1.14}TaSe_{2.54}$

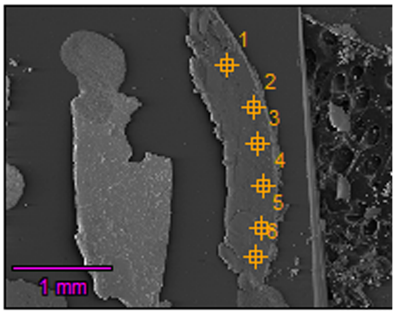

Atomic %	Se (Kα)	Sn (Lα)	Ta (Lα)
1	53.81	25.29	20.90
2	54.24	24.33	21.43
3	53.62	25.01	21.38
4	54.14	24.08	21.78
5	55.90	22.43	21.68
6	53.57	25.52	20.90
Avg.	54.21	24.44	21.35

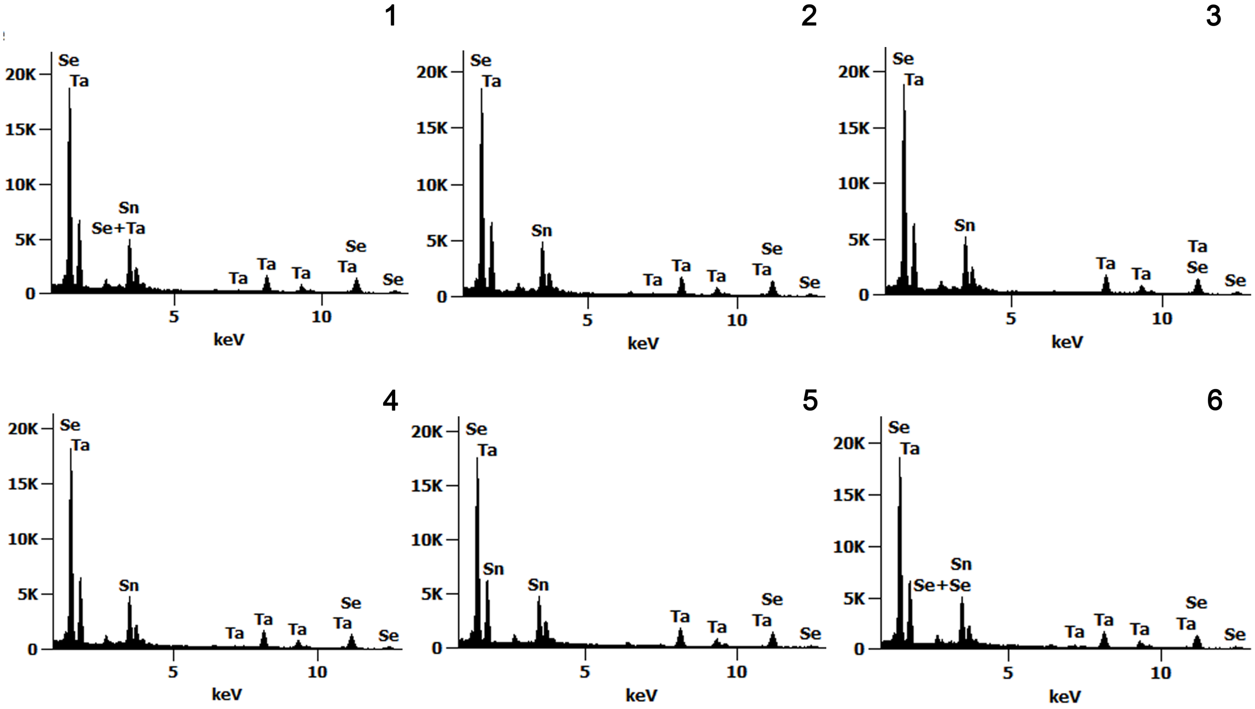

Supplementary Figure 9: Example EDS spectra for Sn_xTaSe_2 crystal.

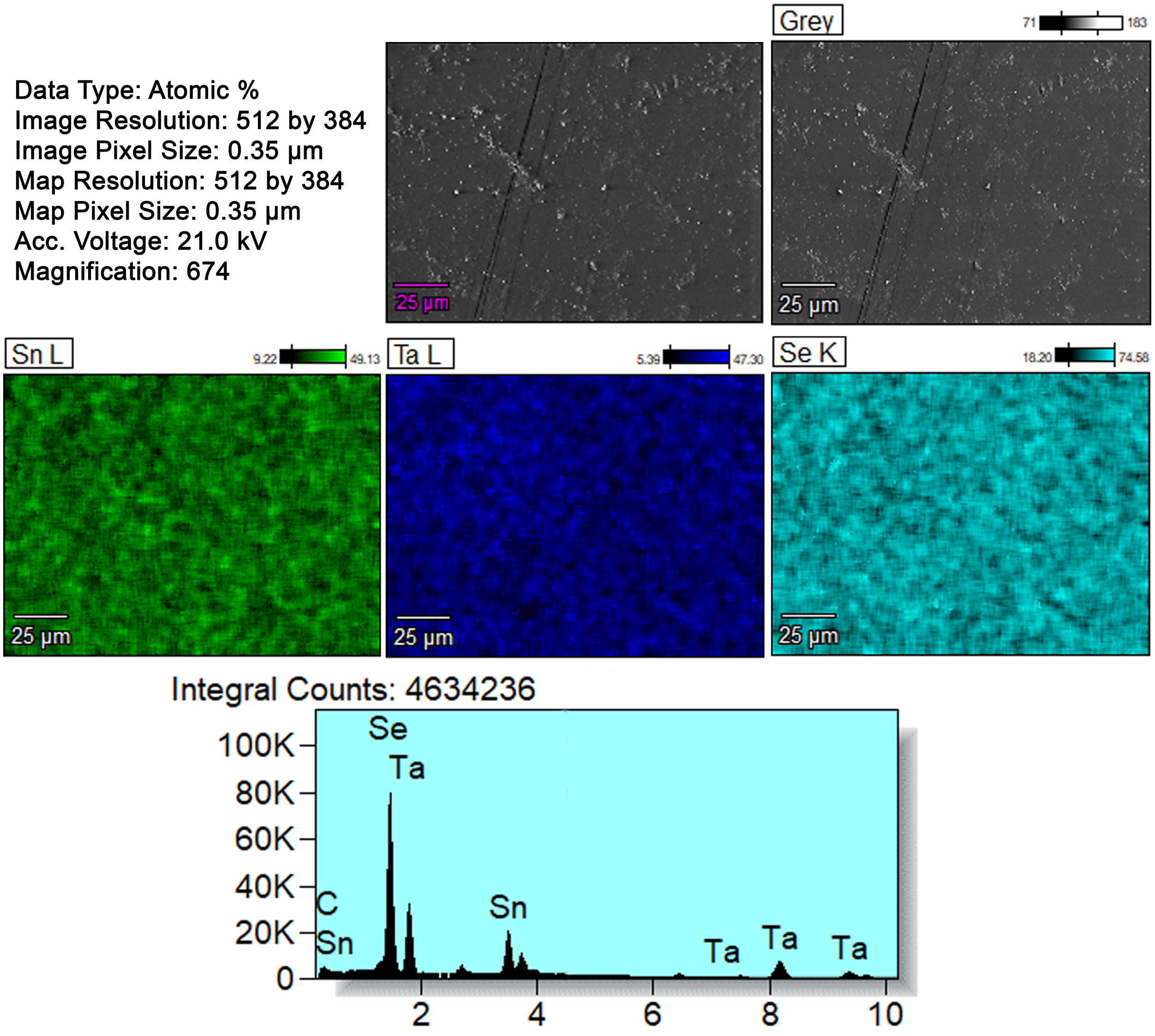

Supplementary Figure 10: EDS atomic percentage map for Sn_xTaSe_2 crystal.

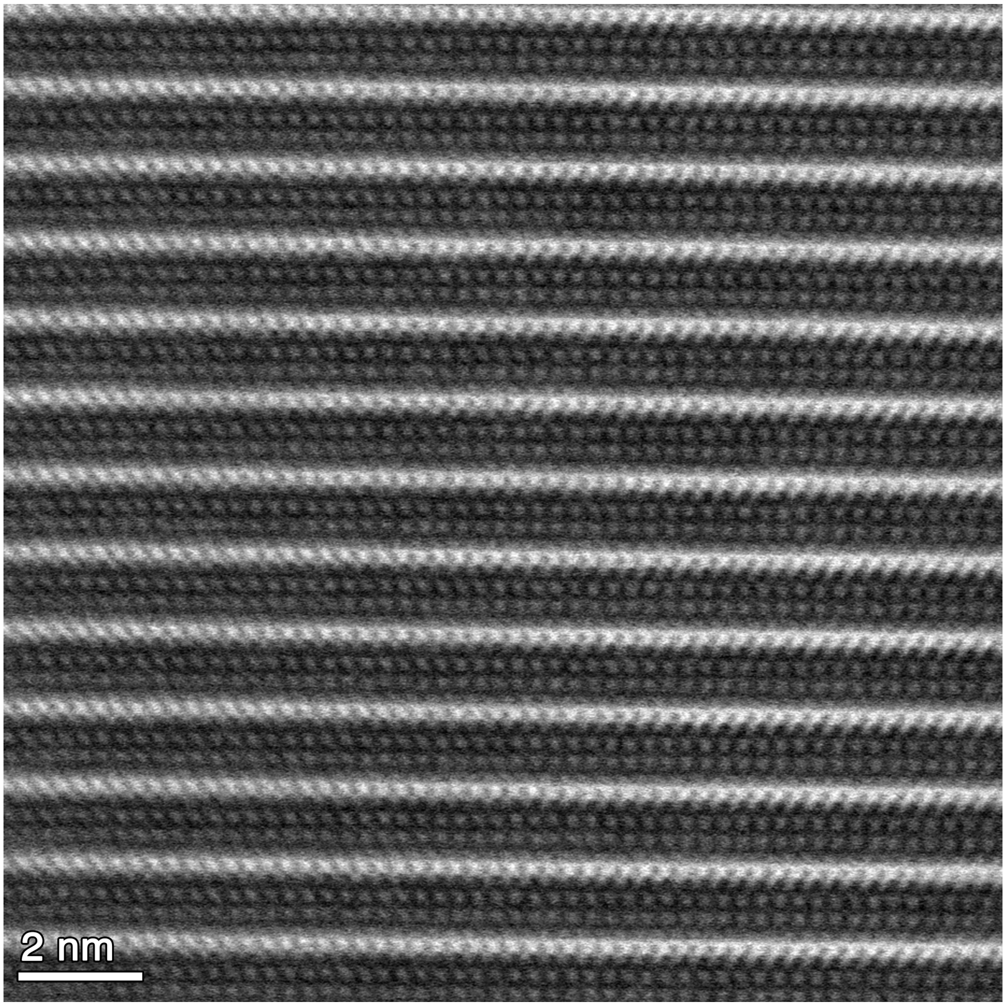

Supplementary Figure 11: Cross-sectional STEM image showing the periodic region composed of Ta-centered trigonal prisms alternated with two layers of Sn. In this region, the Sn atoms are visible as distinguishable circles rather than lines. Note: The limited resolution of the $TaSe_2$ layers means their orientation cannot be determined unambiguously.

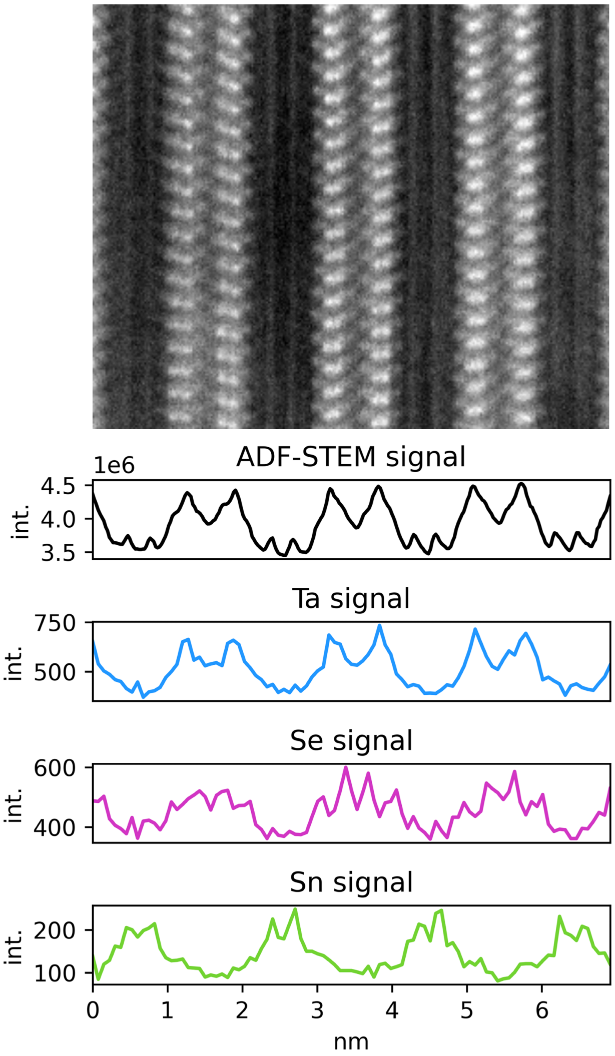

Supplementary Figure 12: Cross-sectional STEM image with collected EDS signal.

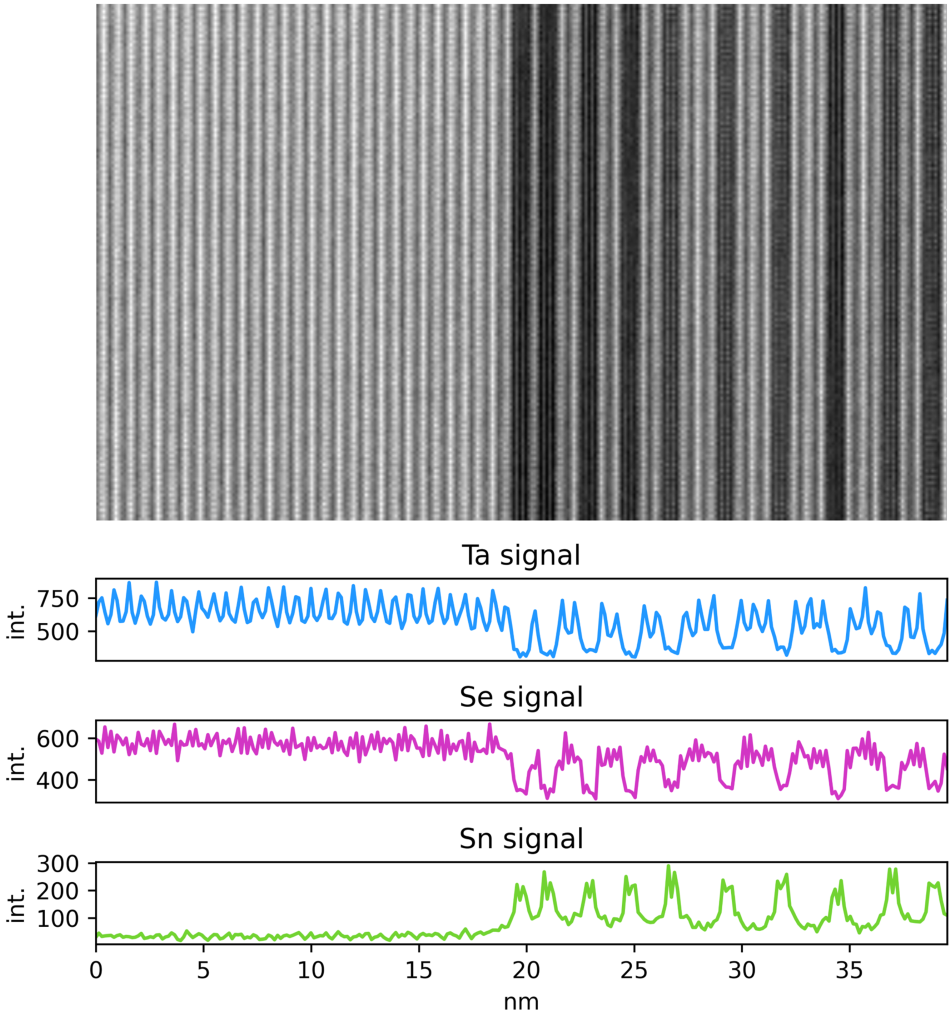

Supplementary Figure 13: Cross-sectional STEM image with collected EDS signal in a transition region from periodic to variable stacking.

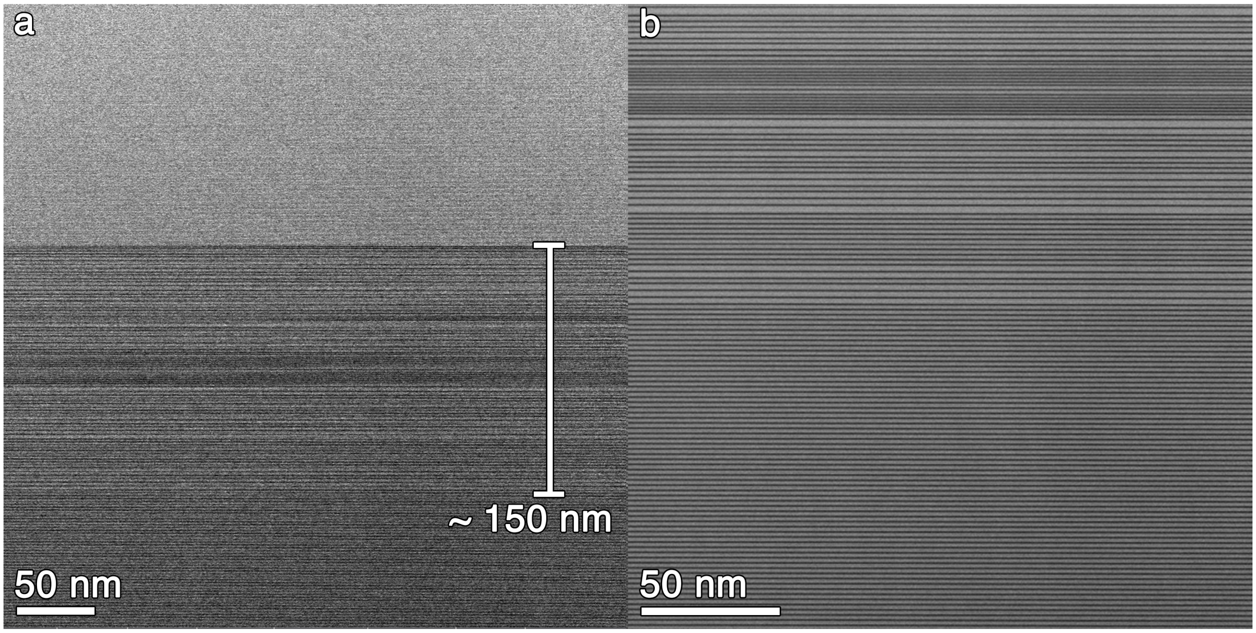

Supplementary Figure 14: Cross-sectional STEM image showing: (a) full region of variable stacking, marked by the white bars and (b) the transition from variable stacking to a periodic region composed of two layers of $TaSe_2$ alternated with two layers of Sn channels.

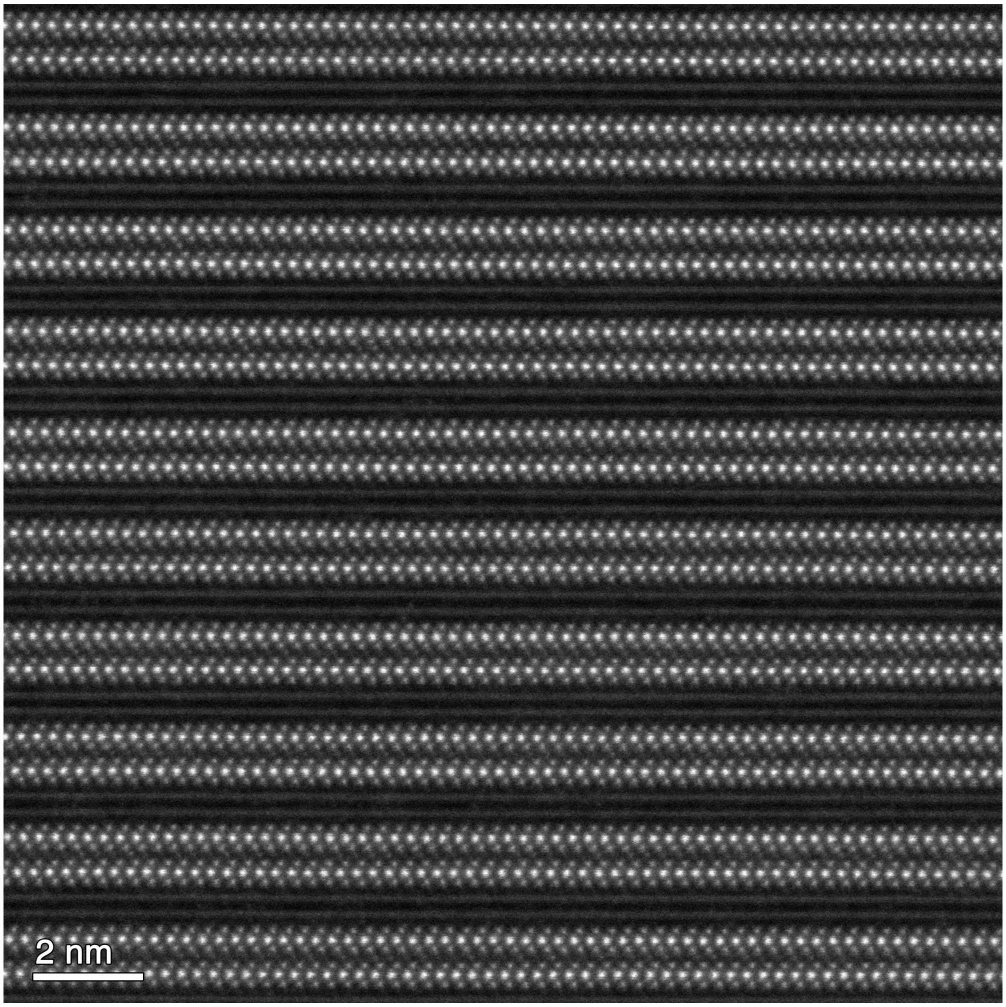

Supplementary Figure 15: Cross-sectional STEM image showing the stacking pattern that stabilized after the variable stacking transition. Note: diffuse intercalants between $TaSe_2$ layers appear to be visible.

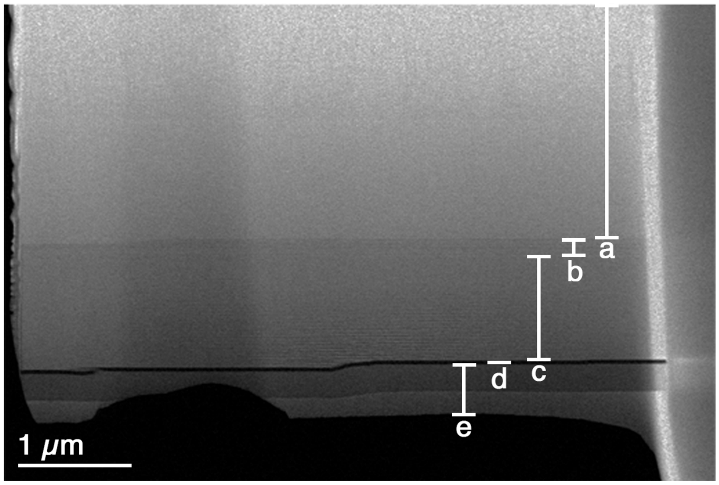

Supplementary Figure 16: Cross-sectional STEM image showing the full range view of the STEM sample obtained from the flake corresponding to structure two. The five regions of the structure are marked with bars, with the label underneath: (a) Periodic stacking of Ta-centered trigonal prisms with diffuse intercalants between layers, (b) region of variable stacking, (c) periodic stacking of two layers of Ta-centered trigonal prisms alternated with two layers of Sn, (d) amorphous carbon, and (e) protective Pt.

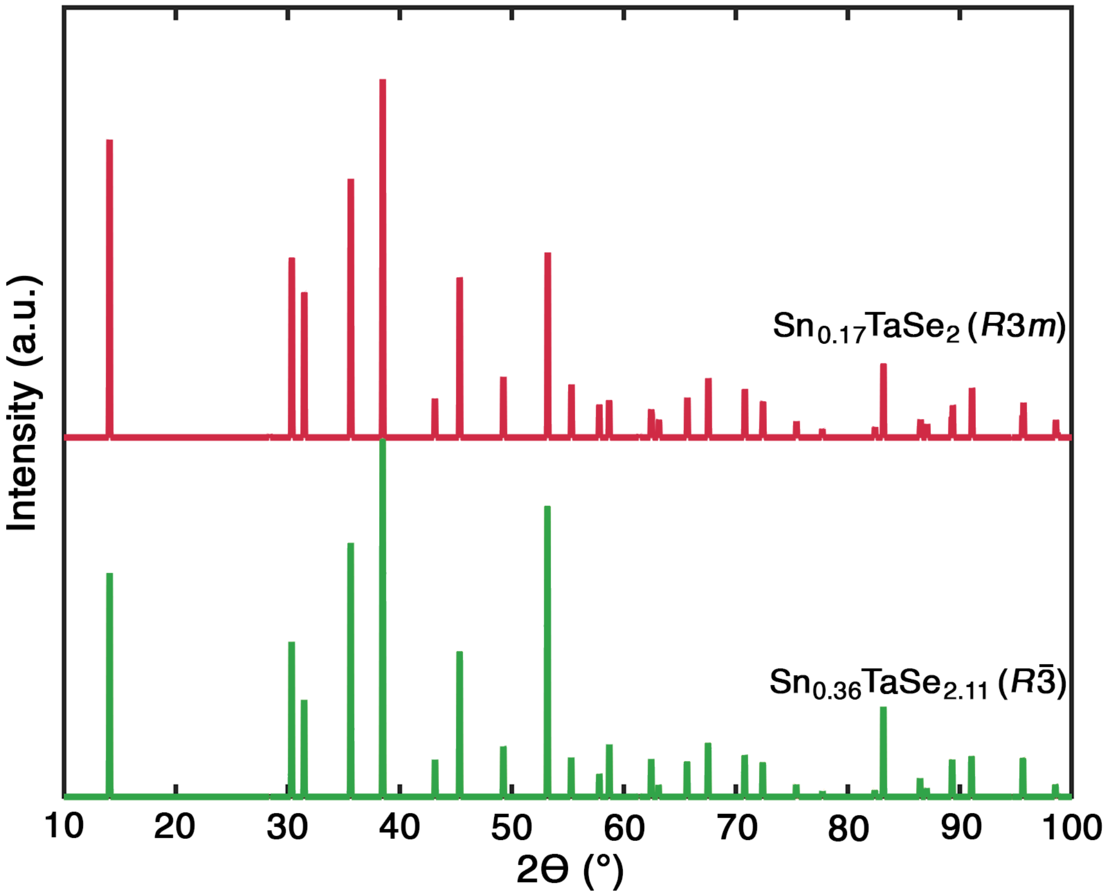

Supplementary Figure 17: Calculated PXRD patterns for two Sn_xTaSe_2 structures with effectively identical peak positions but different structures. The two structures are theoretical; phase data and atomic parameters for these two structures are given in Table S10-S13.

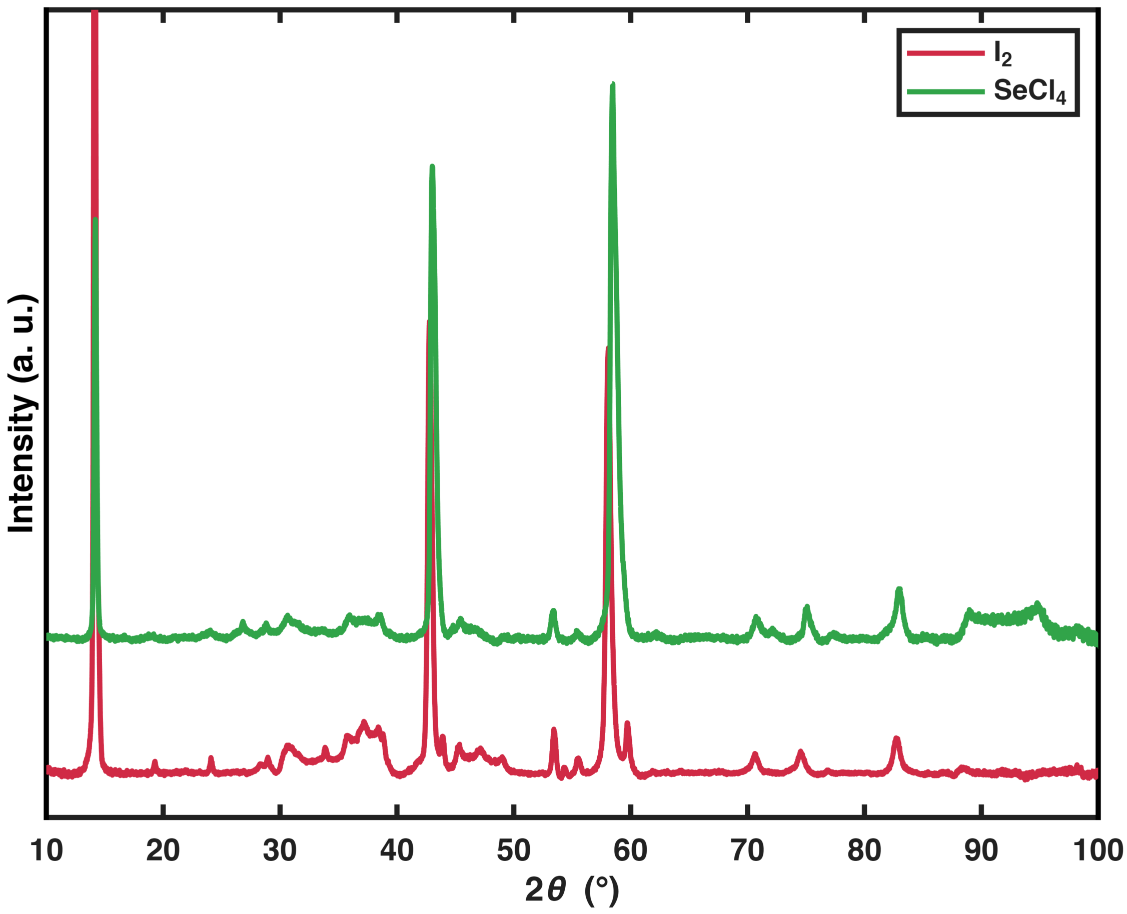

Supplementary Figure 18: Experimental PXRD patterns for I_2 and $SeCl_4$ grown samples of Sn_xTaSe_2 showing a high degree of preferred orientation. The c-axis is the out of plane direction of the physical plates, resulting in more intense $(00l)$ peaks. The patterns feature sharp $(00l)$ peaks and anisotropic broadening on $(h0l)$ and $(0kl)$ peaks, indicating the presence of stacking disorder.[57]

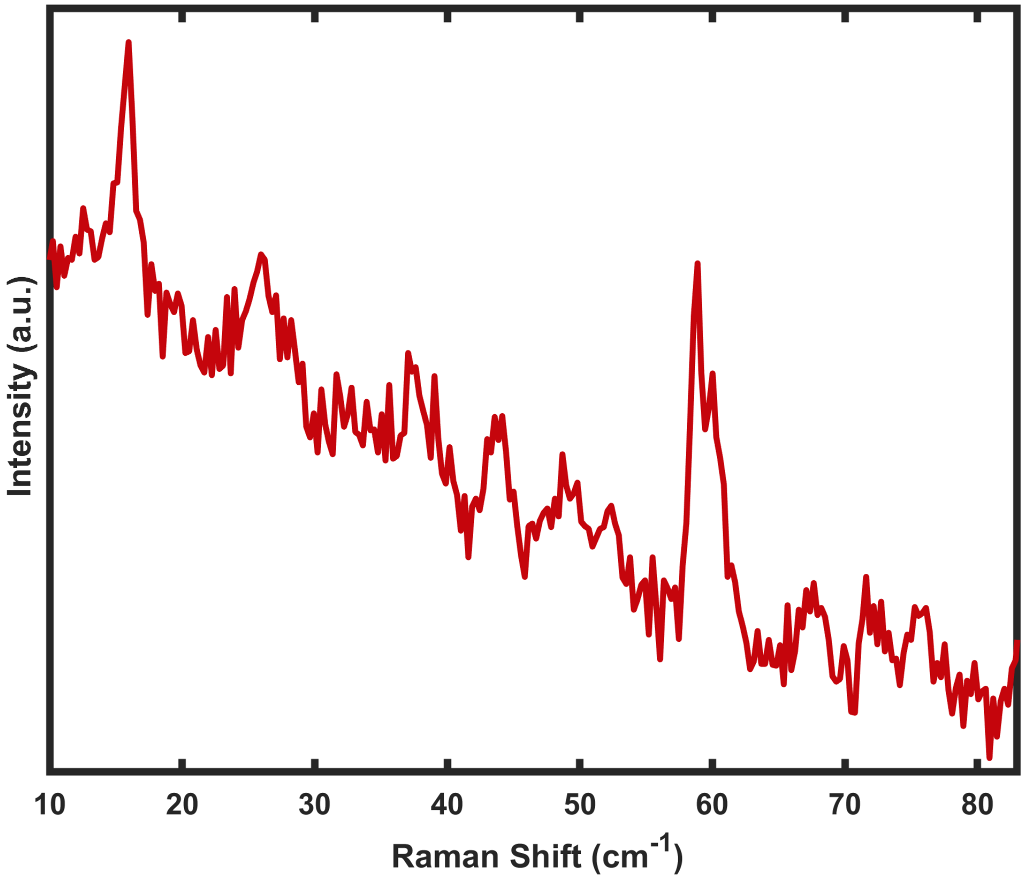

Supplementary Figure 19: Low-frequency Raman spectrum for SiO_2 without baseline correction. This spectrum was subtracted from the low frequency Sn_xTaSe_2 spectra included in the main text.

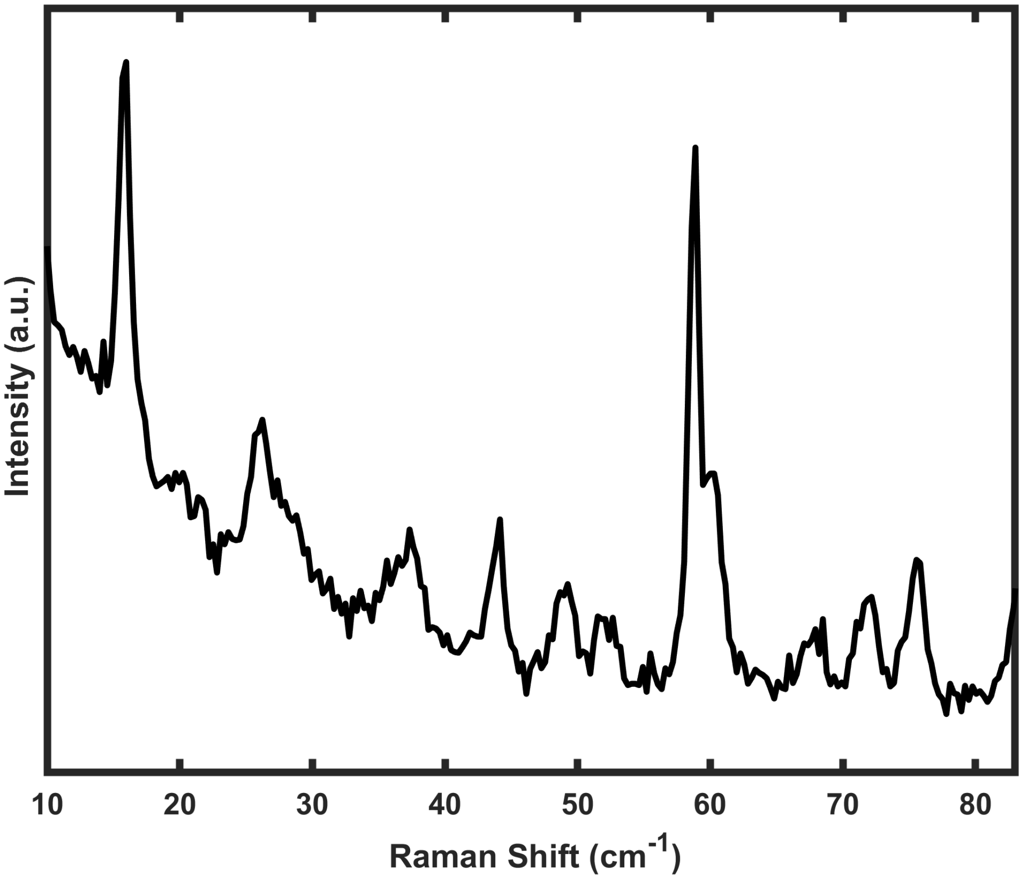

Supplementary Figure 20: Low-frequency Raman spectrum for $TaSe_2$ without baseline correction or SiO_2 subtraction.

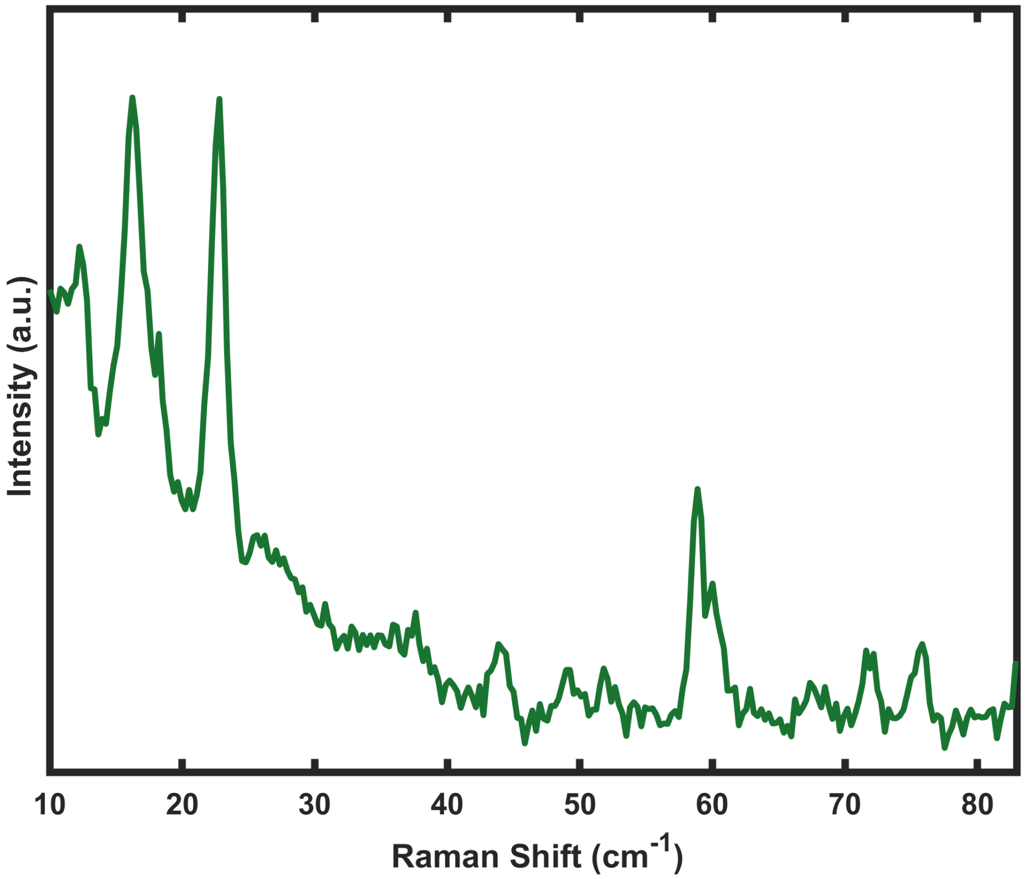

Supplementary Figure 21: Low-frequency Raman spectrum for $Sn_{0.81}TaSe_{2.20}$ without baseline correction or SiO_2 subtraction.

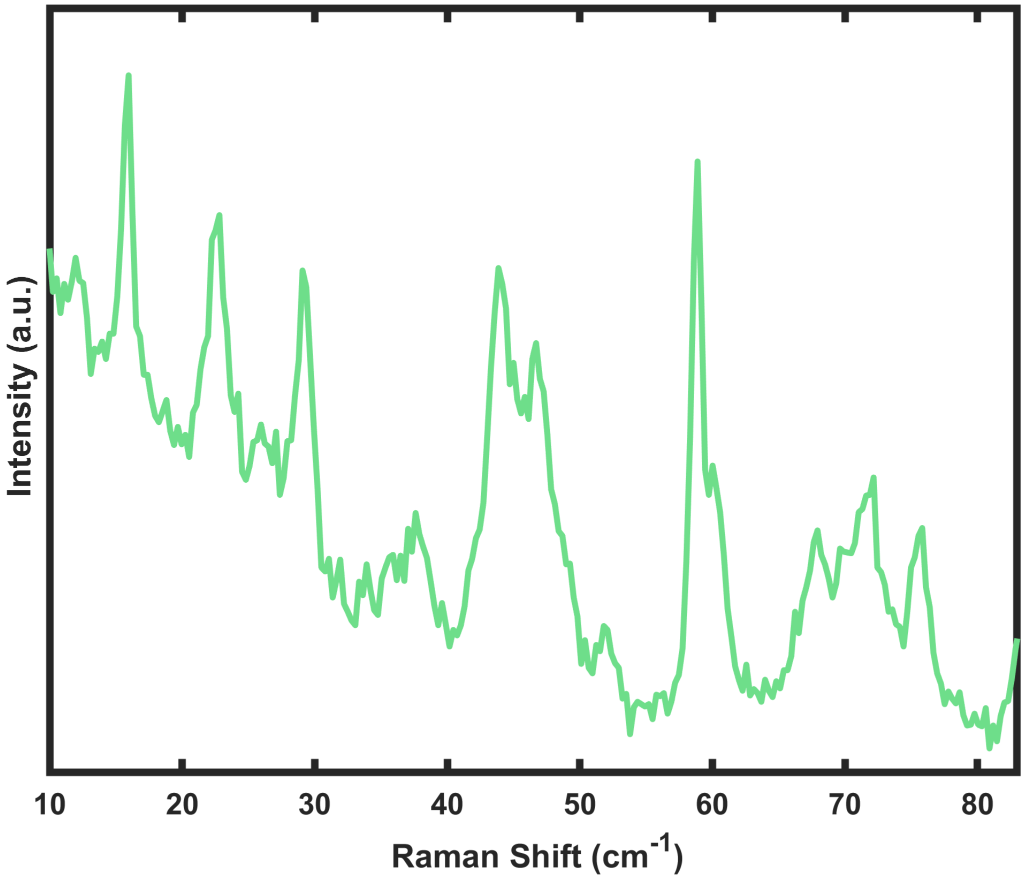

Supplementary Figure 22: Low-frequency Raman spectrum for $Sn_{0.29}TaSe_{1.82}$ without baseline correction or SiO_2 subtraction.

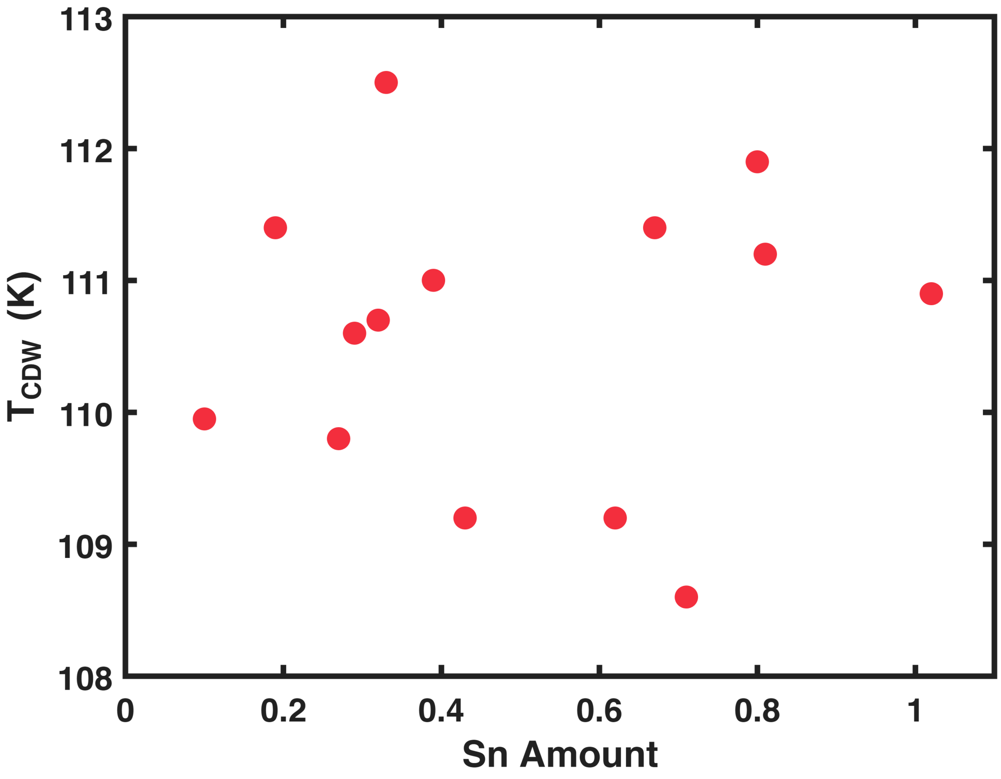

Supplementary Figure 23: T_{CDW} plotted against the amount of Sn

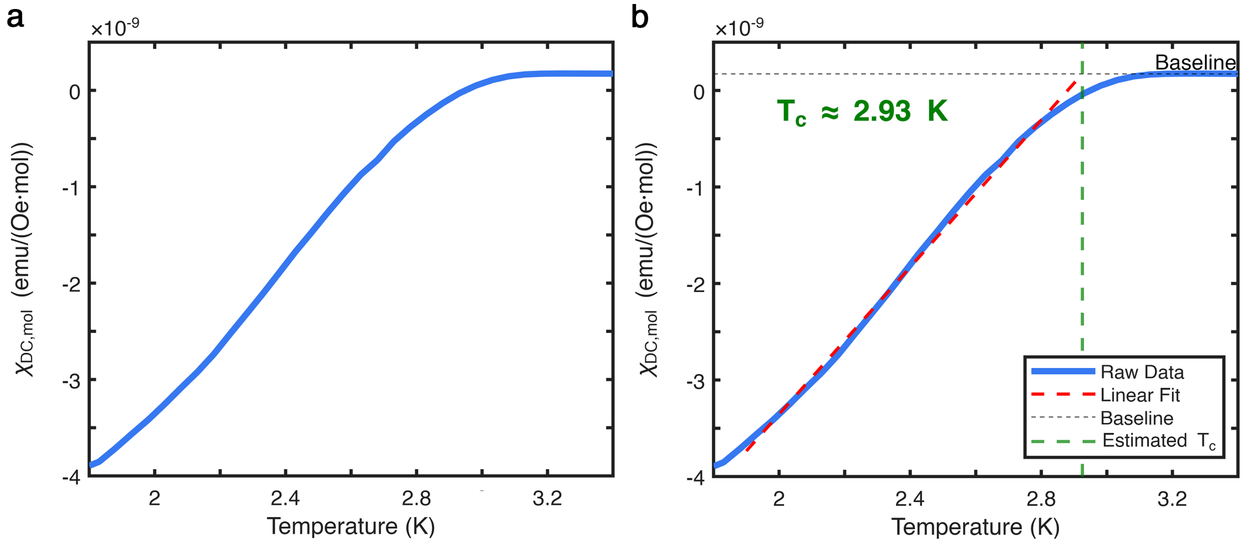

Supplementary Figure 24: Low-temperature field-cooled magnetic moment measurements across the superconducting transition of Sn_xTaSe_2: (a) unfitted and (b) fitted curve for 1.8 to 3.4 K. The molar mass used to calculate $X_{DC,mol}$ was 398.2 g/mol ($Sn_{0.5}TaSe_2$).

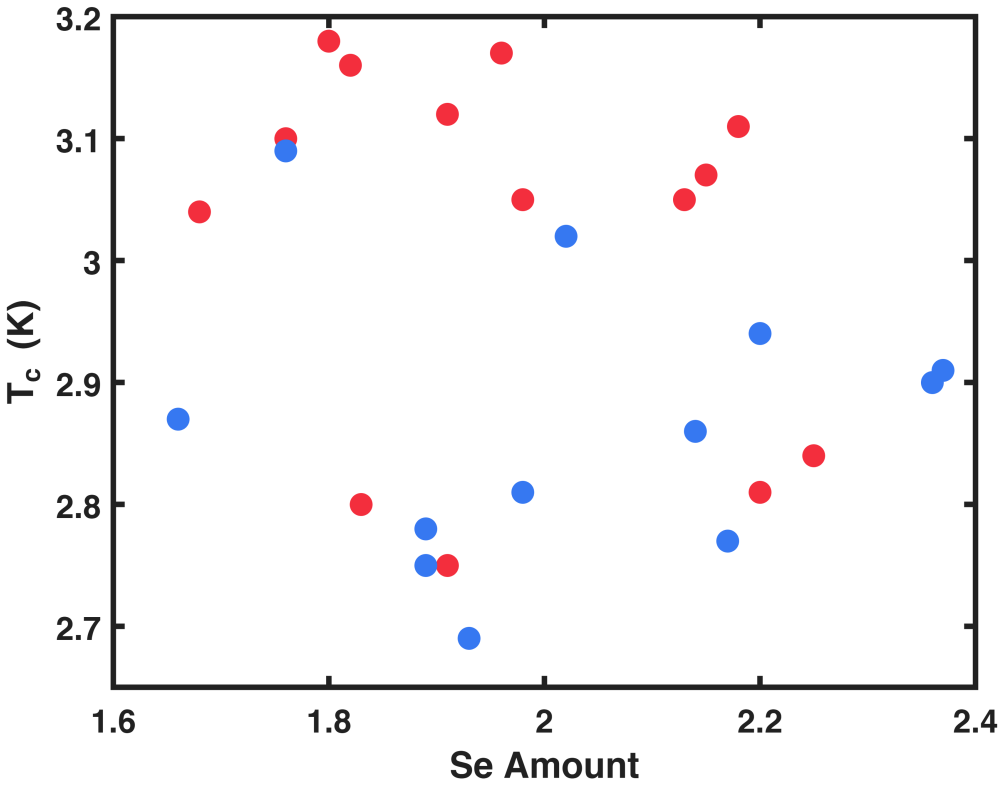

Supplementary Figure 25: Correlation between T_c and the stoichiometric amount of Se. Points in red are from samples with visible CDW. Points in blue are from samples without a visible CDW.

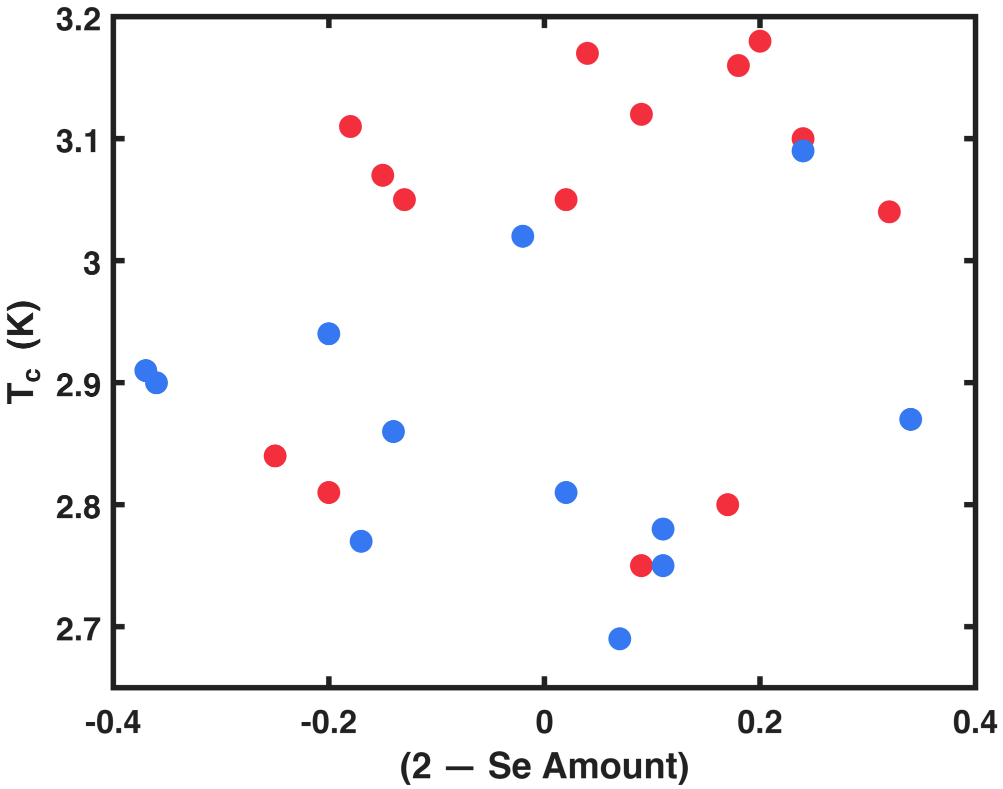

Supplementary Figure 26: Correlation between T_c and the difference in the Se amount from two. Points in red are from samples with visible CDW. Points in blue are from samples without a visible CDW.

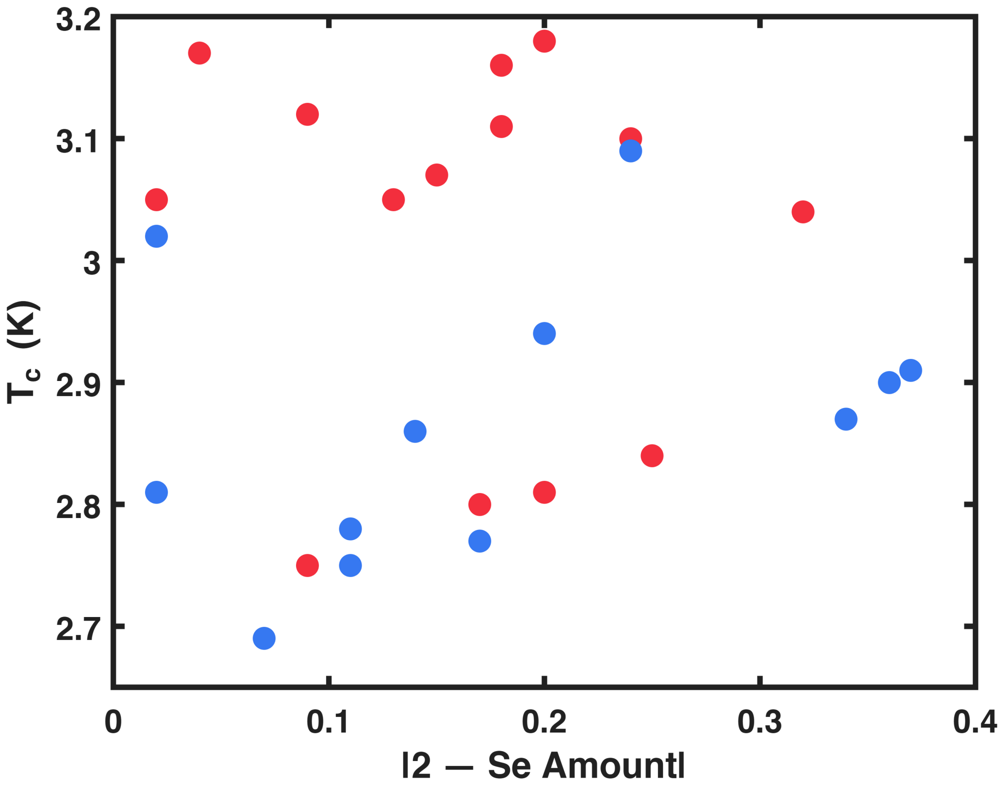

Supplementary Figure 27: Correlation between T_c and the absolute value of the difference in the Se amount from two. Points in red are from samples with visible CDW. Points in blue are from samples without a visible CDW.

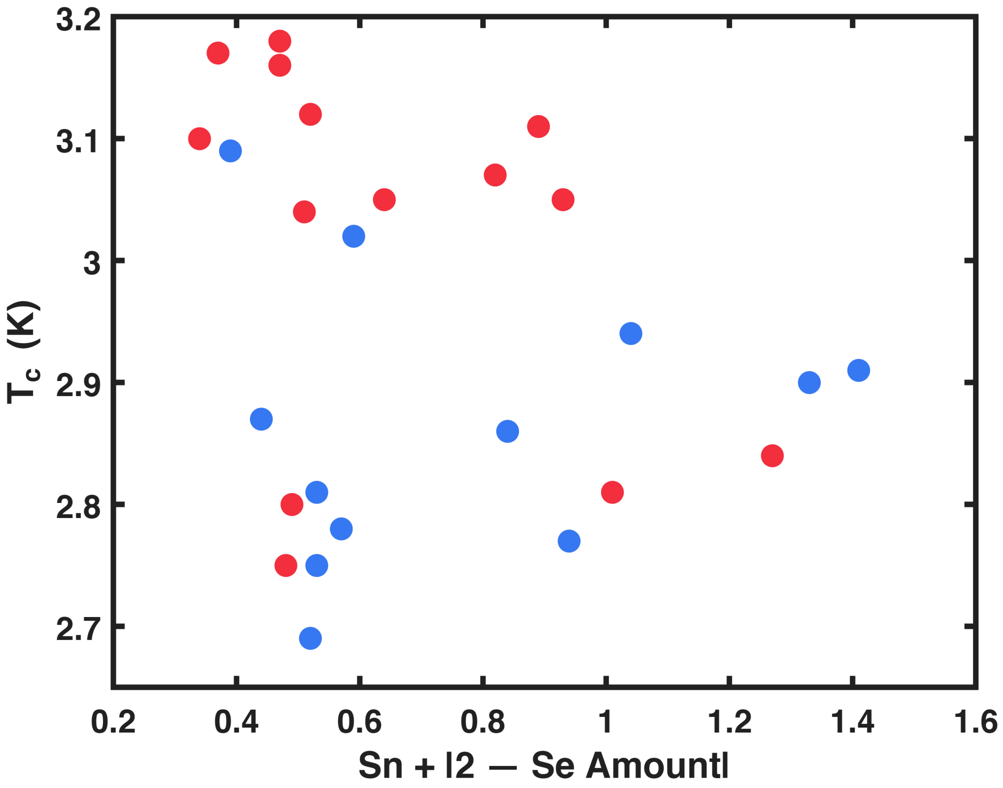

Supplementary Figure 28: Correlation between T_c and the sum of the amount of Sn and the absolute value of the difference in the Se amount from two. Points in red are from samples with visible CDW. Points in blue are from samples without a visible CDW.

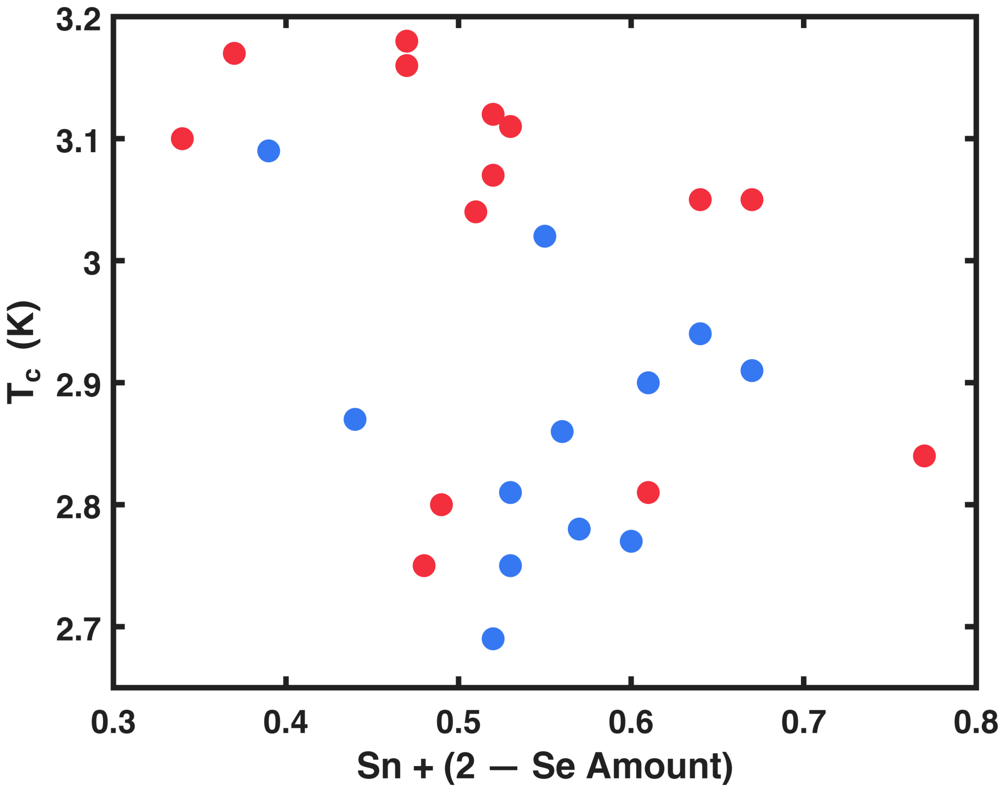

Supplementary Figure 29: Correlation between T_c and the sum of the amount of Sn and the difference in the Se amount from two. Points in red are from samples with visible CDW. Points in blue are from samples without a visible CDW.

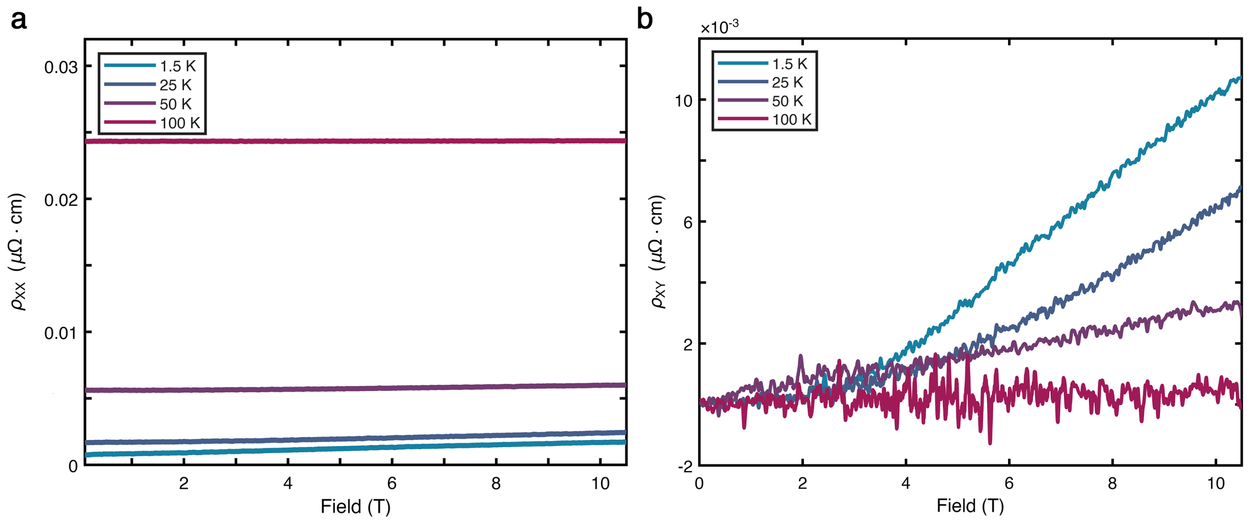

Supplementary Figure 30: Magnetotransport for $2H$-$TaSe_2$. (a) Longitudinal resistivity (ρ_{xx}). (b) Hall response (ρ_{xy}).

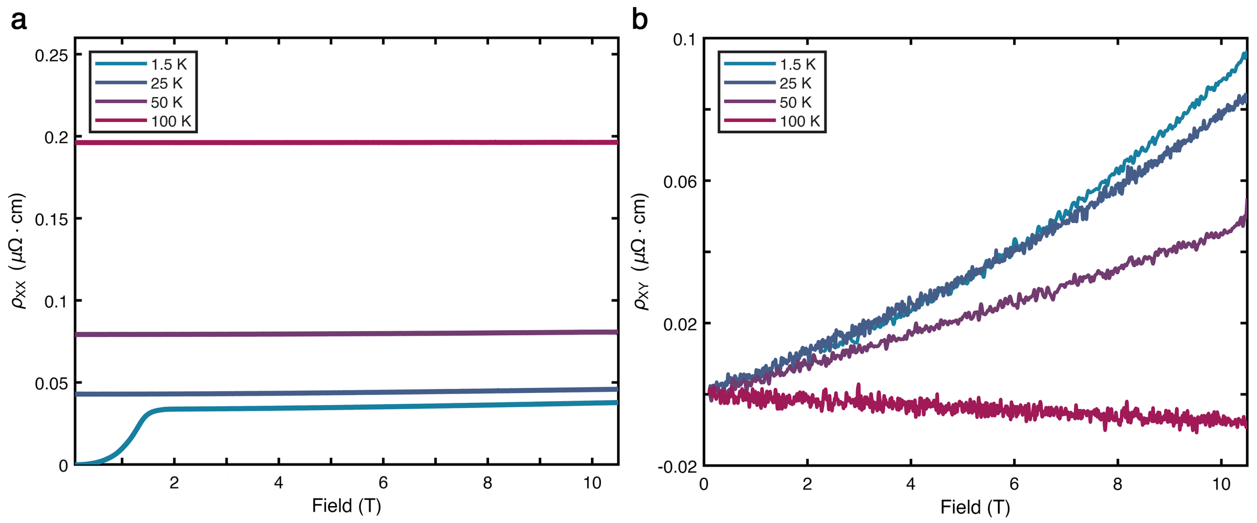

Supplementary Figure 31: Magnetotransport for $Sn_{0.81}TaSe_{2.20}$. (a) Longitudinal resistivity (ρ_{xx}). (b) Hall response (ρ_{xy}).

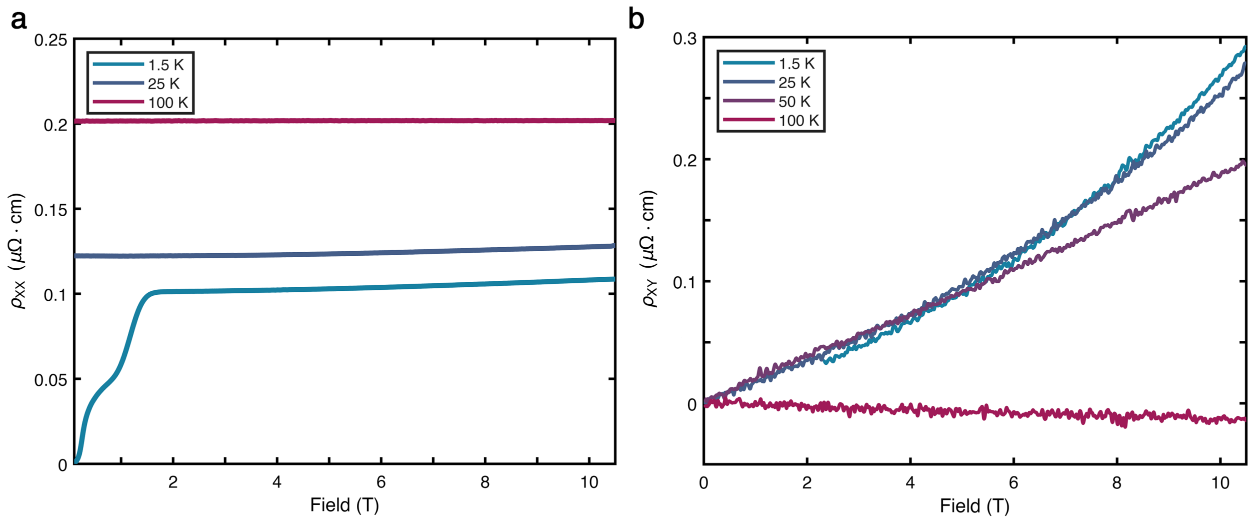

Supplementary Figure 32: Magnetotransport for $Sn_{0.43}TaSe_{1.91}$. (a) Longitudinal resistivity (ρ_{xx}). (b) Hall response (ρ_{xy}).

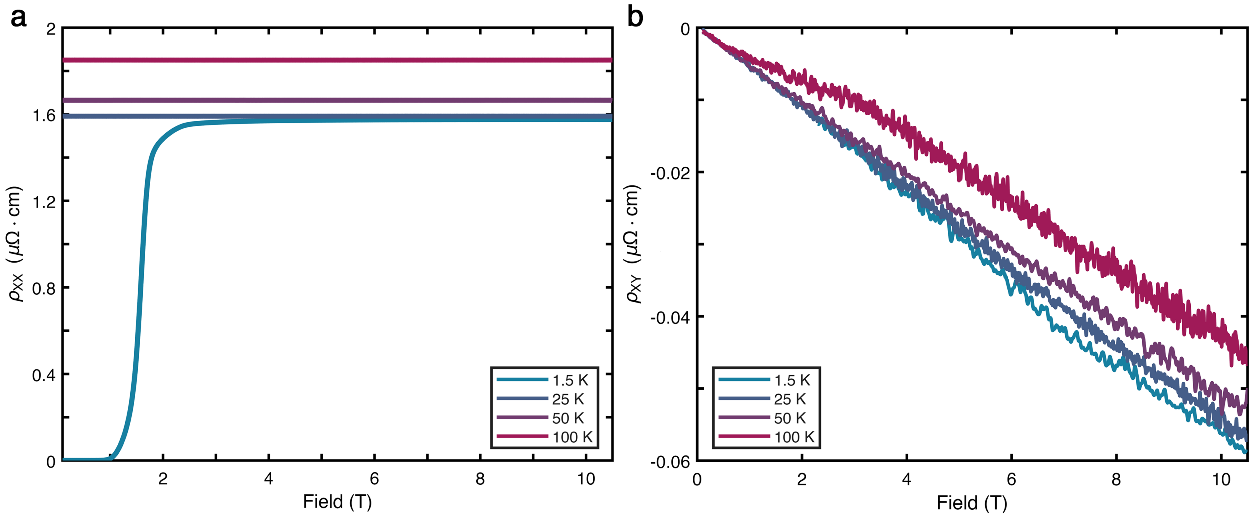

Supplementary Figure 33: Magnetotransport for $Sn_{0.15}TaSe_{1.76}$. (a) Longitudinal resistivity (ρ_{xx}). (b) Hall response (ρ_{xy}).

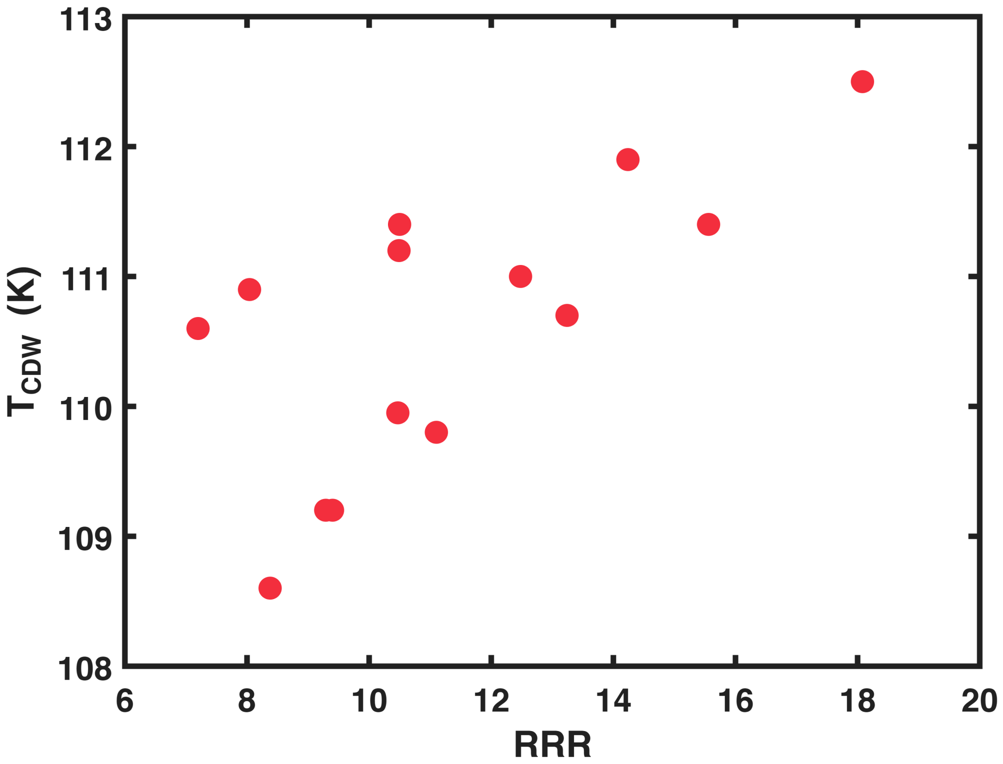

Supplementary Figure 34: Correlation between T_{CDW} and RRR of $\mathrm{Sn}_x\mathrm{TaSe}_2$ samples.

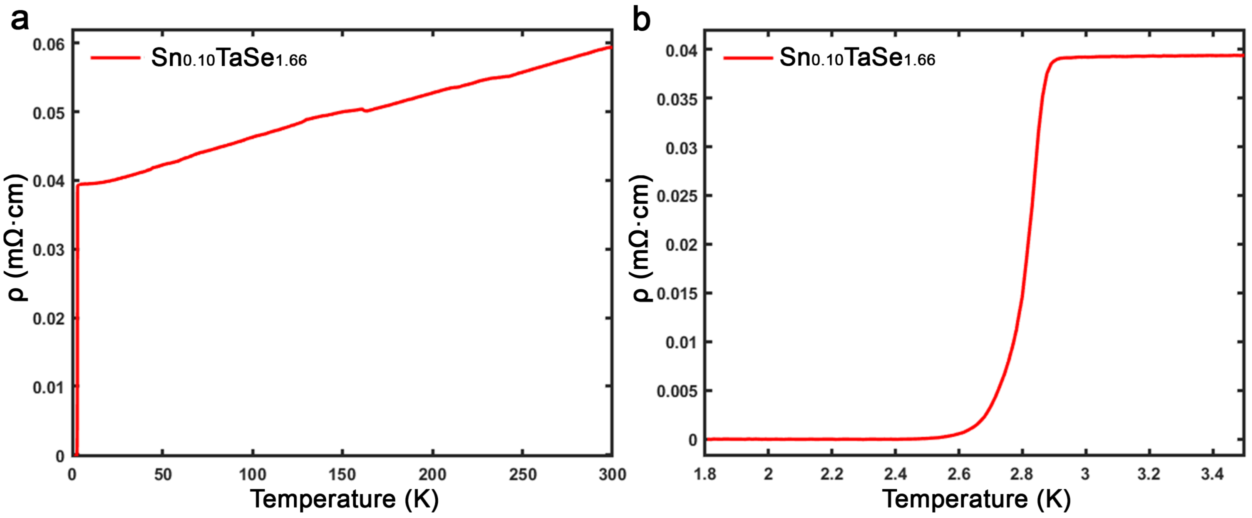

Supplementary Figure 35: Transport curve for $Sn_{0.10}TaSe_{1.66}$. (a) Full temperature range (0 to 300 K). (b) Low-temperature range (1.8 to 3.5 K).

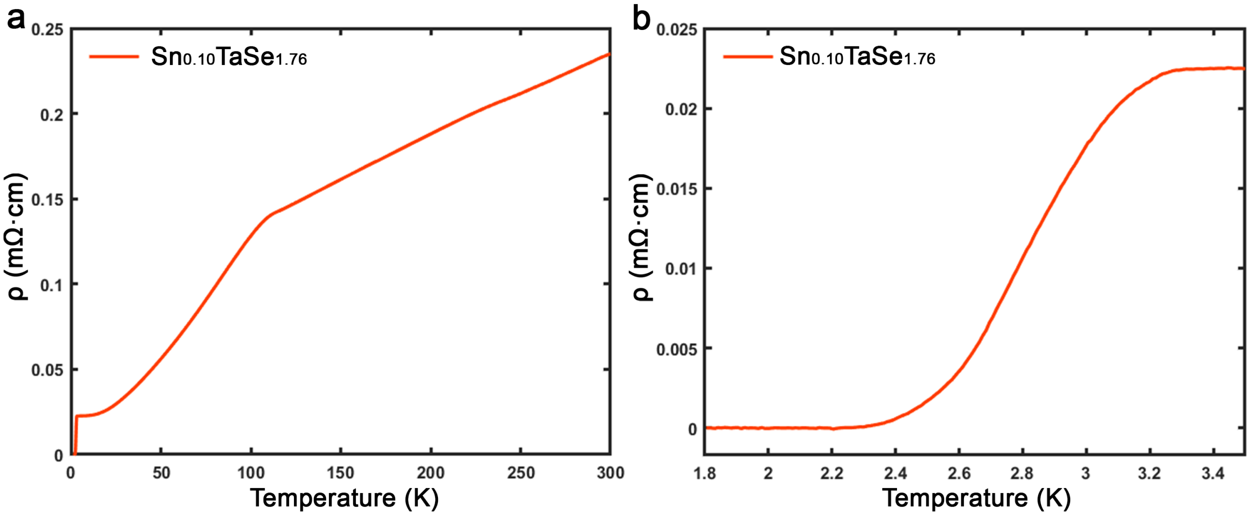

Supplementary Figure 36: Transport curve for $Sn_{0.10}TaSe_{1.76}$. (a) Full temperature range (0 to 300 K). (b) Low-temperature range (1.8 to 3.5 K).

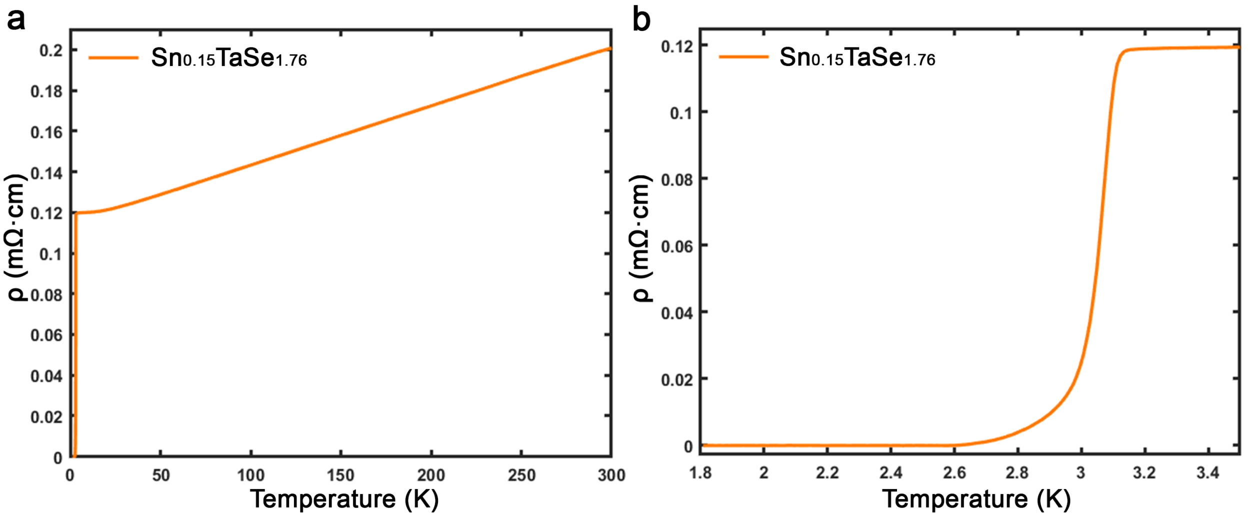

Supplementary Figure 37: Transport curve for $Sn_{0.15}TaSe_{1.76}$. (a) Full temperature range (0 to 300 K). (b) Low-temperature range (1.8 to 3.5 K).

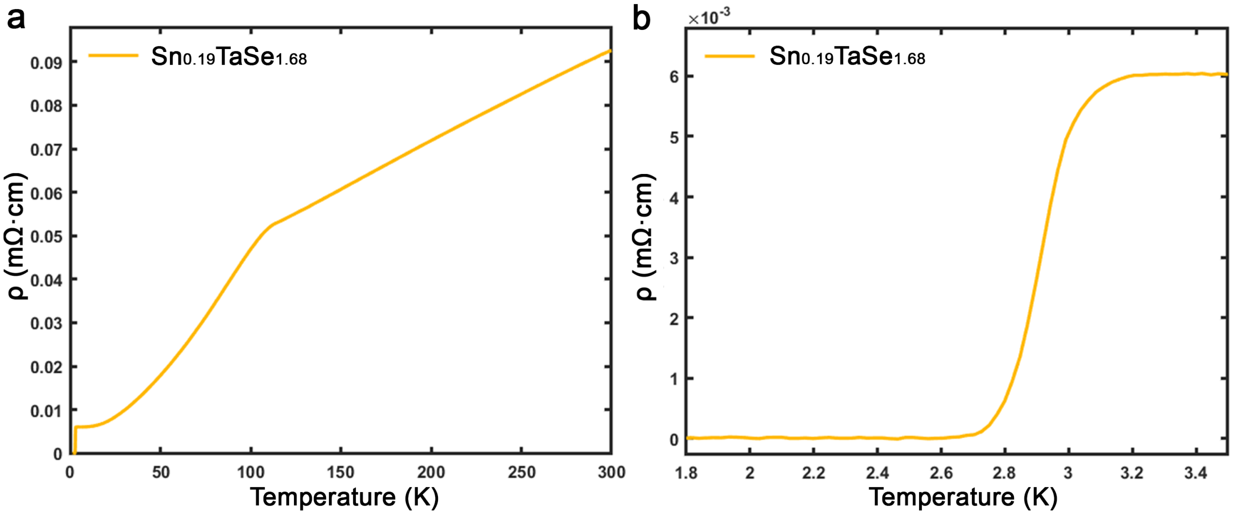

Supplementary Figure 38: Transport curve for $Sn_{0.19}TaSe_{1.68}$. (a) Full temperature range (0 to 300 K). (b) Low-temperature range (1.8 to 3.5 K).

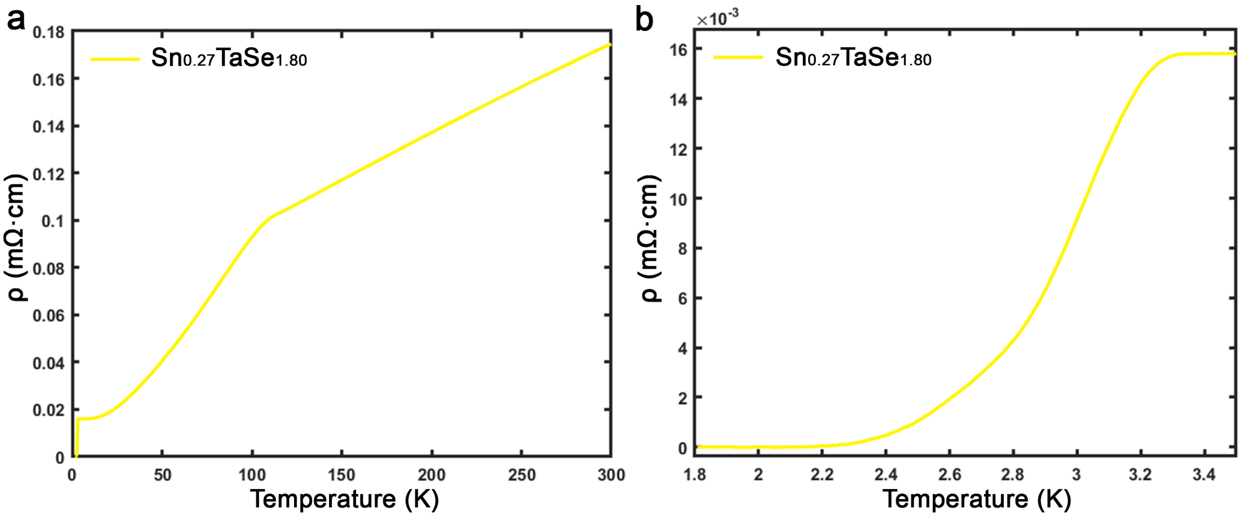

Supplementary Figure 39: Transport curve for $Sn_{0.27}TaSe_{1.80}$. (a) Full temperature range (0 to 300 K). (b) Low-temperature range (1.8 to 3.5 K).

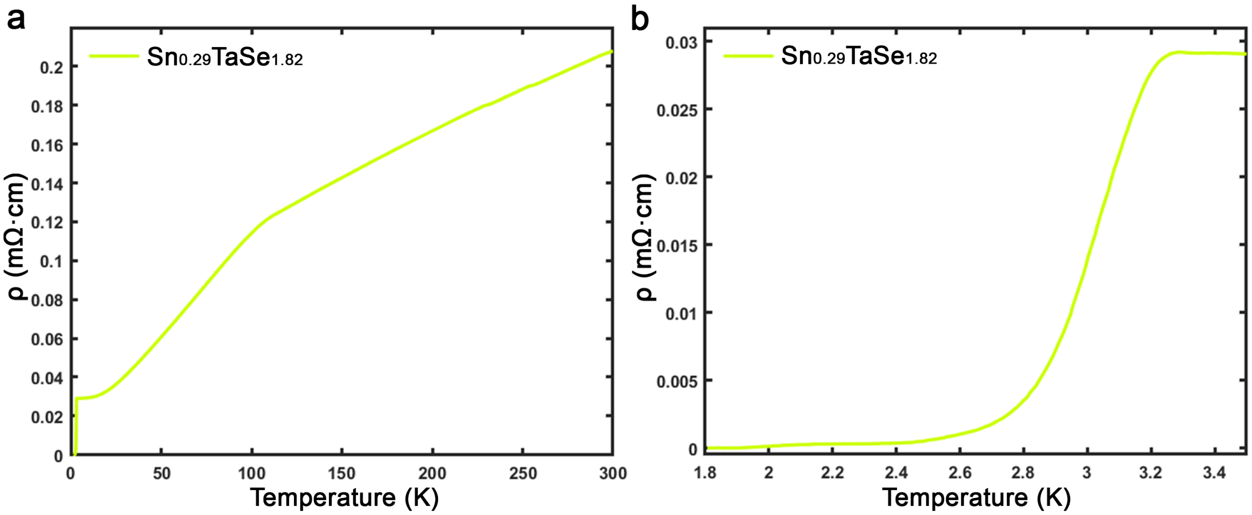

Supplementary Figure 40: Transport curve for $Sn_{0.29}TaSe_{1.82}$. (a) Full temperature range (0 to 300 K). (b) Low-temperature range (1.8 to 3.5 K).

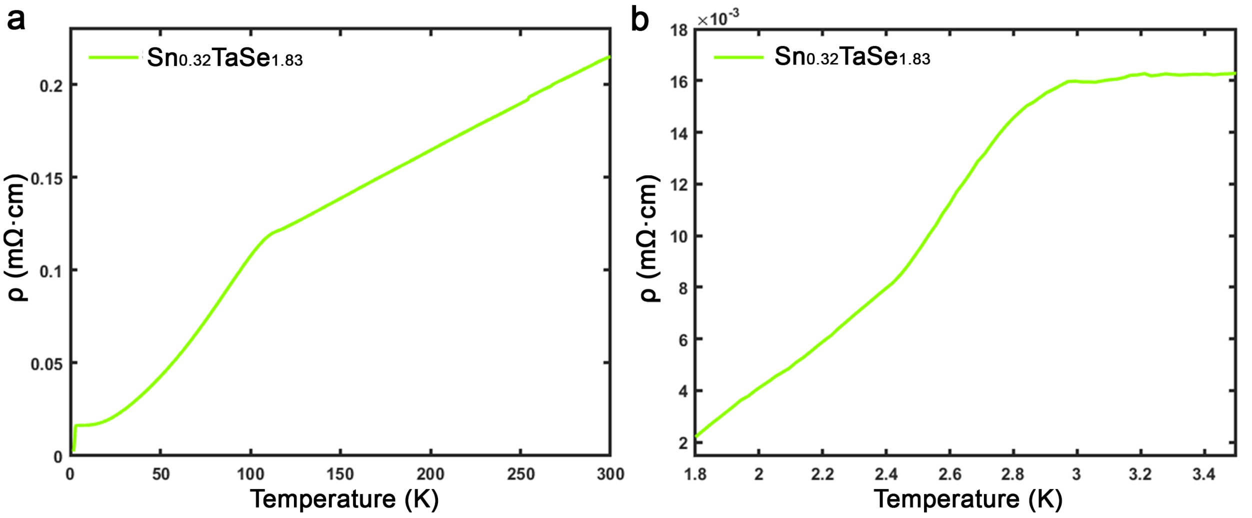

Supplementary Figure 41: Transport curve for $Sn_{0.32}TaSe_{1.83}$. (a) Full temperature range (0 to 300 K). (b) Low-temperature range (1.8 to 3.5 K).

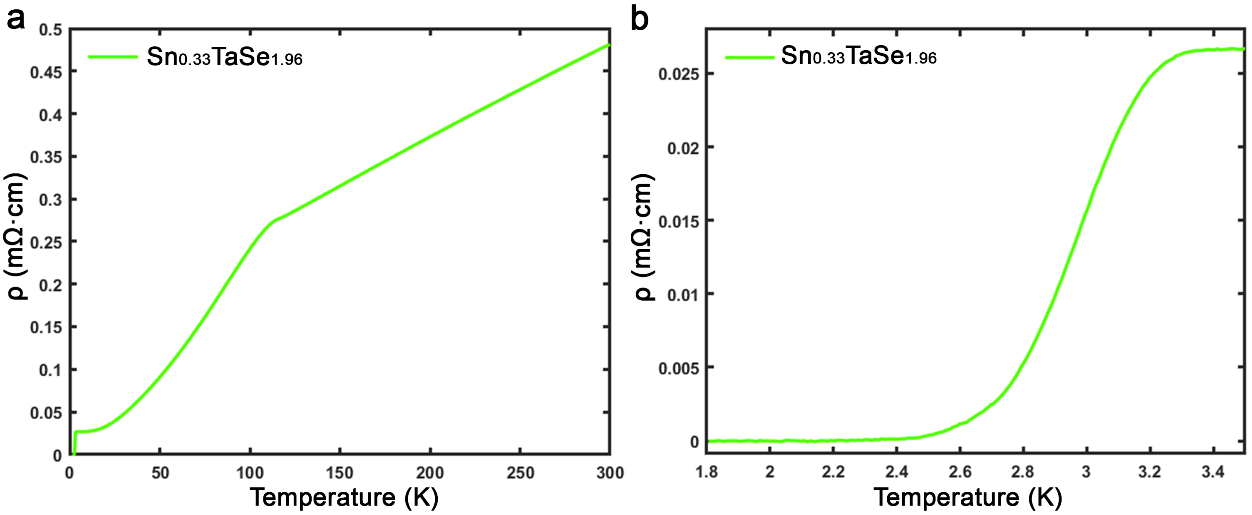

Supplementary Figure 42: Transport curve for $Sn_{0.33}TaSe_{1.96}$. (a) Full temperature range (0 to 300 K). (b) Low-temperature range (1.8 to 3.5 K).

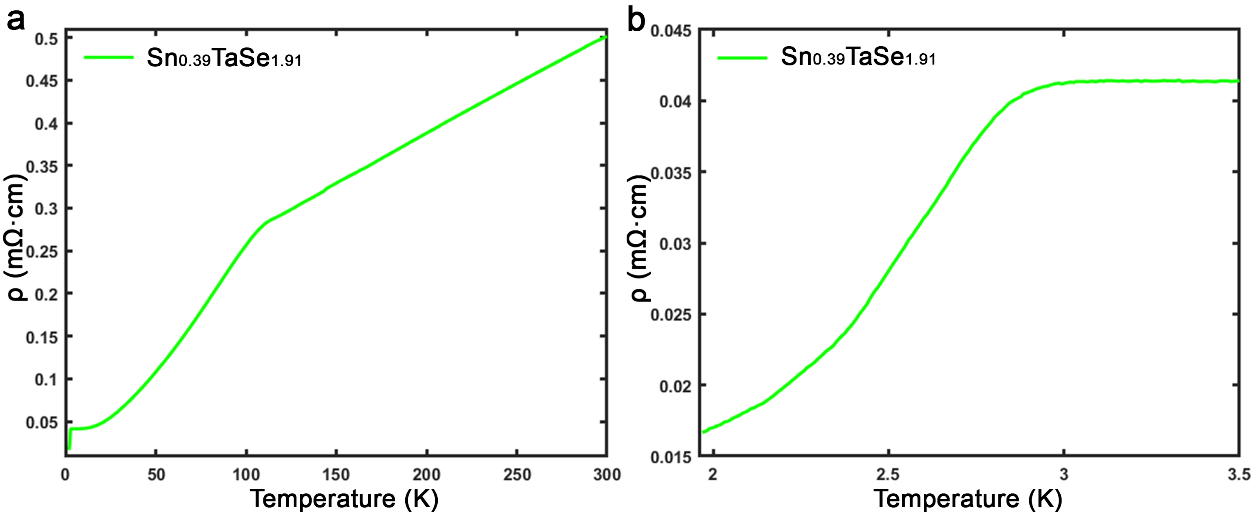

Supplementary Figure 43: Transport curve for $Sn_{0.39}TaSe_{1.91}$. (a) Full temperature range (0 to 300 K). (b) Low-temperature range (1.96 to 3.5 K).

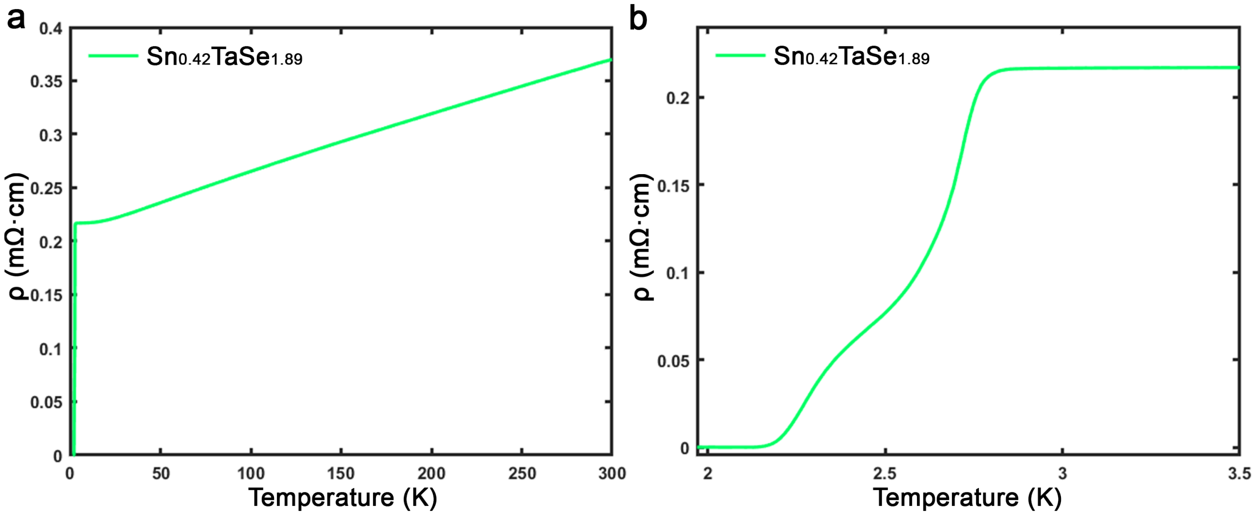

Supplementary Figure 44: Transport curve for $Sn_{0.42}TaSe_{1.89}$. (a) Full temperature range (0 to 300 K). (b) Low-temperature range (1.97 to 3.5 K).

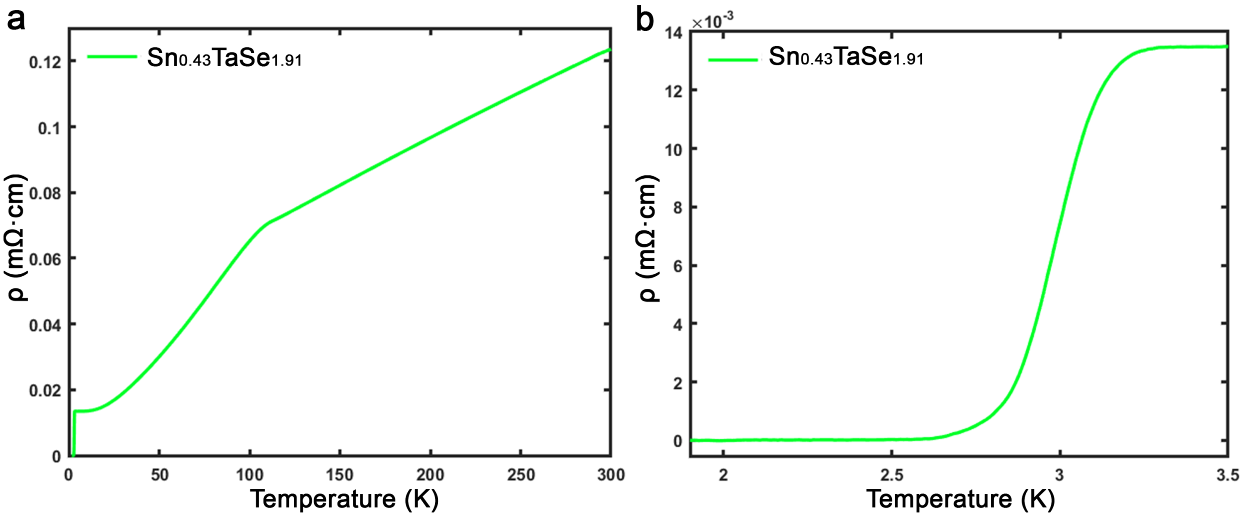

Supplementary Figure 45: Transport curve for $Sn_{0.43}TaSe_{1.91}$. (a) Full temperature range (0 to 300 K). (b) Low-temperature range (1.9 to 3.5 K).

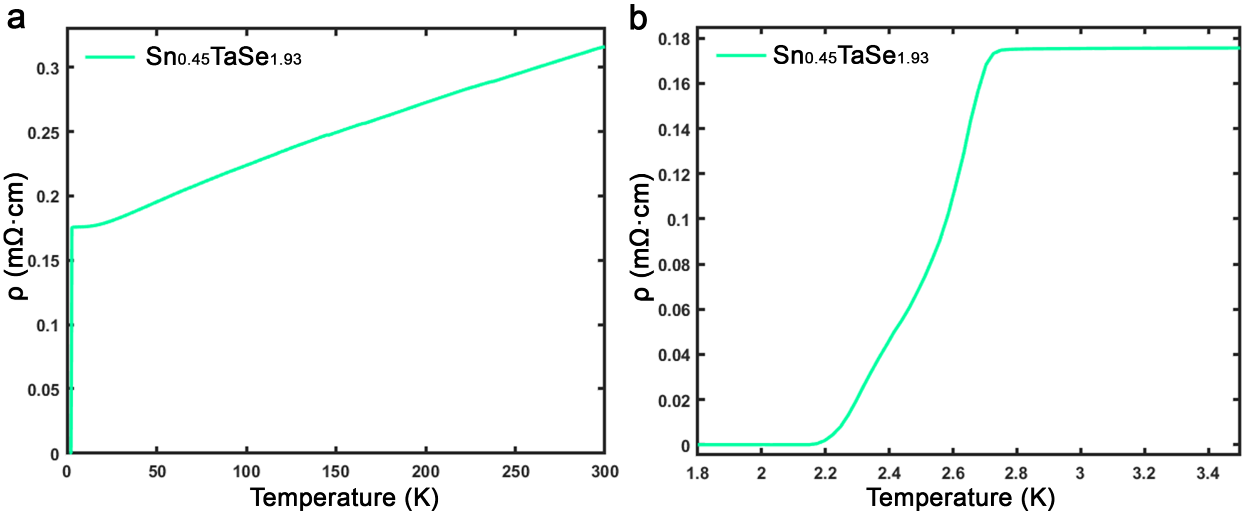

Supplementary Figure 46: Transport curve for $Sn_{0.45}TaSe_{1.93}$. (a) Full temperature range (0 to 300 K). (b) Low-temperature range (1.8 to 3.5 K).

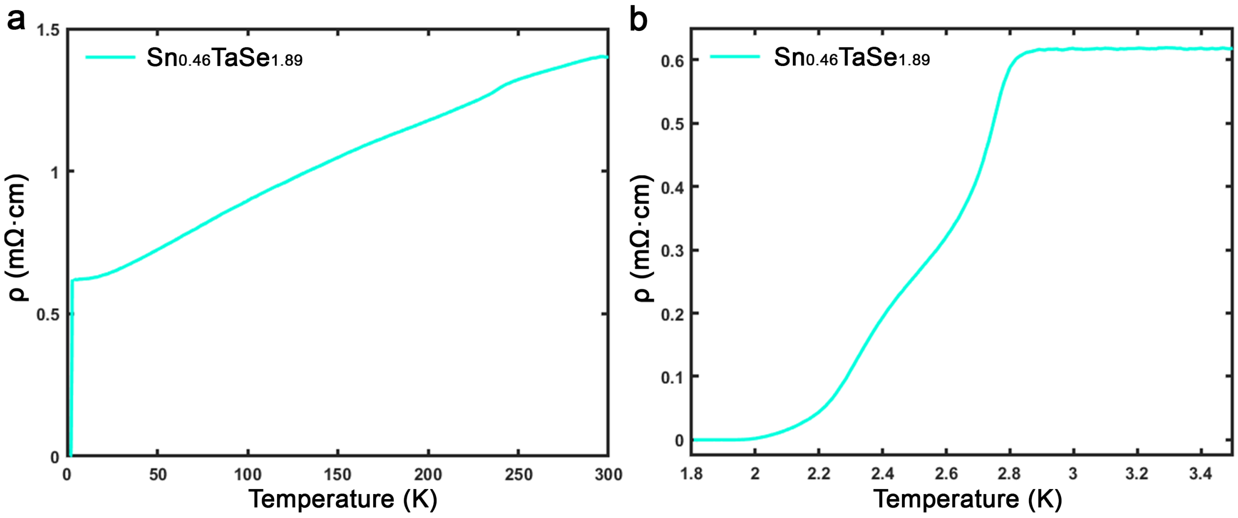

Supplementary Figure 47: Transport curve for $Sn_{0.46}TaSe_{1.89}$. (a) Full temperature range (0 to 300 K). (b) Low-temperature range (1.8 to 3.5 K).

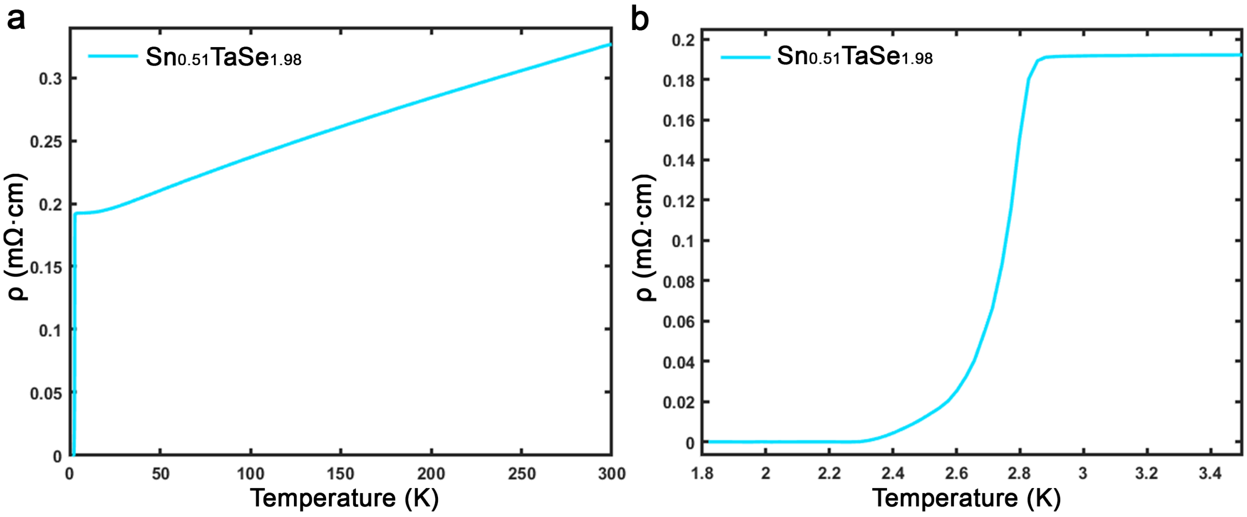

Supplementary Figure 48: Transport curve for $Sn_{0.51}TaSe_{1.98}$. (a) Full temperature range (0 to 300 K). (b) Low-temperature range (1.8 to 3.5 K).

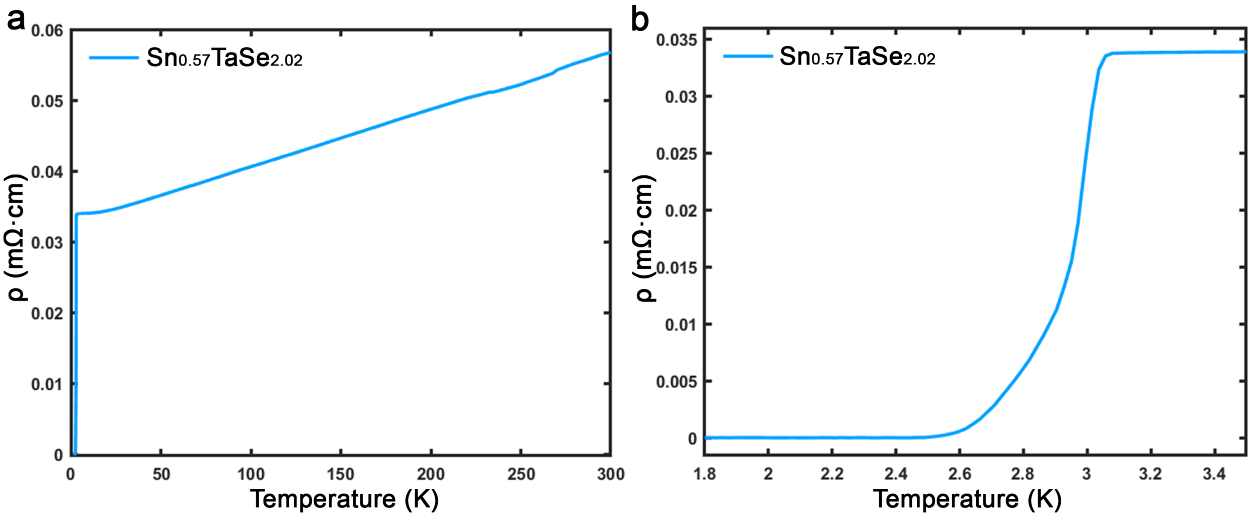

Supplementary Figure 49: Transport curve for $Sn_{0.57}TaSe_{2.02}$. (a) Full temperature range (0 to 300 K). (b) Low-temperature range (1.8 to 3.5 K).

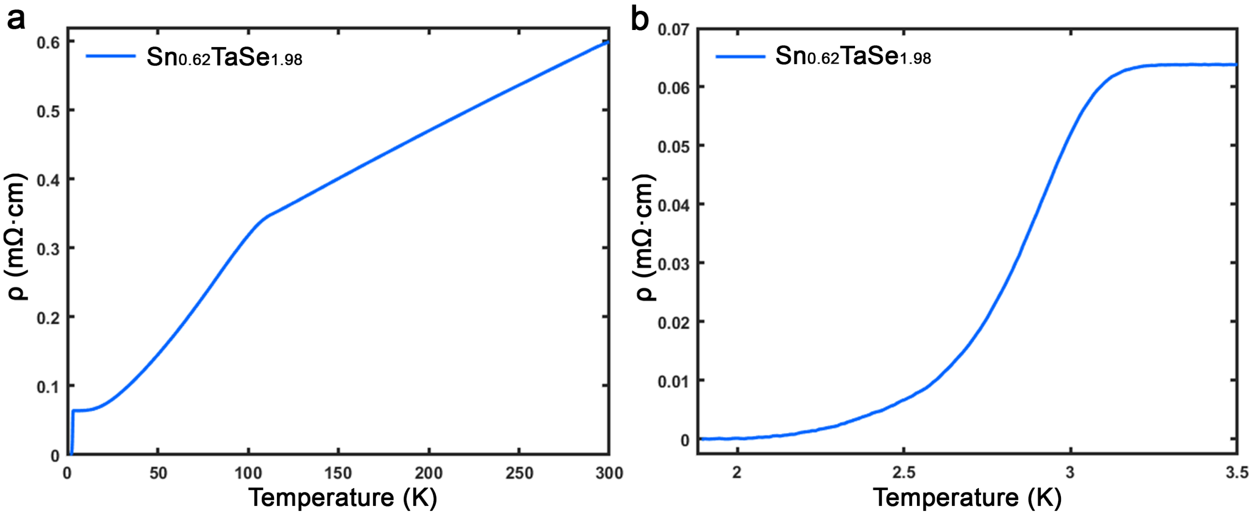

Supplementary Figure 50: Transport curve for $Sn_{0.62}TaSe_{1.98}$. (a) Full temperature range (0 to 300 K). (b) Low-temperature range (1.88 to 3.5 K).

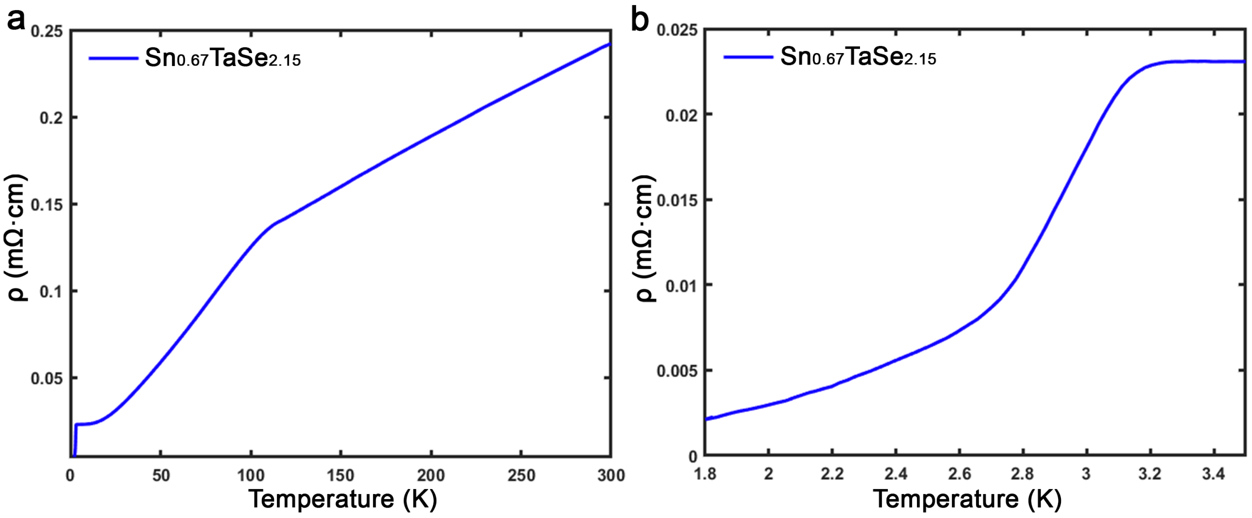

Supplementary Figure 51: Transport curve for $Sn_{0.67}TaSe_{2.15}$. (a) Full temperature range (0 to 300 K). (b) Low-temperature range (1.8 to 3.5 K).

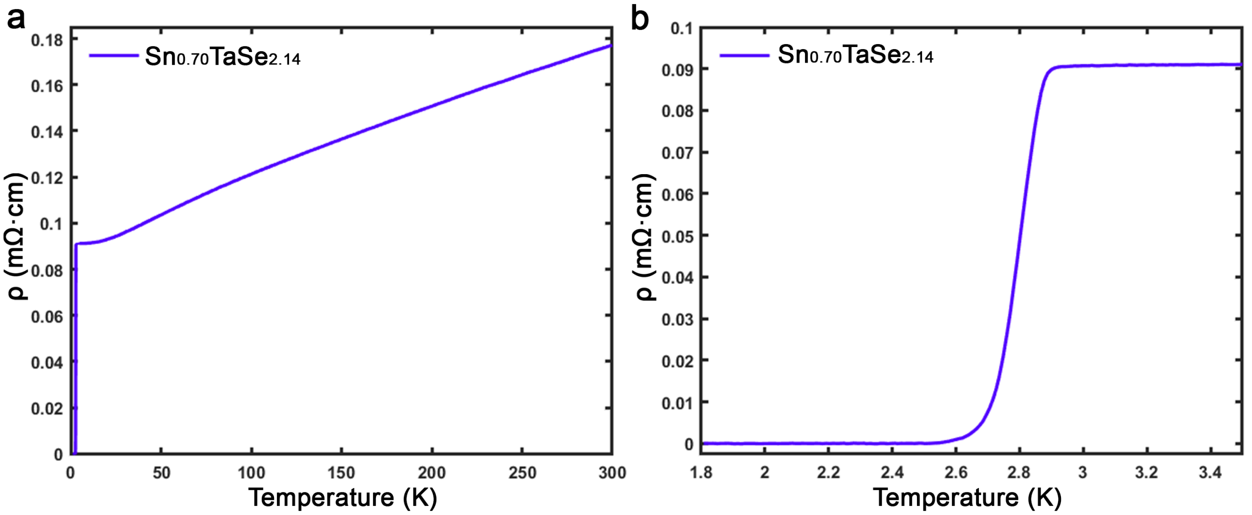

Supplementary Figure 52: Transport curve for $Sn_{0.70}TaSe_{2.14}$. (a) Full temperature range (0 to 300 K). (b) Low-temperature range (1.8 to 3.5 K).

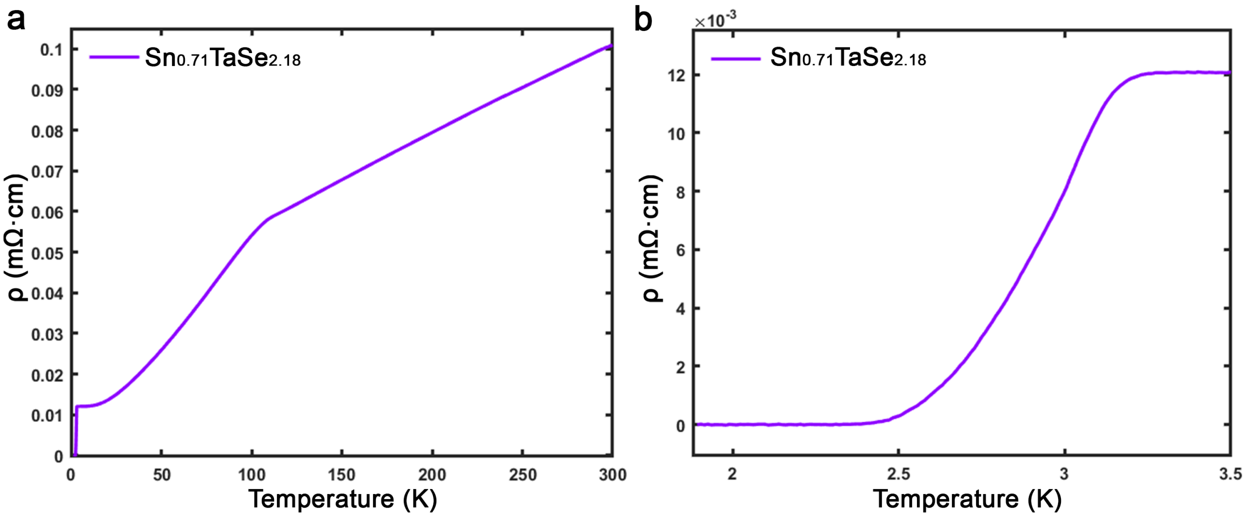

Supplementary Figure 53: Transport curve for $Sn_{0.71}TaSe_{2.18}$. (a) Full temperature range (0 to 300 K). (b) Low-temperature range (1.88 to 3.5 K).

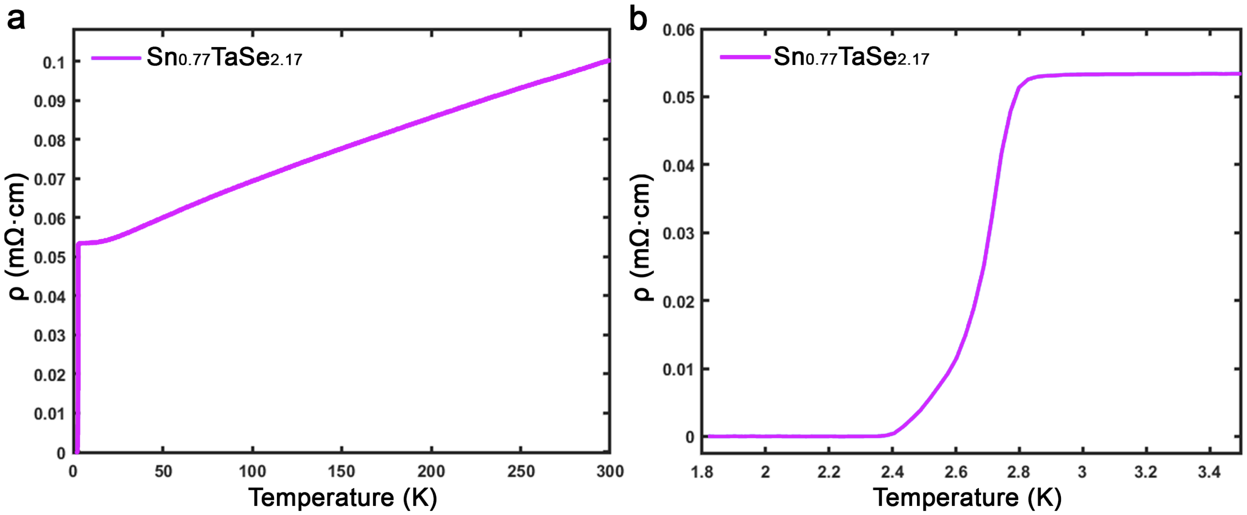

Supplementary Figure 54: Transport curve for $Sn_{0.77}TaSe_{2.17}$. (a) Full temperature range (0 to 300 K). (b) Low-temperature range (1.8 to 3.5 K).

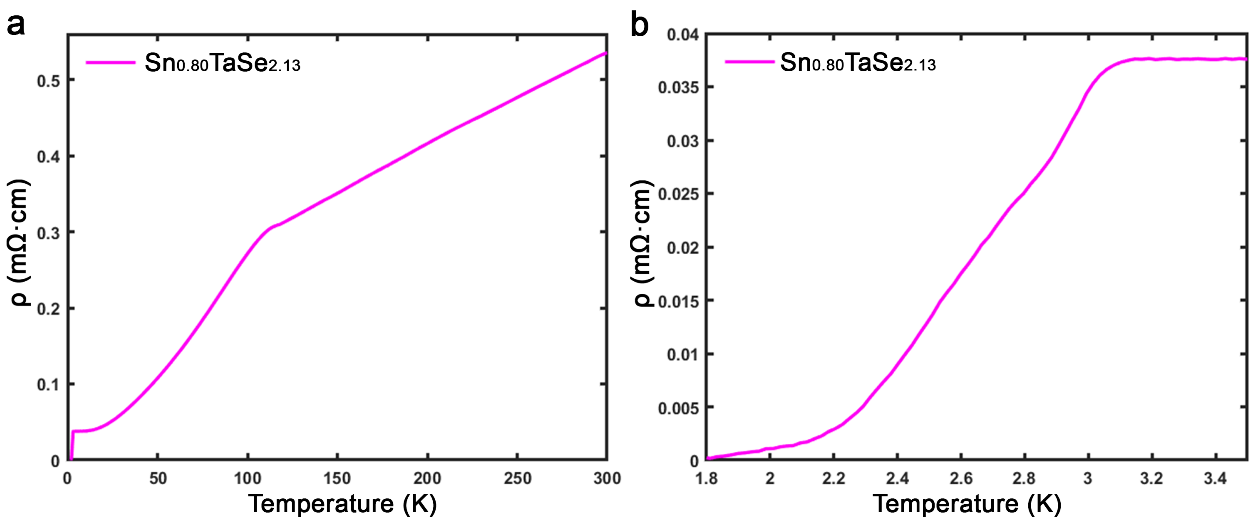

Supplementary Figure 55: Transport curve for $Sn_{0.80}TaSe_{2.13}$. (a) Full temperature range (0 to 300 K). (b) Low-temperature range (1.8 to 3.5 K).

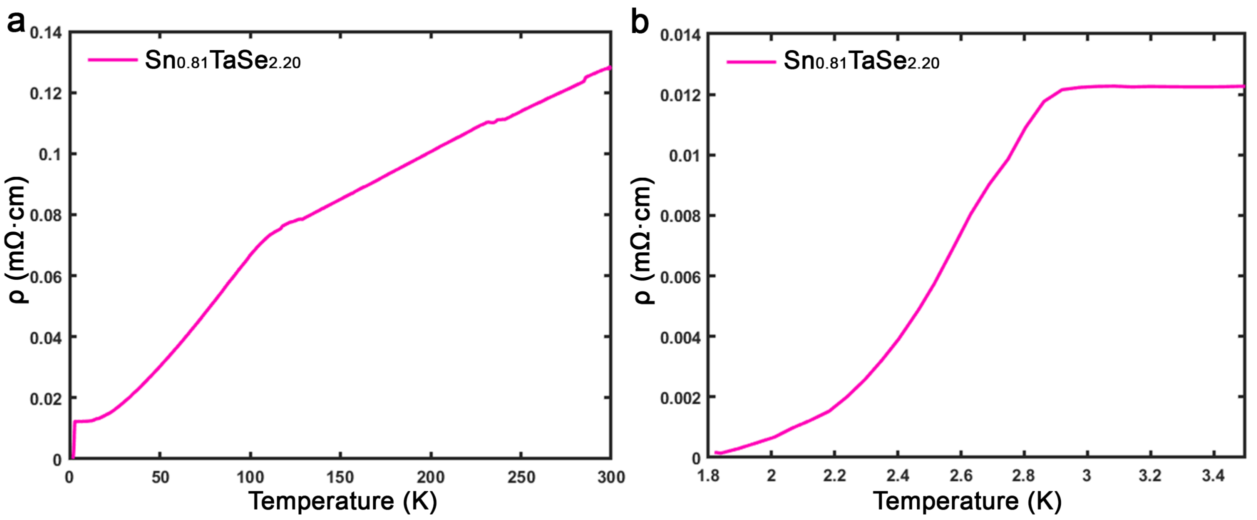

Supplementary Figure 56: Transport curve for $Sn_{0.81}TaSe_{2.20}$. (a) Full temperature range (0 to 300 K). (b) Low-temperature range (1.8 to 3.5 K).

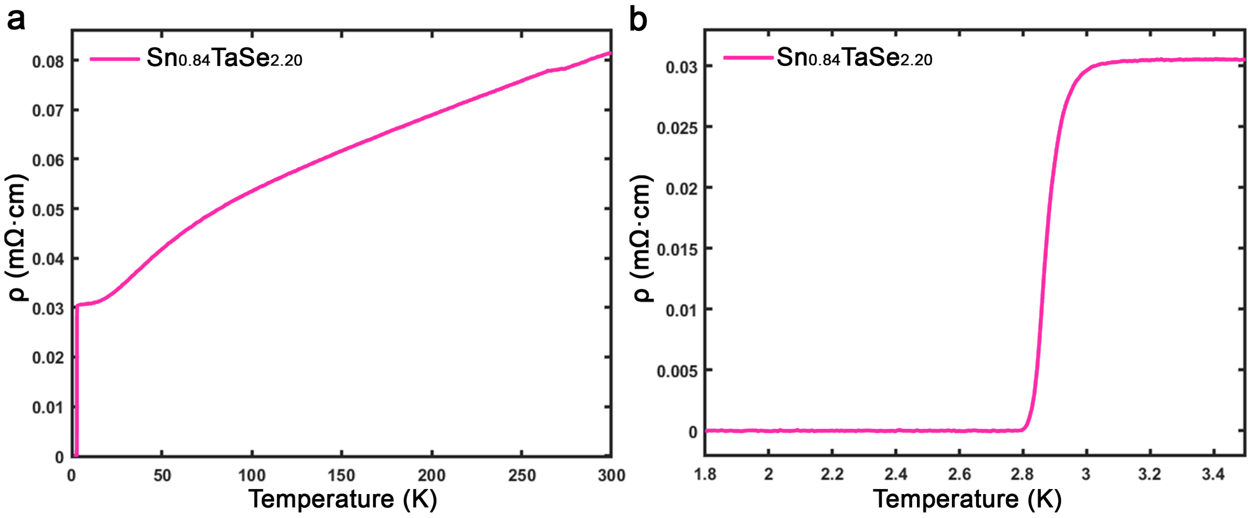

Supplementary Figure 57: Transport curve for $Sn_{0.84}TaSe_{2.20}$. (a) Full temperature range (0 to 300 K). (b) Low-temperature range (1.8 to 3.5 K).

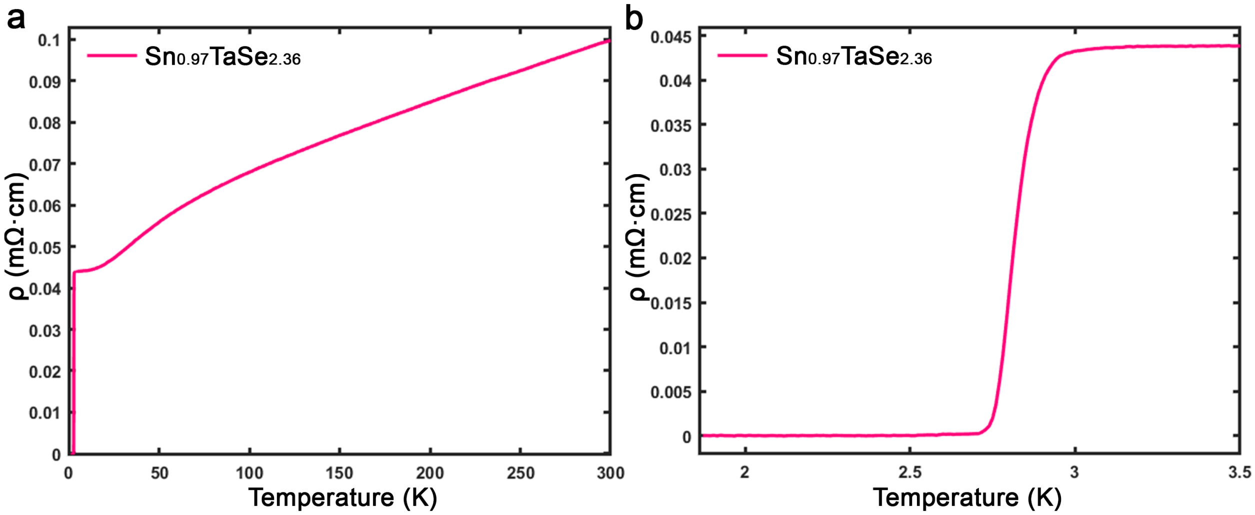

Supplementary Figure 58: Transport curve for $Sn_{0.97}TaSe_{2.36}$. (a) Full temperature range (0 to 300 K). (b) Low-temperature range (1.86 to 3.5 K).

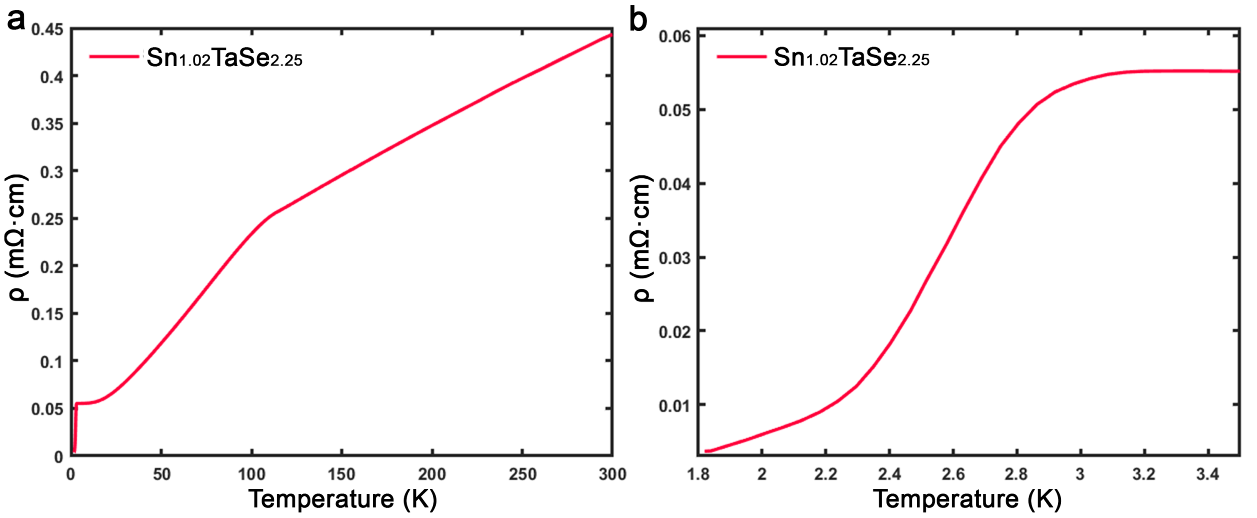

Supplementary Figure 59: Transport curve for $Sn_{1.02}TaSe_{2.25}$. (a) Full temperature range (0 to 300 K). (b) Low-temperature range (1.80 to 3.5 K).

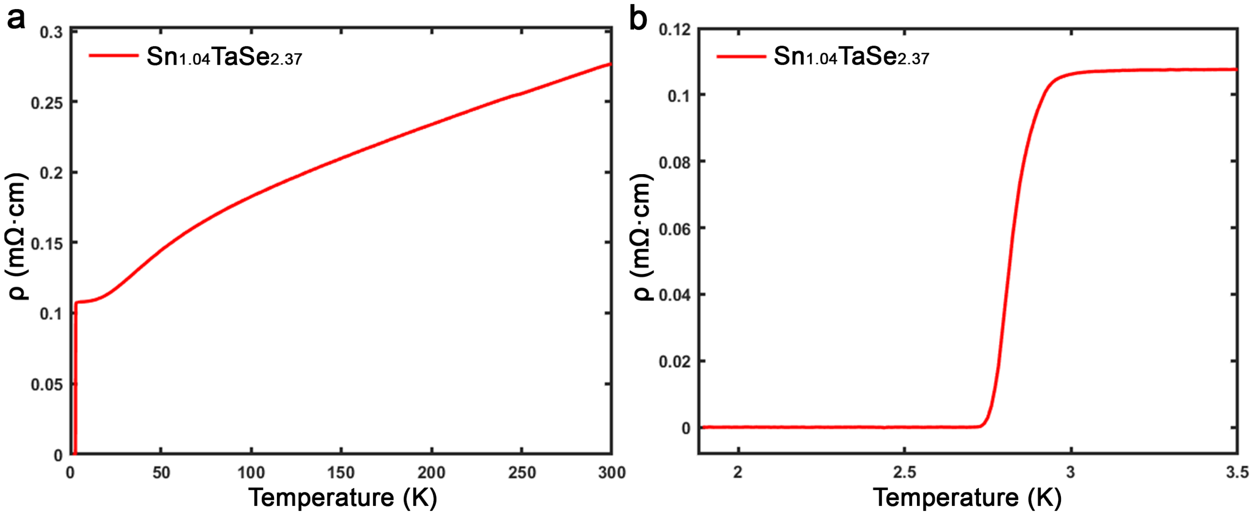

Supplementary Figure 60: Transport curve for $Sn_{1.04}TaSe_{2.37}$. (a) Full temperature range (0 to 300 K). (b) Low-temperature range (1.88 to 3.5 K).

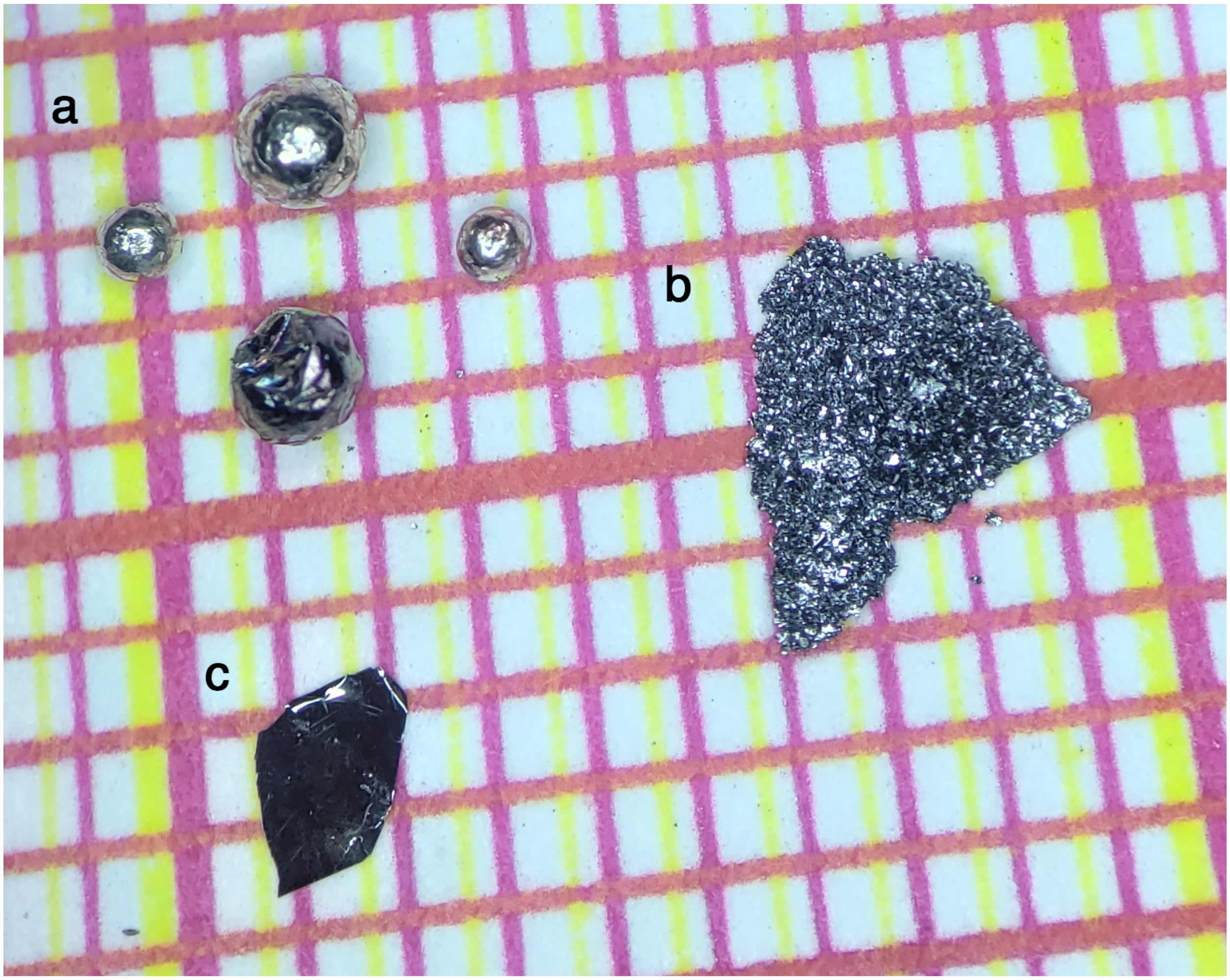

Supplementary Figure 61: Typical products of CVT Sn_xTaSe_2 growth: a) unreacted Sn, b) polycrystalline Sn_xTaSe_2, and c) Sn_xTaSe_2 flake.

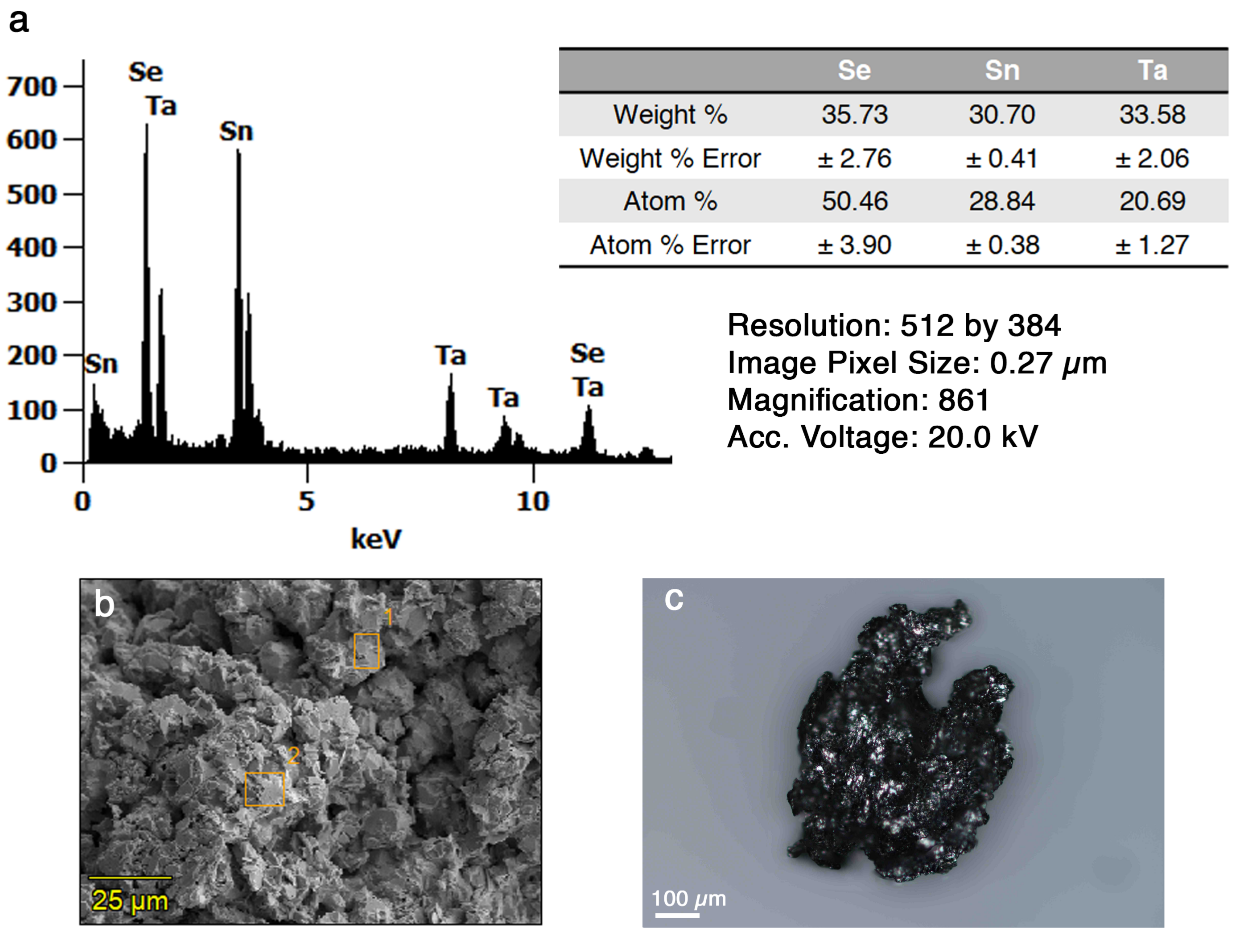

	Se	Sn	Ta
Weight %	35.73	30.70	33.58
Weight % Error	± 2.76	± 0.41	± 2.06
Atom %	50.46	28.84	20.69
Atom % Error	± 3.90	± 0.38	± 1.27

Supplementary Figure 62: Polycrystalline Sn_xTaSe_2 products obtained from flux growth attempts. (a) EDS area collection results for rectangle one. (b) SEM image of crystal and spot used for EDS collection. Both areas had similar results. (c) optical microscope image of a representative Sn_xTaSe_2 cluster (image is layered from different focuses).

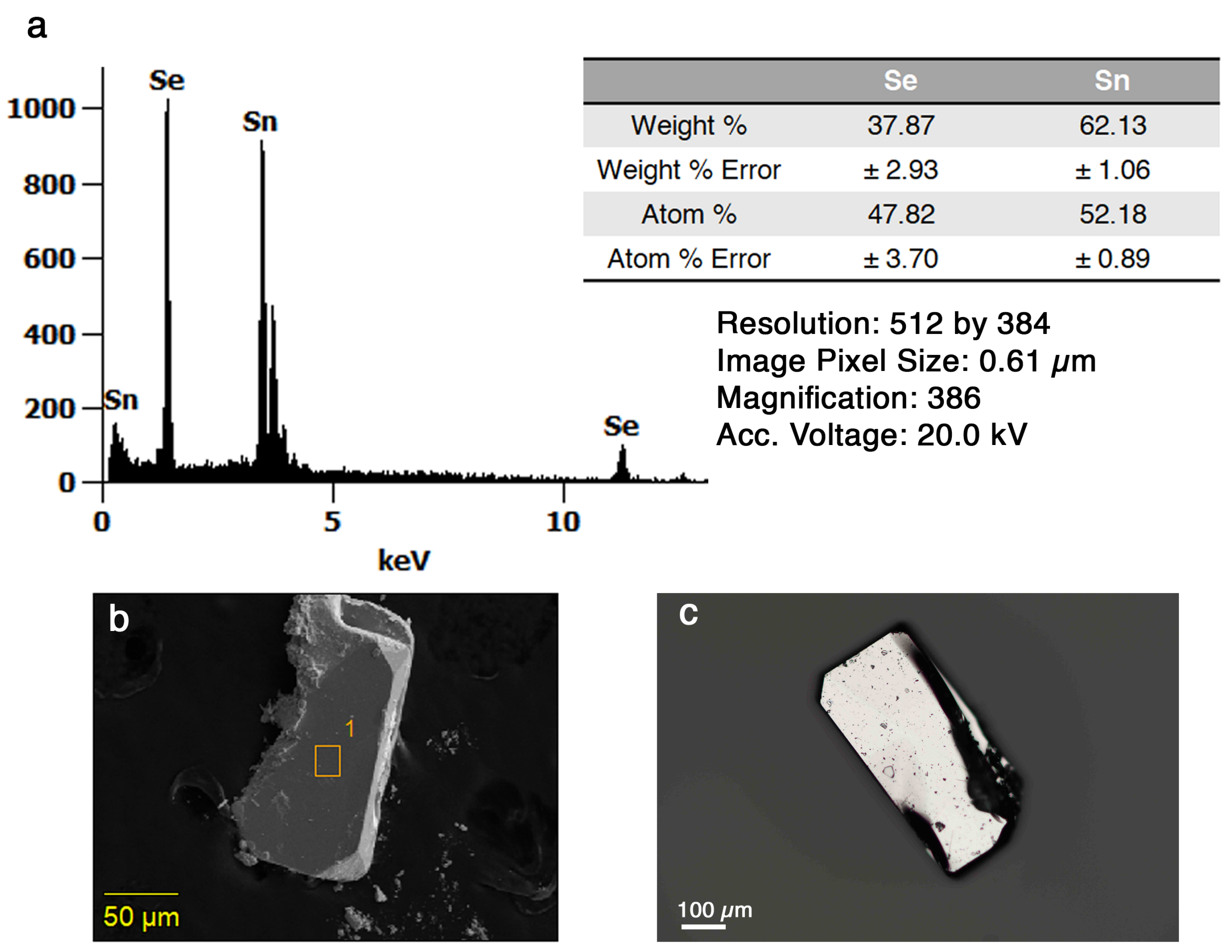

	Se	Sn
Weight %	37.87	62.13
Weight % Error	± 2.93	± 1.06
Atom %	47.82	52.18
Atom % Error	± 3.70	± 0.89

Supplementary Figure 63: SnSe products obtained from flux growth attempts. (a) EDS area collection results. (b) SEM image of crystal and spot used for EDS collection. (c) optical microscope image of a representative SnSe crystal.

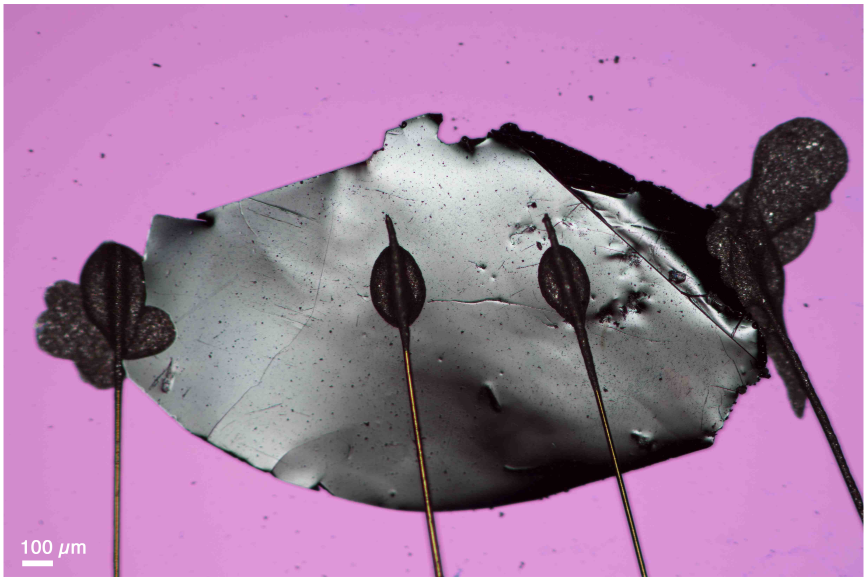

Supplementary Figure 64: Example of sample preparation for temperature dependent electronic transport measurements.

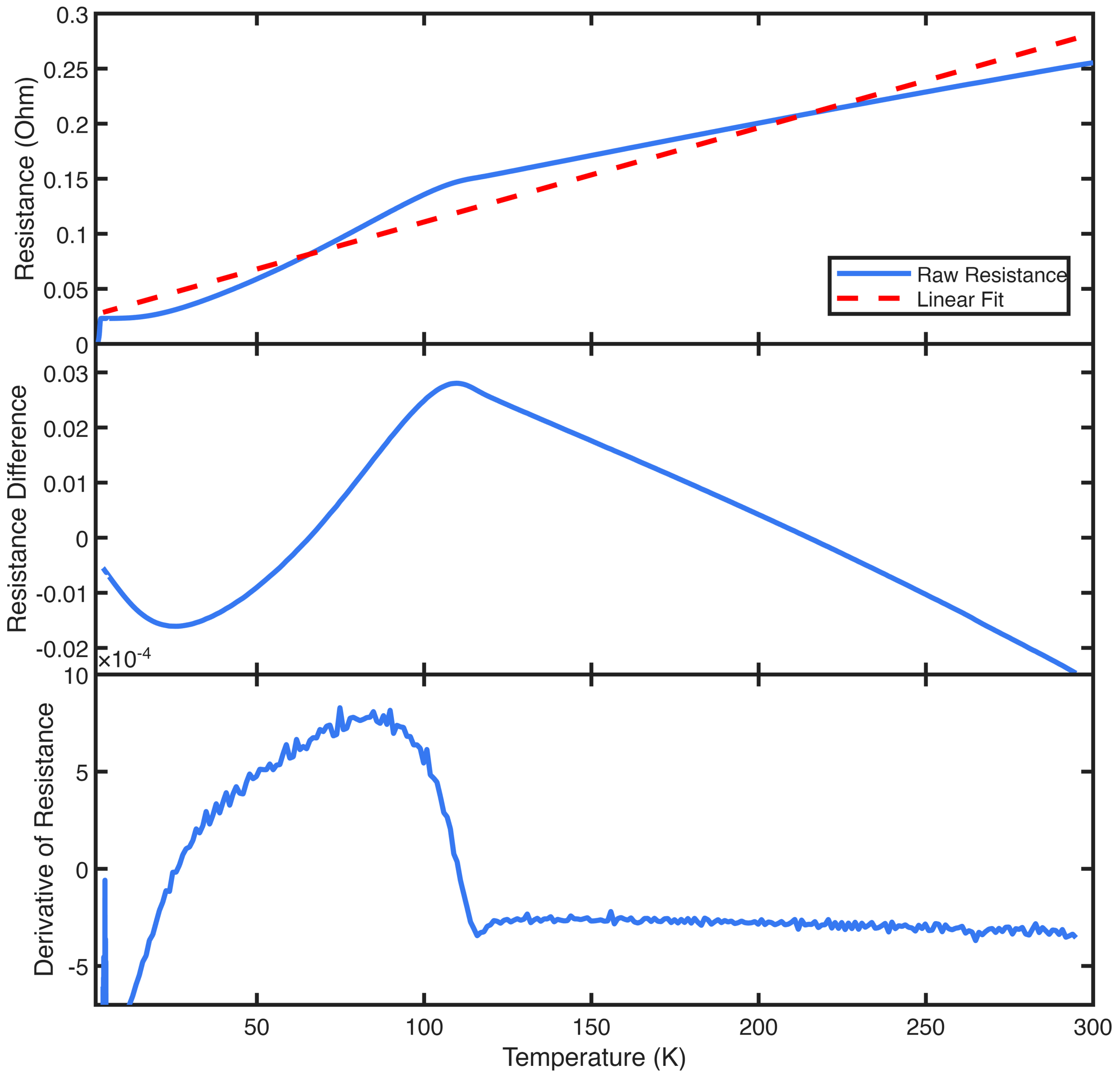

Supplementary Figure 65: Raw resistance data with linear fit (using a fit range of 4 to 295 K), difference between raw resistance and linear fit, and derivative of the difference for $Sn_{0.27}TaSe_{1.80}$, representing a sample with a visible CDW.

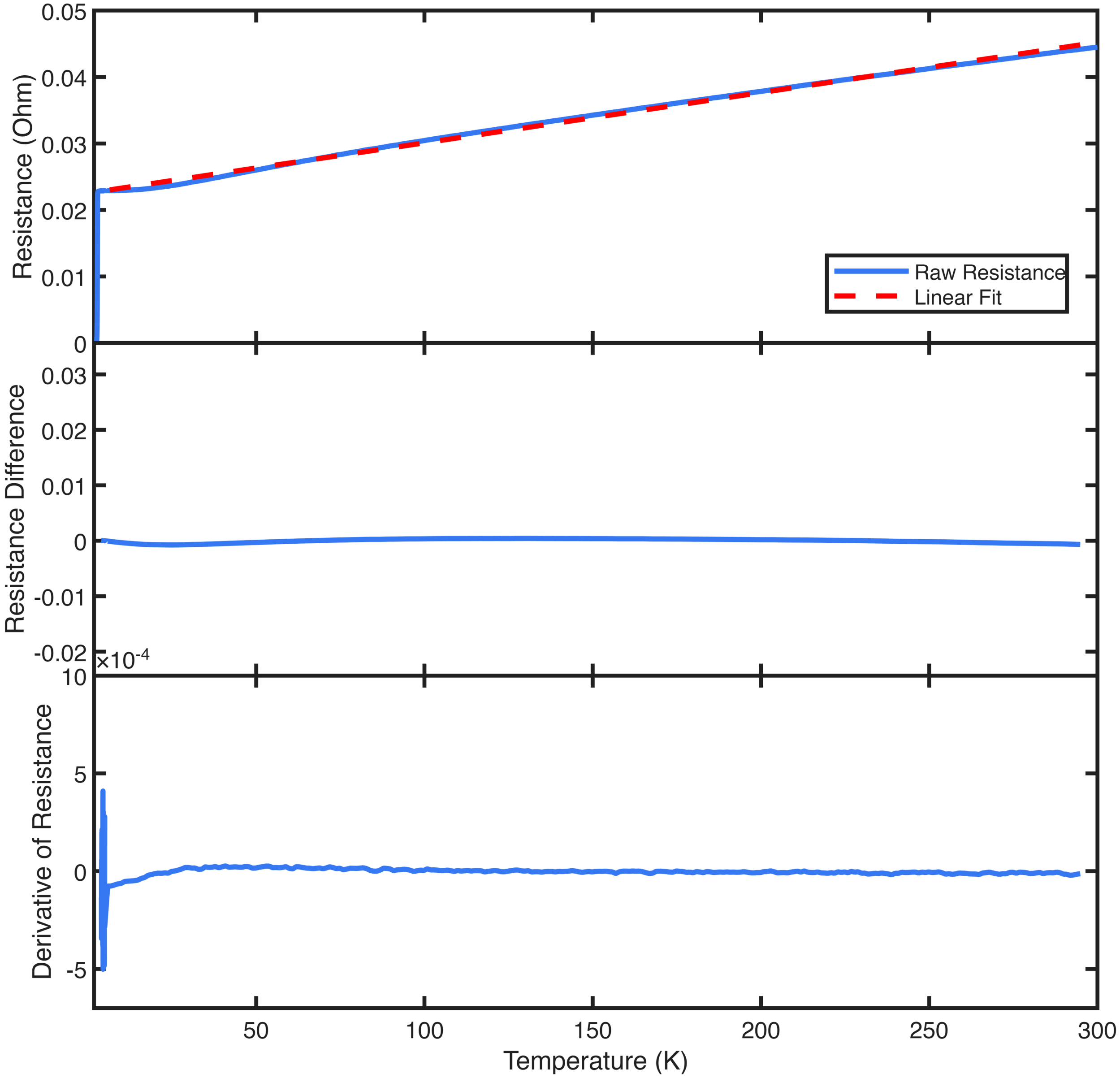

Supplementary Figure 66: Raw resistance data with linear fit (using a fit range of 4 to 295 K), difference between raw resistance and linear fit, and derivative of the difference for $Sn_{0.70}TaSe_{2.14}$, representing a sample without a visible CDW.

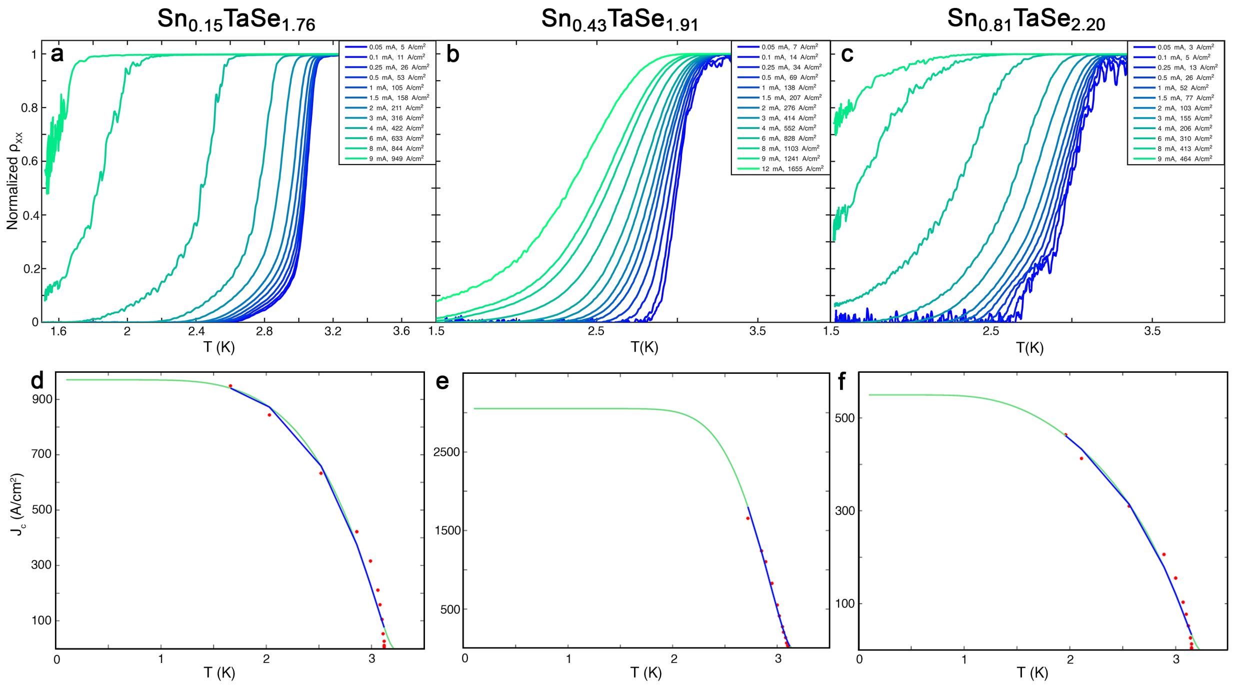

Supplementary Figure 67: (a-c) Superconducting transition across a range of currents and (d-e) critical current density as a function of temperature for $Sn_{0.15}TaSe_{1.76}$, $Sn_{0.43}TaSe_{1.91}$, and $Sn_{0.81}TaSe_{2.20}$ respectively.

The fits in d-f use the Matlab code written by Talantsev et al. for type II, S-wave, single-band, 3D rectangular superconductors, with the following equations:[7]

$$J_c(T, sf) = \frac{\phi_0(\ln\kappa+0.5)}{4\pi\mu_0}\frac{1}{\lambda_0^2}\rho\left(\frac{1}{a}\tanh\left(\frac{a\rho^{1/2}}{\lambda_0}\right)+\frac{1}{b}\tanh\left(\frac{b\rho^{1/2}}{\lambda_0}\right)\right),$$
$$\rho = 1-\frac{1}{2k_BT}\int_0^\infty \cosh^{-2}\left(\frac{\sqrt{\epsilon^2+\Delta^2(T)}}{2k_BT}\right)d\epsilon$$

Below are extracted $J_c(0)$ for the three samples, compared to reports for similar compounds. Fitted values may be unreliable but provide an estimate based on the available data.

**Material**	J_c **(A/cm^2)**	T/T_c	**Reference**
$Sn_{0.15}TaSe_{1.76}$	$\sim$ 970	0.03	This work
$Sn_{0.43}TaSe_{1.91}$	$\sim$ 3050	0.03	This work
$Sn_{0.81}TaSe_{2.20}$	$\sim$ 550	0.03	This work
$2H$-TaS_2	$\sim$ 700	$\sim$ 0.05	58
$NbSe_2$	138,997	0.32	59
Sliding $3R$-$NbSe_2$	200,000	0.53	60
$Fe_{0.0011}NbSe_2$	357,280	0.38	59
$Cr_{0.0005}NbSe_2$	391,884	0.30	61
$(InSe_2)_{0.12}NbSe_2$	820,000	0.17	62
$Cu_{0.058}TiSe_2$	$\sim$ 35	0.65	63

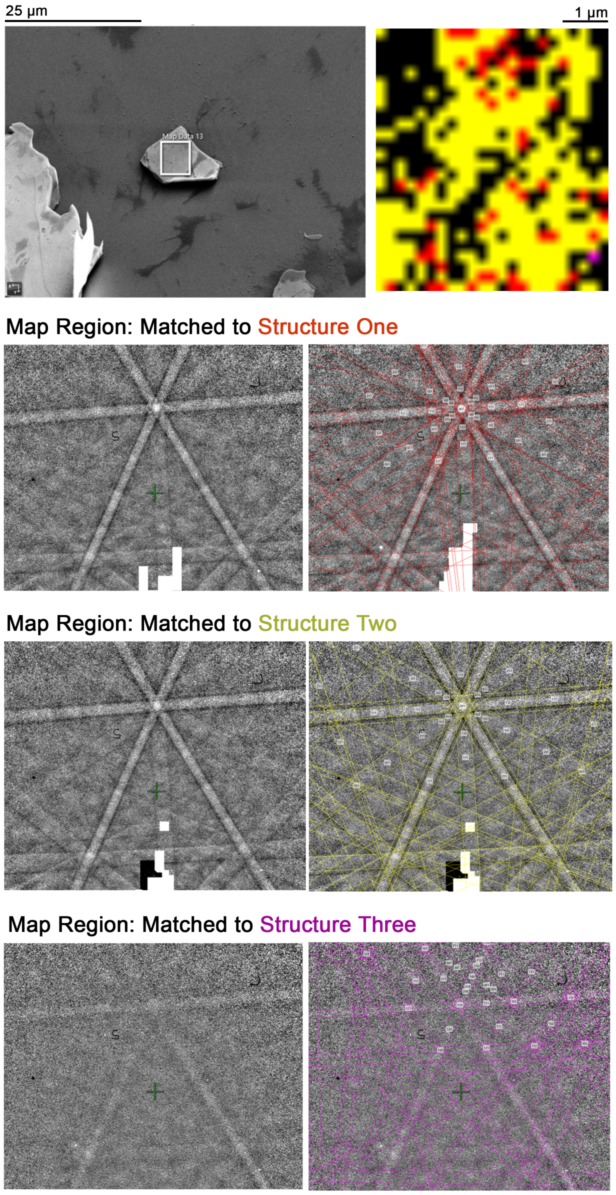

Supplementary Figure 68: EBSD Map and select Kikuchi patterns from a Sn_xTaSe_2 sample exfoliated onto SiO_2. Regions are chosen that show a match to each of the three structures. Dark areas on the map appear to be regions where the pattern was too weak to index, rather than belonging to a non-matching phase.

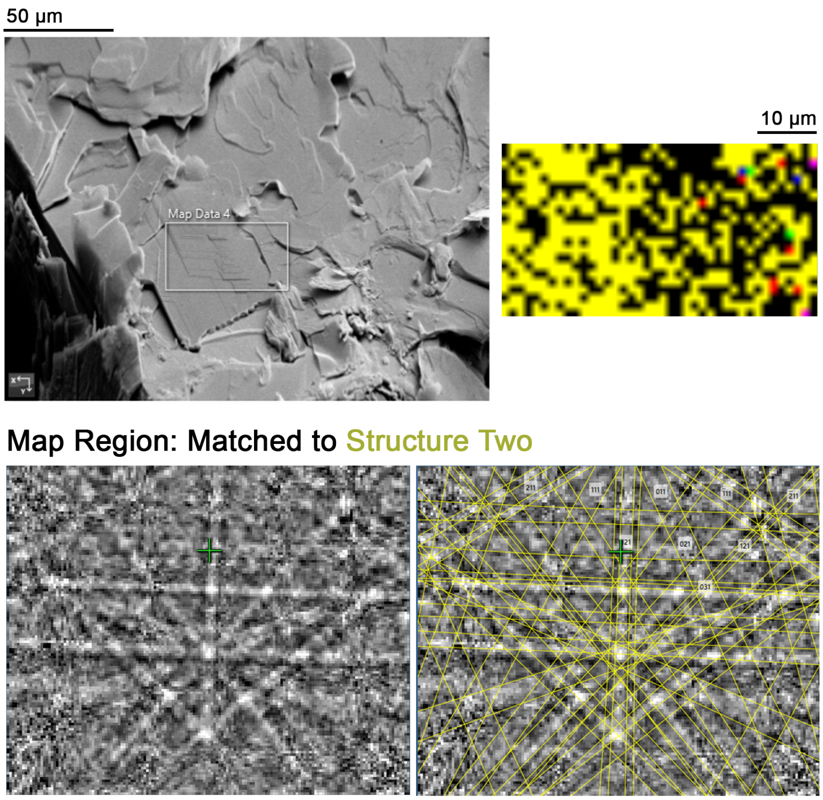

Supplementary Figure 69: EBSD Map and Kikuchi pattern for sample corresponding to structure two. Sample for STEM data collection was extracted from the boxed region. Dark areas on the map appear to be regions where the pattern was too weak to index, rather than belonging to a non-matching phase.

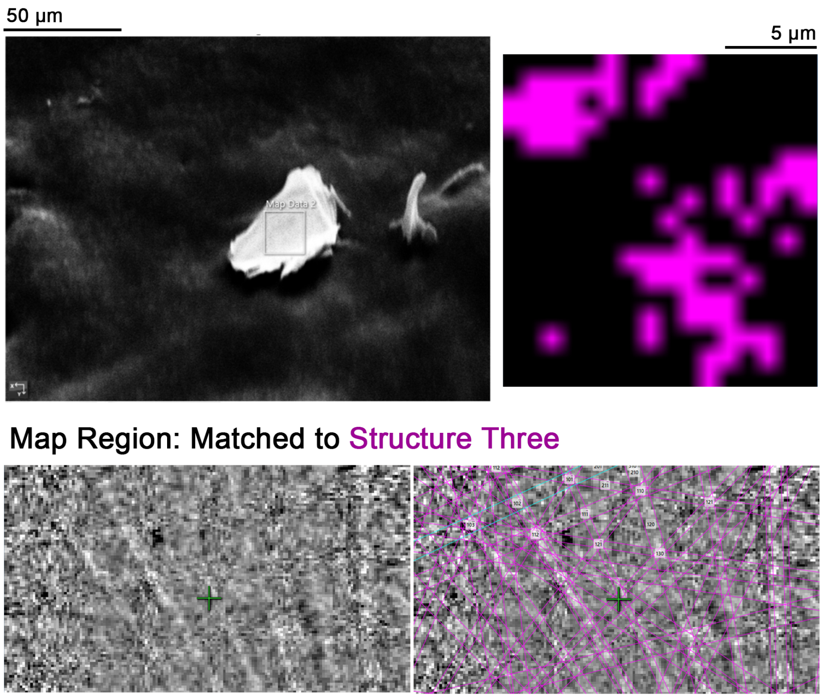

Supplementary Figure 70: EBSD Map and Kikuchi pattern corresponding to structure three. Sample for STEM data collection was extracted from this sample. Dark areas on the map appear to be regions where the pattern was too weak to index, rather than belonging to a non-matching phase.

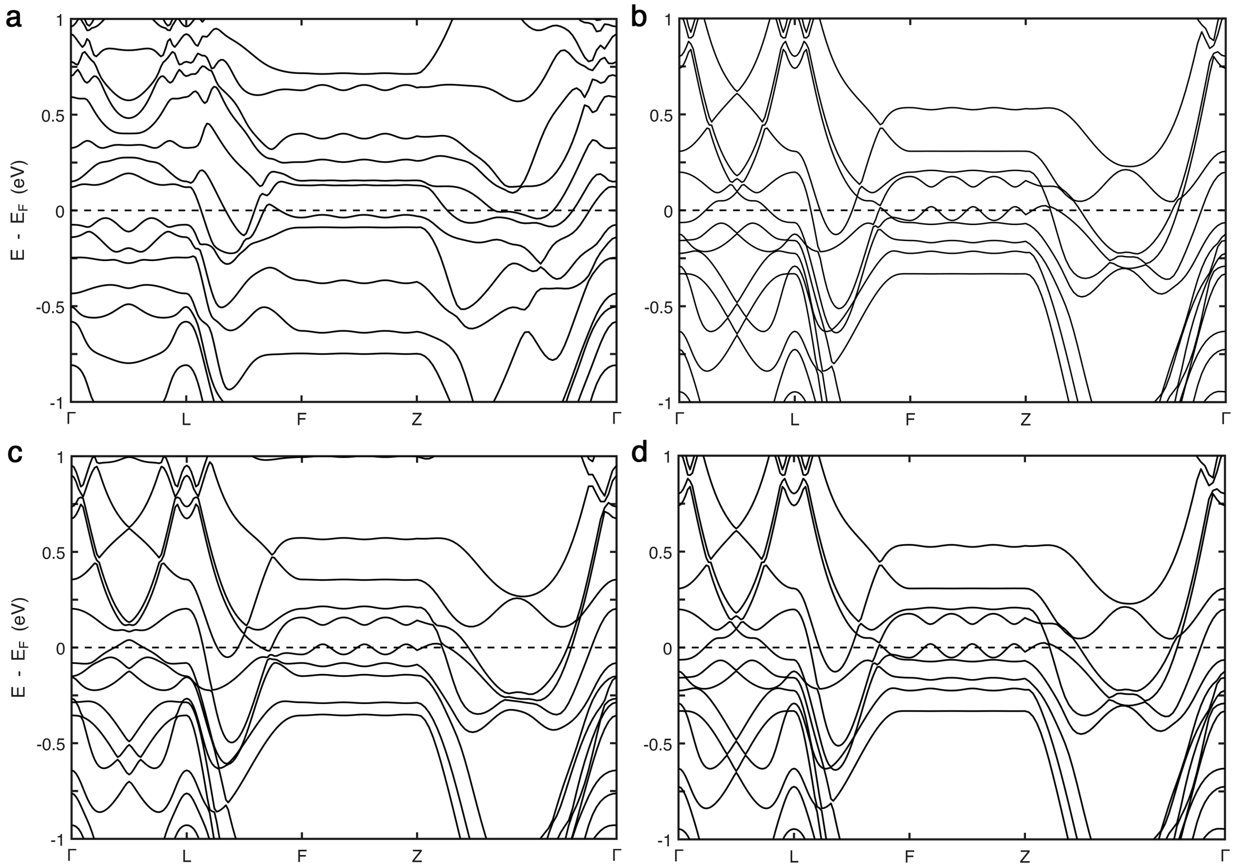

Supplementary Figure 71: Band structure for structure one: (a) modeled as $Sn_{1/6}TaSe_2$ with atomic parameters directly from cif and (b) optimized parameters or, (c) modeled without Sn using atomic parameters directly from cif and (d) optimized parameters.

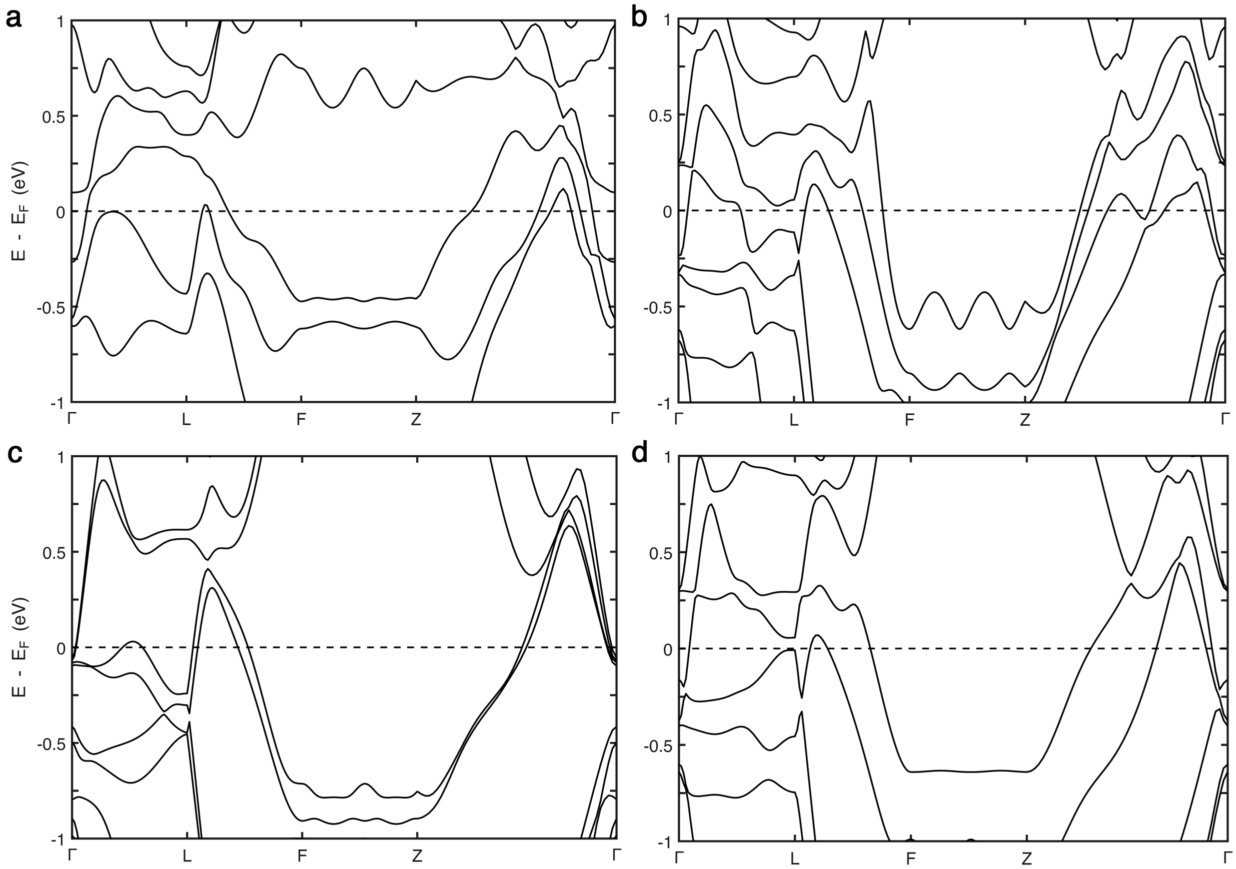

Supplementary Figure 72: Band structure for structure two: (a) modeled as $Sn_{1/4}TaSe_2$ with atomic parameters directly from cif and (b) optimized parameters, or (c) modeled without Sn using atomic parameters directly from cif and (d) optimized parameters.

Supplementary Tables

Supplementary Table 1: Stoichiometries, transition temperature, and residual-resistant ratios for measured flakes.

**Stoichiometry**	**EDS At. % (Sn, Ta, Se)**	**Transport Agent**	**RRR**	$T_{\mathbf{CDW}}$**(K)**	$T_{c_{90\%}}$**(K)**
$Sn_{0.10(4)}TaSe_{1.66(5)}$	4(1), 36(1), 60.0(7)	I_2	1.51		2.87
$Sn_{0.10(2)}TaSe_{1.76(2)}$	3.3(7), 35.1(4), 61.6(5)	I_2	10.47	109.9	3.10
$Sn_{0.15(6)}TaSe_{1.76(7)}$	5(2), 34(1), 60.4(9)	I_2	1.68		3.09
$Sn_{0.19(2)}TaSe_{1.68(3)}$	6.6(7), 34.9(5), 58.5(3)	I_2	15.56	111.4	3.04
$Sn_{0.27(6)}TaSe_{1.80(8)}$	9(2), 32(1), 59(2)	I_2	11.10	109.8	3.18
$Sn_{0.29(3)}TaSe_{1.82(3)}$	9.4(9), 32.2(5), 58.4(5)	I_2	7.2	110.6	3.16
$Sn_{0.32(5)}TaSe_{1.83(7)}$	10.(2), 32(1), 58.0(6)	I_2	13.24	110.7	2.80
$Sn_{0.33(6)}TaSe_{1.96(6)}$	10.(2), 30.(1), 59.5(8)	I_2	18.08	112.5	3.17
$Sn_{0.39(9)}TaSe_{1.91(9)}$	12(3), 30.(1), 58(1)	I_2	12.48	111.0	2.75
$Sn_{0.42(6)}TaSe_{1.89(7)}$	13(2), 30.(1), 57.1(7)	I_2	1.71		2.75
$Sn_{0.43(3)}TaSe_{1.91(2)}$	12.7(5), 29.9(4), 57.3(3)	I_2	9.29	109.2	3.12
$Sn_{0.45(5)}TaSe_{1.93(6)}$	13(1), 30.(1), 57.1(5)	I_2	1.80		2.69
$Sn_{0.46(7)}TaSe_{1.89(8)}$	14(2), 30.(1), 56(1)	I_2	2.27		2.78
$Sn_{0.51(1)}TaSe_{1.98(2)}$	14.7(2), 28.6(3), 56.7(4)	I_2	1.71		2.81
$Sn_{0.57(1)}TaSe_{2.02(1)}$	16.0(3), 27.9(2), 56.2(2)	I_2	1.68		3.02
$Sn_{0.62(5)}TaSe_{1.98(6)}$	17(1), 27.8(9), 54.9(5)	I_2	9.40	109.2	3.05
$Sn_{0.67(8)}TaSe_{2.15(9)}$	17(2), 26(1), 56.3(7)	I_2	10.50	111.4	3.07
$Sn_{0.70(9)}TaSe_{2.14(10)}$	18(2), 26(1), 57(1)	I_2	1.95		2.86
$Sn_{0.71(6)}TaSe_{2.18(6)}$	18(1), 25.7(8), 56.0(6)	I_2	8.38	108.6	3.11
$Sn_{0.77(8)}TaSe_{2.17(9)}$	20.(2), 25(1), 55.1(7)	I_2	1.91		2.77
$Sn_{0.80(9)}TaSe_{2.13(7)}$	20.(2), 25.4(9), 54(1)	I_2	14.24	111.9	3.05
$Sn_{0.81(9)}TaSe_{2.20(9)}$	20.(2), 25(1), 55(1)	I_2	10.49	111.2	2.81
$Sn_{0.84(9)}TaSe_{2.20(9)}$	21(2), 25(1), 54.5(7)	$SeCl_4$	2.67		2.94
$Sn_{0.97(3)}TaSe_{2.36(3)}$	22.4(7), 23.1(4), 54.5(3)	$SeCl_4$	2.29		2.90
$Sn_{1.02(9)}TaSe_{2.25(9)}$	24(2), 23(1), 52.7(5)	I_2	8.04	110.9	2.84
$Sn_{1.04(4)}TaSe_{2.37(5)}$	23.5(8), 22.7(6), 53.7(4)	$SeCl_4$	2.57		2.91

Supplementary Table 2: Atomic parameters with fractional atomic coordinates and equivalent isotropic displacement parameters (Å$^2\times10^3$) for structure one, datset one ($Sn_{0.18(3)}Ta_{1.00(9)}Se_{2.0(1)}$). U_{eq} is defined as 1/3 of the trace of the orthogonalised U_{IJ} tensor. Atomic positions are fixed in final refinement; provided uncertainties for atomic coordinates are determined when the occupancies are refined.

**Atom**	**Wyckoff**	**Site**	**Occupancy**	**x**	**y**	**z**	$\mathbf{U_{eq}}$
Ta1	3*a*	3*m*	0.98(9)	1/3	2/3	0.50001(1)	7.4(4)
Se1	3*a*	3*m*	1.0(1)	2/3	1/3	0.4116(2)	8.3(13)
Se2	3*a*	3*m*	0.98(10)	2/3	1/3	0.5881(2)	11.7(13)
Sn1	3*a*	3*m*	0.18(3)	1	1	0.6514(9)	15(6)

Supplementary Table 3: Anisotropic displacement parameters (Å$^2\times10^3$) for structure one, dataset one ($Sn_{0.18(3)}Ta_{1.00(9)}Se_{2.0(1)}$). The anisotropic displacement factor exponent takes the form: $-2\pi^2[h^2a^{*2}U_{11}+2hka^*b^*U_{12}+\ldots]$.

**Atom**	$\mathbf{U_{11}}$	$\mathbf{U_{22}}$	$\mathbf{U_{33}}$	$\mathbf{U_{23}}$	$\mathbf{U_{13}}$	$\mathbf{U_{12}}$
Ta1	5.7(4)	5.7(4)	10.7(6)	0	0	2.9(2)
Se1	6.5(14)	6.5(14)	12(2)	0	0	3.3(7)
Se2	11.5(15)	11.5(15)	12(2)	0	0	5.8(7)
Sn1	15(6)	15(6)	14(9)	0	0	7(3)

Supplementary Table 4: Atomic parameters with fractional atomic coordinates and equivalent isotropic displacement parameters ($Å^2 \times 10^3$) for structure one, dataset two ($Sn_{0.08(2)}Ta_{1.00(5)}Se_{1.96(7)}$). U_{eq} is defined as 1/3 of the trace of the orthogonalised U_{IJ} tensor. Atomic positions are fixed in final refinement; provided uncertainties for atomic coordinates are determined when the occupancies are refined.

**Atom**	**Wyckoff**	**Site**	**Occupancy**	**x**	**y**	**z**	$\mathbf{U_{eq}}$
Ta1	3*a*	3*m*	1.00(5)	1/3	2/3	0.37551(4)	8.5(2)
Se1	3*a*	3*m*	0.98(5)	2/3	1/3	0.46323(11)	10.0(8)
Se2	3*a*	3*m*	0.99(5)	2/3	1/3	0.28762(11)	8.2(7)
Sn1	3*a*	3*m*	0.076(9)	1	1	0.5252(10)	3(6)

Supplementary Table 5: Anisotropic displacement parameters ($Å^2 \times 10^3$) for structure one, dataset two ($Sn_{0.08(2)}Ta_{1.00(5)}Se_{1.96(7)}$). The anisotropic displacement factor exponent takes the form: $-2\pi^2[h^2a^{*2}U_{11}+2hka^*b^*U_{12}+\ldots]$.

**Atom**	$\mathbf{U_{11}}$	$\mathbf{U_{22}}$	$\mathbf{U_{33}}$	$\mathbf{U_{23}}$	$\mathbf{U_{13}}$	$\mathbf{U_{12}}$
Ta1	7.2(2)	7.2(2)	11.1(4)	0	0	3.62(12)
Se1	10.4(9)	10.4(9)	9.1(12)	0	0	5.2(4)
Se2	6.9(8)	6.9(8)	10.8(12)	0	0	3.5(4)
Sn1	2(6)	2(6)	3(10)	0	0	1(3)

Supplementary Table 6: Atomic parameters with fractional atomic coordinates and equivalent isotropic displacement parameters ($Å^2 \times 10^3$) for structure two ($Sn_{0.16(2)}Ta_{1.0(2)}Se_{2.0(2)}$). U_{eq} is defined as 1/3 of the trace of the orthogonalised U_{IJ} tensor. Atomic positions are fixed in final refinement; provided uncertainties for atomic coordinates are determined when the occupancies are refined.

**Atom**	**Wyckoff**	**Site**	**Occupancy**	**x**	**y**	**z**	$\mathbf{U_{eq}}$
Ta1	2*c*	$\bar{6}m2$	0.88(14)	2/3	1/3	3/4	16.8(8)
Ta2	2*b*	$\bar{6}m2$	0.11(14)	0	1	3/4	42(8)
Se1	4*e*	$3m.$	0.41(7)	0	1	0.6189(5)	18(2)
Se2	4*f*	$3m.$	0.46(9)	1/3	2/3	0.6188(5)	20(2)
Se3	4*f*	$3m.$	0.13(10)	2/3	1/3	0.6189(18)	29(8)
Sn1	4*f*	$3m.$	0.081(9)	1/3	2/3	0.5219(16)	11(6)

Supplemental Table 7: Anisotropic displacement parameters ($Å^2 \times 10^3$) for structure two ($Sn_{0.16(2)}Ta_{1.0(2)}Se_{2.0(2)}$). The anisotropic displacement factor exponent takes the form: $-2\pi^2[h^2a^{*2}U_{11}+2hka^*b^*U_{12}+\ldots]$.

**Atom**	$\mathbf{U_{11}}$	$\mathbf{U_{22}}$	$\mathbf{U_{33}}$	$\mathbf{U_{23}}$	$\mathbf{U_{13}}$	$\mathbf{U_{12}}$
Ta1	13.1(9)	13.1(9)	24.4(12)	0	0	6.5(4)
Ta2	50(10)	50(10)	25(10)	0	0	25(5)
Se1	18(2)	18(2)	18(3)	0	0	9.2(11)
Se2	16(2)	16(2)	28(4)	0	0	8.2(12)
Se3	33(10)	33(10)	20(12)	0	0	17(5)
Sn1	15(7)	15(7)	3(9)	0	0	7(4)

Supplementary Table 8: Atomic parameters with fractional atomic coordinates and equivalent isotropic displacement parameters (Å^2×10^3) for structure three ($Sn_{1.2}Ta_{1.0}Se_{1.9}$). U_{eq} is defined as 1/3 of the trace of the orthogonalised U_{IJ} tensor. Atomic positions are fixed in final refinement; provided uncertainties for atomic coordinates are determined when the occupancies are refined.

**Atom**	**Wyckoff**	**Site**	**Occupancy**	**x**	**y**	**z**	$\mathbf{U_{eq}}$
Ta1	4*a*	*mm*2	0.95	1/2	1/2	0.2278(9)	25.1(15)
Ta2	8*d*	.*m*.	0.05	0.539(2)	1/2	0.254(10)	10(8)
Se1	8*d*	.*m*.	1	0.5669(4)	1/2	0.5598(12)	27(2)
Sn1	16*e*	1	0.15	0.6917(17)	0.752(14)	0.311(7)	58(10)
Sn2	8*d*	.*m*.	0.165	0.694(2)	1	0.306(9)	55(12)
Sn3	8*d*	.*m*.	0.16	0.693(2)	1/2	0.311(10)	59(13)

Supplementary Table 9: Anisotropic displacement parameters (Å^2×10^3) for structure three ($Sn_{1.2}Ta_{1.0}Se_{1.9}$). The anisotropic displacement factor exponent takes the form: -$2\pi^2[h^2a^{*2}U_{11}+2hka^*b^*U_{12}+\ldots]$.

**Atom**	$\mathbf{U_{11}}$	$\mathbf{U_{22}}$	$\mathbf{U_{33}}$	$\mathbf{U_{23}}$	$\mathbf{U_{13}}$	$\mathbf{U_{12}}$
Ta1	47(3)	18(2)	10.6(19)	0	0	0
Se1	41(5)	22(4)	17(4)	0	-1(3)	0

Supplementary Table 10: Phase data for $Sn_{0.36}TaSe_{2.11}$ (theoretical).

**Phase Data**	
Empirical formula	$Sn_{0.36}TaSe_{2.11}$
Formula weight	401.65
Crystal system	Trigonal
Space group	$R\bar{3}$
a/Å	3.44170
b/Å	3.44170
c/Å	18.8554
Volume/$Å^3$	193.425

Supplementary Table 11: Atomic parameters for $Sn_{0.36}TaSe_{2.11}$ (theoretical).

**Atom**	**Wyckoff**	**Site**	**Occupancy**	**x/a**	**y/b**	**z/c**
Ta1	$3b$	$-3.$	0.91	1.00	0	1/2
Ta2	$6c$	3.	0.045	1/3	2/3	0.25679
Se1	$6c$	3.	1/2	1/3	2/3	0.41244
Se2	$6c$	3.	1/2	2/3	1/3	0.41229
Sn1	$6c$	3.	0.179	1/3	2/3	0.34575

Supplementary Table 12: Phase data for $Sn_{0.17}TaSe_2$ (theoretical).

**Phase Data**	
Empirical formula	$Sn_{0.17}TaSe_2$
Formula weight	369.40
Crystal system	Trigonal
Space group	$R3m$
a/Å	3.440
b/Å	3.440
c/Å	18.860
Volume/Å^3	193.3

Supplementary Table 13: Atomic parameters for $Sn_{0.17}TaSe_2$ (theoretical).

**Atom**	**Wyckoff**	**Site**	**Occupancy**	**x/a**	**y/b**	**z/c**
Ta1	$3a$	$3m$	1	1/3	2/3	0.21204
Se1	$3a$	$3m$	1	2/3	1/3	0.30007
Se2	$3a$	$3m$	1	2/3	1/3	0.12355
Sn1	$3a$	$3m$	0.1608	1.00	1.00	0.3638

Supplementary Table 14: DFT total energy calculations (in eV per formula unit).

	**Structure One:** $Sn_{1/6}TaSe_2$	**Structure Two:** $Sn_{1/4}TaSe_2$	**Structure Three:** $SnTaSe_2$
Directly from CIF	-673.7587234	-681.1356365	-753.9806881
Relaxed Atomic Positions	-674.3612183	-682.2179708	-754.8909198
Relaxed Atomic Positions and Cell	-674.4111384	-682.3160147	-754.9292509

Supplementary Table 15: Atomic positions for DFT calculations for structure one (cell: a = 3.4409, b = 6.8818, c = 18.997 Å).

**Identity**	**x/a**	**y/b**	**z/c**
Se	0.33331	-0.16666	0.79658
Se	0.66669	-0.33335	0.46325
Se	0.66669	0.16666	0.46325
Se	1.00000	0.00000	0.12992
Se	0.33331	0.33335	0.79658
Se	1.00000	-0.50000	0.12992
Se	0.33331	-0.16666	0.62094
Se	0.33331	0.33335	0.62094
Se	1.00000	0.00000	0.95427
Se	1.00000	-0.50000	0.95427
Se	0.66669	0.16666	0.28761
Se	0.66669	-0.33335	0.28761
Sn	0.33331	-0.16666	0.19043
Ta	1.00000	-0.50000	0.70881
Ta	0.33331	-0.16666	0.37548
Ta	0.66669	0.16666	0.04215
Ta	1.00000	0.00000	0.70881
Ta	0.33331	0.33335	0.37548
Ta	0.66669	-0.33335	0.04215

Supplementary Table 16: Optimized atomic positions for DFT calculations for structure one (cell: a = 3.3942, b = 6.8123, c = 20.2197 Å).

**Identity**	**x/a**	**y/b**	**z/c**
Se	0.34021	-0.16025	0.78919
Se	0.65135	-0.34819	0.48069
Se	0.64756	0.14801	0.48268
Se	1.03513	0.03363	0.09759
Se	0.33989	0.33944	0.78920
Se	1.02893	-0.47263	0.09572
Se	0.32686	-0.17280	0.62554
Se	0.32728	0.32762	0.62575
Se	1.01838	0.01776	0.93243
Se	1.01840	-0.48227	0.93432
Se	0.64472	0.14602	0.31793
Se	0.62724	-0.37141	0.31894
Sn	0.32108	-0.17738	0.21432
Ta	1.00090	-0.49915	0.70736
Ta	0.31495	-0.18419	0.39978
Ta	0.68735	0.18633	0.01358
Ta	1.00003	-0.00002	0.70717
Ta	0.30591	0.30679	0.40157
Ta	0.69713	-0.30396	0.01469

Supplementary Table 17: Atomic positions for DFT calculations for structure two (cell: a = 3.4404, b = 6.8808, c = 12.6239 Å).

**Identity**	**x/a**	**y/b**	**z/c**
Se	0	0.5	0.38055
Se	0	0.5	0.61945
Se	0	0	0.61945
Se	0	0	0.38055
Se	0.66669	0.166655	0.11883
Se	0.66667	0.666665	0.11883
Se	0.33331	0.333345	0.88117
Se	0.33333	0.833335	0.88117
Sn	0.66669	0.666655	0.4806
Ta	0.33333	0.833335	0.25
Ta	0.66667	0.666665	0.75
Ta	0.66669	0.166655	0.75
Ta	0.33331	0.333345	0.25

Supplementary Table 18: Optimized atomic positions for DFT calculations for structure two (cell: a = 3.4137, b = 7.0469, c = 13.1708 Å).

**Identity**	**x/a**	**y/b**	**z/c**
Se	0.14554	0.64550	0.32658
Se	-0.11990	0.38014	0.67044
Se	-0.10924	-0.10918	0.63302
Se	0.13488	0.13481	0.36400
Se	0.81149	0.31142	0.08534
Se	0.80494	0.80487	0.12333
Se	0.22072	0.22077	0.87369
Se	0.21416	0.71423	0.91168
Sn	0.51280	0.51282	0.49850
Ta	0.52960	1.02954	0.22709
Ta	0.60643	0.60647	0.77880
Ta	0.49606	-0.00389	0.76992
Ta	0.41921	0.41916	0.21821

Supplementary Table 19: Atomic positions for DFT calculations for structure three (cell: a = 24.774, b = 3.4226, c = 6.023 Å).

**Identity**	**x/a**	**y/b**	**z/c**
Se	0.43298	0.50000	0.66606
Se	0.06702	0.00000	0.66606
Se	0.93298	0.50000	0.16606
Se	0.93298	0.00000	0.66606
Se	0.56702	0.50000	0.66606
Se	0.43298	0.00000	0.16606
Se	0.56702	0.00000	0.16606
Se	0.06702	0.50000	0.16606
Sn	0.80597	0.27637	0.91658
Sn	0.69403	0.27637	0.41658
Sn	0.19403	0.27637	0.91658
Sn	0.32001	0.50000	0.38236
Ta	0.00000	0.50000	0.83464
Ta	0.50000	0.00000	0.83464
Ta	0.50000	0.50000	0.33464
Ta	0.00000	0.00000	0.33464

Supplementary Table 20: Optimized atomic positions for DFT calculations for structure three (cell: a = 25.1883, b = 3.3611, c = 5.9094 Å).

**Identity**	**x/a**	**y/b**	**z/c**
Se	0.50077	0.58859	0.66250
Se	-0.00212	-0.04836	0.61924
Se	0.86565	0.37147	0.13268
Se	0.86906	-0.12343	0.63532
Se	0.63612	0.66968	0.64081
Se	0.50385	0.08943	0.13538
Se	0.63271	0.16447	0.14373
Se	0.00107	0.45257	0.14633
Sn	0.74848	0.22569	1.13936
Sn	0.75324	-0.18439	0.63903
Sn	0.07685	0.49640	0.82901
Sn	0.42484	0.54468	0.35068
Ta	-0.06640	0.41384	0.81122
Ta	0.56952	0.12935	0.80142
Ta	0.56815	0.62728	0.32330
Ta	-0.06775	-0.08816	0.28913

References

(1) Dolomanov, O. V.; Bourhis, L. J.; Gildea, R. J.; Howard, J. A.; Puschmann, H. OLEX2: A Complete Structure Solution, Refinement and Analysis Program. *J. Appl. Cryst.* **2009**, *42*, 339–341, DOI: 10.1107/S0021889808042726.

(2) Sheldrick, G. M. SHELXT - Integrated Space-Group and Crystal-Structure Determination. *Acta Cryst. A* **2015**, *71*, 3–8, DOI: 10.1107/S2053273314026370.

(3) Palatinus, L.; Chapuis, G. SUPERFLIP - A Computer Program for the Solution of Crystal Structures by Charge Flipping in Arbitrary Dimensions. *J. Appl. Cryst.* **2007**, *40*, 786–790, DOI: 10.1107/S0021889807029238.

(4) Petrícek, V.; Dušek, M.; Palatinus, L. Crystallographic Computing System JANA2006: General Features. *Z. Kristallogr.* **2014**, *229*, 345–352, DOI: 10.1515/zkri-2014-1737.

(5) Sheldrick, G. M. Crystal Structure Refinement with SHELXL. *Acta Cryst. C* **2015**, *71*, 3–8, DOI: 10.1107/S2053229614024218.

(6) Momma, K.; Izumi, F. VESTA 3 for Three-Dimensional Visualization of Crystal, Volumetric and Morphology Data. *J. Appl. Cryst.* **2011**, *44*, 1272–1276, DOI: 10.1107/S0021889811038970.

(7) Talantsev, E. F.; Crump, W. P.; Island, J. O.; Xing, Y.; Sun, Y.; Wang, J.; Tallon, J. L. On the Origin of Critical Temperature Enhancement in Atomically Thin Superconductors. *2D Mater.* **2017**, *4*, 025072, DOI: 10.1088/2053-1583/aa6917.

(8) Giannozzi, P. et al. QUANTUM ESPRESSO: A Modular and Open-Source Software Project for Quantumsimulations of Materials. *J. Phys. Condens. Matter.* **2009**, *21*, 395502, DOI: 10.1088/0953-8984/21/39/395502.

(9) Giannozzi, P. et al. Advanced Capabilities for Materials Modelling with

Quantum ESPRESSO. *J. Phys. Condens. Matter.* **2017**, *29*, 465901, DOI: 10.1088/1361-648X/AA8F79.

(10) Jollet, F.; Torrent, M.; Holzwarth, N. Generation of Projector Augmented-Wave Atomic Data: A 71 Element Validated Table in the XML Format. *Comput. Phys. Commun.* **2014**, *185*, 1246–1254, DOI: 10.1016/J.CPC.2013.12.023.

(11) Perdew, J. P.; Ruzsinszky, A.; Csonka, G. I.; Vydrov, O. A.; Scuseria, G. E.; Constantin, L. A.; Zhou, X.; Burke, K. Restoring the Density-Gradient Expansion for Exchange in Solids and Surfaces. *Phys. Rev. Lett.* **2008**, *100*, 136406, DOI: 10.1103/PhysRevLett.100.136406.

(12) van Setten, M. J.; Giantomassi, M.; Bousquet, E.; Verstraete, M. J.; Hamann, D. R.; Gonze, X.; Rignanese, G. M. The PseudoDojo: Training and Grading a 85 Element Optimized Norm-Conserving Pseudopotential Table. *Comput. Phys. Commun.* **2018**, *226*, 39–54, DOI: 10.1016/J.CPC.2018.01.012.

(13) Bradley, C.; Cracknell, A. *The Mathematical Theory of Symmetry in Solids: Representation Theory for Point Groups and Space Groups*; Oxford University Press, 2010.

(14) Gonze, X. et al. The ABINIT project: Impact, Environment and Recent Developments. *Comput. Phys. Commun.* **2020**, *248*, 107042, DOI: 10.1016/J.CPC.2019.107042.

(15) Romero, A. H. et al. ABINIT: Overview and Focus on Selected Capabilities. *J. Chem. Phys.* **2020**, *152*, 124102, DOI: 10.1063/1.5144261.

(16) Hartwigsen, C.; Goedecker, S.; Hutter, J. Relativistic Separable Dual-Space Gaussian Pseudopotentials from H to Rn. *Phys. Rev. B* **1998**, *58*, 3641, DOI: 10.1103/PhysRevB.58.3641.

(17) Bruker AXS Inc XPREP 2014 (X-Ray Data Preparation and Reciprocal Space Exploration Program). 2014.

(18) Solar, M.; Trapp, N.; Wörle, M. Zürich Space Group Helper. https://gitlab.ethz.ch/trappn/zsgh.

(19) Albright, A. L.; White, J. M. *Methods in Molecular Biology*; Humana Press Inc., 2013; Vol. 1055; pp 149–162, DOI: 10.1007/978-1-62703-577-4_11.

(20) Linden, A. Best Practice and Pitfalls in Absolute Structure Determination. *Tetrahedron Asymmetry* **2017**, *28*, 1314–1320, DOI: 10.1016/j.tetasy.2017.07.010.

(21) Flack, H. D.; Bernardinelli, G.; Clemente, D. A.; Linden, A.; Spek, A. L. Centrosymmetric and Pseudo-Centrosymmetric Structures Refined as Non-Centrosymmetric. *Acta Cryst. B* **2006**, *62*, 695–701, DOI: 10.1107/S0108768106021884.

(22) Hooft, R. W.; Straver, L. H.; Spek, A. L. Determination of Absolute Structure Using Bayesian Statistics on Bijvoet differences. *J. Appl. Cryst.* **2008**, *41*, 96–103, DOI: 10.1107/S0021889807059870.

(23) Flack, H. D. Absolute-Structure Reports. *Acta Cryst. C* **2013**, *69*, 803–807, DOI: 10.1107/S0108270113014789.

(24) Sheldrick, G. M. TWINABS. 2012.

(25) Le Page, Y. Computer Derivation of the Symmetry Elements Implied in a Structure Description. *J. Appl. Cryst.* **1987**, *20*, 264–269, DOI: 10.1107/S0021889887086710.

(26) Le Page, Y. MISSYM 1.1 – a Flexible New Release. *J. Appl. Cryst.* **1988**, *21*, 983–984, DOI: 10.1107/S0021889888007022.

(27) Spek, A. L. Single-Crystal Structure Validation with the Program PLATON. *J. Appl. Cryst.* **2003**, *36*, 7–13, DOI: 10.1107/S0021889802022112.

(28) Guzei, I.; Herbst-Irmer, R.; Munyaneza, A.; Darkwa, J. Detailed Example of the Identification and Crystallographic Analysis of a Pseudo-Merohedrally Twinned Crystal. *Acta Cryst. B* **2012**, *68*, 150–157, DOI: 10.1107/S0108768112002728.

(29) Luo, H.; Xie, W.; Tao, J.; Inouec, H.; Gyenis, A.; Krizan, J. W.; Yazdani, A.; Zhu, Y.; Cava, R. J. Polytypism, Polymorphism, and Superconductivity in $TaSe_{2-x}Te_x$. *Proc. Natl. Acad. Sci. U. S. A.* **2015**, *112*, E1174–E1180, DOI: 10.1073/pnas.1502460112.

(30) Takahashi, N.; Shiojiri, M.; Enomoto, S. High Resolution Transmission Electron Microscope Observation of Stacking Faults of Molybdenum Disulphide in Relation to Lubrication. *Wear* **1991**, *146*, 107–123, DOI: 10.1016/0043-1648(91)90228-M.

(31) Shiojiri, M.; Isshiki, T.; Saijo, H.; Yabuuchi, Y.; Takahashi, N. Cross-Sectional Observations of Layer Structures and Stacking Faults in Natural and Synthesized Molybdenum Disulfide Crystals by High-Resolution Transmission Electron Microscopy. *J. Electron Microsc.* **1993**, *42*, 72–78, DOI: 10.1093/oxfordjournals.jmicro.a051018.

(32) Zhang, J.; Wang, L.; Lü, J.; Wang, Z.; Wu, H.; Zhu, G.; Wang, N.; Xue, F.; Zeng, X.; Zhu, L.; Hu, Y.; Deng, X.; Guan, C.; Yang, C.; Lin, Z.; Wang, P.; Zhou, B.; Lü, J.; Zhu, W.; Zhang, X.; Huang, Y.; Huang, W.; Peng, Y.; Duan, X. Interlayer Reconstruction Phase Transition in van der Waals Materials. *Nat. Mater.* **2025**, 1–8, DOI: 10.1038/s41563-024-02082-w.

(33) Shi, M.; Fan, K.; Li, H.; Pan, S.; Cai, J.; Zhang, N.; Li, H.; Wu, T.; Zhang, J.; Xi, C.; Xiang, Z.; Chen, X. Two-Dimensional Superconductivity and Anomalous Vortex Dissipation in Newly Discovered Transition Metal Dichalcogenide-Based Superlattices. *J. Am. Chem. Soc.* **2024**, *146*, 33413–33422, DOI: 10.1021/jacs.4c09248.

(34) Moulder, J.; Stickle, W.; Sobol, P.; Bomber, K. In *Handbook of X-Ray Photoelectron Spectroscopy*; Jill, C., King, R., Eds.; Physical Electronics, Inc., 1992.

(35) Rupp, H.; Weser, U. X-ray Photoelectron Spectroscopy of Some Selenium Containing Amino Acids. *Bioinorg. Chem.* **1975**, *5*, 21–32, DOI: 10.1016/S0006-3061(00)80217-3.

(36) Nakamura, T.; Yasuda, S.; Miyamae, T.; Nozoye, H.; Kobayashi, N.; Kondoh, H.; Nakai, I.; Ohta, T.; Yoshimura, D.; Matsumoto, M. Effective Insulating Properties of

Autooxidized Monolayers Using Organic Ditellurides. *J. Am. Chem. Soc.* **2002**, *124*, 12642–12643, DOI: 10.1021/ja0276671.

(37) Grilli, R.; Simpson, R.; Mallinson, C. F.; Baker, M. A. Comparison of Ar^+ Monoatomic and Cluster Ion Sputtering of Ta_2O_5 at Different Ion Energies, by XPS: Part 1 - Monoatomic Ions. *Surf. Sci. Spectra* **2014**, *21*, 50–67, DOI: 10.1116/11.20140701.

(38) Tsai, H.-S.; Liu, F.-W.; Liou, J.-W.; Chi, C.-C.; Tang, S.-Y.; Wang, C.; Ouyang, H.; Chueh, Y.-L.; Liu, C.; Zhou, S.; Woon, W.-Y. Direct Synthesis of Large-Scale Multilayer $TaSe_2$ on SiO_2/Si Using Ion Beam Technology. *ACS Omega* **2019**, *4*, 17536–17541, DOI: 10.1021/acsomega.9b02441.

(39) Chiang, S.; Wertheim, G.; DiSalvo, F. Many-Electron Screening in Narrow-Band Metals; XPS in Layered Dichalcogenides. *Solid State Commun.* **1976**, *19*, 75–78, DOI: 10.1016/0038-1098(76)91733-6.

(40) Hughes, H. P.; Pollak, R. A. Charge Density Waves in Layered Metals Observed by X-ray Photoemission. *Philos. Mag.* **1976**, *34*, 1025–1046, DOI: 10.1080/00318087608227726.

(41) Pollak, R. A.; Hughes, H. P. Charge Density Wave Phase Transitions Observed by X-ray Photoemission. *J. Phys. Colloq.* **1976**, *37*, C4–151–C4–155, DOI: 10.1051/jphyscol:1976423.

(42) Waldvogel, H.; Schärli, M. Angle-Resolved X-ray Photoelectron Spectroscopy Study from 1*T*-$TaSe_2$ and from 2*H*-$TaSe_2$ Single Crystals. *J. Electron Spectrosc. Relat. Phenom.* **1984**, *34*, 115–128, DOI: 10.1016/0368-2048(84)80037-7.

(43) Crawack, H.; Pettenkofer, C. Calculation and XPS Measurements of the Ta4f CDW Splitting in Cu, Cs and Li Intercalation Phases of 1*T*-TaX_2 (X=S, Se). *Solid State Commun.* **2001**, *118*, 325–332, DOI: 10.1016/S0038-1098(00)00488-9.

(44) Yan, Z.; Jiang, C.; Pope, T. R.; Tsang, C. F.; Stickney, J. L.; Goli, P.; Renteria, J.; Salguero, T. T.; Balandin, A. A. Phonon and Thermal Properties of Exfoliated $TaSe_2$ Thin Films. *J. Appl. Phys.* **2013**, *114*, 204301, DOI: 10.1063/1.4833250.

(45) Chia, X.; Ambrosi, A.; Lazar, P.; Sofer, Z.; Pumera, M. Electrocatalysis of layered Group 5 Metallic Transition Metal Dichalcogenides (MX_2 , M = V, Nb, and Ta; X = S, Se, and Te). *J. Mater. Chem. A* **2016**, *4*, 14241–14253, DOI: 10.1039/C6TA05110C.

(46) Tsoutsou, D.; Aretouli, K. E.; Tsipas, P.; Marquez-Velasco, J.; Xenogiannopoulou, E.; Kelaidis, N.; Aminalragia Giamini, S.; Dimoulas, A. Epitaxial 2D MoS_2 ($HfSe_2$) Semiconductor/2D $TaSe_2$ Metal van der Waals Heterostructures. *ACS Appl. Mater. Interfaces* **2016**, *8*, 1836–1841, DOI: 10.1021/acsami.5b09743.

(47) Shi, J.; Chen, X.; Zhao, L.; Gong, Y.; Hong, M.; Huan, Y.; Zhang, Z.; Yang, P.; Li, Y.; Zhang, Q.; Zhang, Q.; Gu, L.; Chen, H.; Wang, J.; Deng, S.; Xu, N.; Zhang, Y. Chemical Vapor Deposition Grown Wafer-Scale 2D Tantalum Diselenide with Robust Charge-Density-Wave Order. *Adv. Mater.* **2018**, *30*, 1804616, DOI: 10.1002/adma.201804616.

(48) Wang, M.; Zhang, L.; Huang, M.; Liu, Y.; Zhong, Y.; Pan, J.; Wang, Y.; Zhu, H. Morphology-Controlled Tantalum Diselenide Structures as Self-Optimizing Hydrogen Evolution Catalysts. *Energy Environ. Mater.* **2020**, *3*, 12–18, DOI: 10.1002/eem2.12052.

(49) Wang, H.; Chen, Y.; Zhu, C.; Wang, X.; Zhang, H.; Tsang, S. H.; Li, H.; Lin, J.; Yu, T.; Liu, Z.; Teo, E. H. T. Synthesis of Atomically Thin $1T$-$TaSe_2$ with a Strongly Enhanced Charge-Density-Wave Order. *Adv. Funct. Mater.* **2020**, *30*, 2001903, DOI: 10.1002/adfm.202001903.

(50) Chen, Y.; Ruan, W.; Wu, M.; Tang, S.; Ryu, H.; Tsai, H.-Z.; Lee, R. L.; Kahn, S.; Liou, F.; Jia, C.; Albertini, O. R.; Xiong, H.; Jia, T.; Liu, Z.; Sobota, J. A.; Liu, A. Y.; Moore, J. E.; Shen, Z.-X.; Louie, S. G.; Mo, S.-K.; Crommie, M. F. Strong Correlations

and Orbital Texture in Single-Layer 1*T*-$TaSe_2$. *Nat. Phys.* **2020**, *16*, 218–224, DOI: 10.1038/s41567-019-0744-9.

(51) Deng, Y.; Lai, Y.; Zhao, X.; Wang, X.; Zhu, C.; Huang, K.; Zhu, C.; Zhou, J.; Zeng, Q.; Duan, R.; Fu, Q.; Kang, L.; Liu, Y.; Pennycook, S. J.; Wang, X. R.; Liu, Z. Controlled Growth of 3*R* Phase Tantalum Diselenide and Its Enhanced Superconductivity. *J. Am. Chem. Soc* **2020**, *142*, 2948–2955, DOI: 10.1021/jacs.9b11673.

(52) Adam, M. L.; Liu, Z.; Moses, O. A.; Wu, X.; Song, L. Superconducting Properties and Topological Nodal Lines Features in Centrosymmetric $Sn_{0.5}TaSe_2$. *Nano Res.* **2021**, *14*, 2613–2619, DOI: 10.1007/s12274-020-3262-2.

(53) Ge, Y.; Wang, F.; Yang, Y.; Xu, Y.; Ye, Y.; Cai, Y.; Zhang, Q.; Cai, S.; Jiang, D.; Liu, X.; Liedberg, B.; Mao, J.; Wang, Y. Atomically Thin $TaSe_2$ Film as a High-Performance Substrate for Surface-Enhanced Raman Scattering. *Small* **2022**, *18*, 2107027, DOI: 10.1002/smll.202107027.

(54) Lawan Adam, M.; Buba Garba, I.; Alhaji Bala, A.; Aji Suleiman, A.; Muhammad Gana, S.; Lawan Adam, F. Tuning Superconductivity and Charge Density Wave Order in $TaSe_2$ through Pt intercalation. *Phys. Rev. B* **2023**, *107*, 104510, DOI: 10.1103/PhysRevB.107.104510.

(55) Liu, L.; Zemlyanov, D. Y.; Chen, Y. P. Small-Rotation-Angle Moiré Structures of 2*H* $TaSe_2$ Monolayers on Au(111). *Nanoscale* **2025**, *17*, 6474–6480, DOI: 10.1039/D4NR03398A.

(56) Sthioul, C.; Chernukha, Y.; Koussir, H.; Coinon, C.; Patriarche, G.; Troadec, D.; Thomas, L.; Roussel, P.; Grandidier, B.; Diener, P.; Wallart, X. Thickness Dependence in Phase Formation and Properties of $TaSe_2$ Layers Grown on $GaP(111)_B$. *ACS Appl. Mater. Interfaces* **2025**, *17*, 10027–10037, DOI: 10.1021/acsami.4c17204.

(57) Li, X. C.; Zhou, M. H.; Dong, C. Superconductivity Enhancement in $Ta_{1+x}Se_2$ with a Randomly Stacked Structure. *Supercond. Sci. Technol.* **2019**, *32*, 035001, DOI: 10.1088/1361-6668/aaf355.

(58) Yang, Y.; Fang, S.; Fatemi, V.; Ruhman, J.; Navarro-Moratalla, E.; Watanabe, K.; Taniguchi, T.; Kaxiras, E.; Jarillo-Herrero, P. Enhanced Superconductivity Upon Weakening of Charge Density Wave Transport in $2H$-TaS_2 in the Two-Dimensional Limit. *Phys. Rev. B* **2018**, *98*, 035203, DOI: 10.1103/PhysRevB.98.035203.

(59) Pervin, R.; Krishnan, M.; Rana, A. K.; Kannan, M.; Arumugam, S.; Shirage, P. M. Enhancement of Superconducting Critical Current Density by Fe Impurity Substitution in $NbSe_2$ Single Crystals and the Vortex Pinning Mechanism. *Phys. Chem. Chem. Phys.* **2017**, *19*, 11230–11238, DOI: 10.1039/C7CP00784A.

(60) Liu, X.; Xu, C.; Jiang, J.; Wang, H.; Liu, S.; Liu, G.; Zhu, Z.; Yuan, J.; Xia, W.; Wen, L.; Luo, J.; Luo, Y.; Wang, X.; Yu, N.; Cheng, P.; Chen, L.; Zhou, R.; Li, J.; Chen, Y.; Wu, S.; Qu, K.; Li, W.; Zhang, G.; Duan, C.; Chen, J.; Xi, X.; Yang, Z.; Liu, K.; Guo, Y. Sliding Two-Dimensional Superconductivity and Charge-Density-Wave State in a Bulk Crystal. **2025**, DOI: 10.48550/arXiv.2508.01241.

(61) Arumugam, S.; Krishnan, M.; Ishigaki, K.; Gouchi, J.; Pervin, R.; Selvan, G. K.; Shirage, P. M.; Uwatoko, Y. Enhancement of Superconducting Properties and Flux Pinning Mechanism on $Cr_{0.0005}NbSe_2$ Single Crystal under Hydrostatic Pressure. *Sci. Rep.* **2019**, *9*, 347, DOI: 10.1038/s41598-018-36672-x.

(62) Niu, R.; Li, J.; Zhen, W.; Xu, F.; Weng, S.; Yue, Z.; Meng, X.; Xia, J.; Hao, N.; Zhang, C. Enhanced Superconductivity and Critical Current Density Due to the Interaction of $InSe_2$ Bonded Layer in $(InSe_2)_{0.12}NbSe_2$. *J. Am. Chem. Soc.* **2024**, *146*, 1244–1249, DOI: 10.1021/jacs.3c09756.

(63) Husaníková, P.; Fedor, J.; Dérer, J.; Šoltýs, J.; Cambel, V.; Iavarone, M.; May, S. J.;

Karapetrov, G. Magnetization Properties and Vortex Phase Diagram of Cu_xTiSe_2 Single Crystals. *Phys. Rev. B* **2013**, *88*, 174501, DOI: 10.1103/PHYSREVB.88.174501/.
